# Recycling Properties of Iridium Nanoparticles Anchored on Graphene as Catalysts in Alcohol Oxidation

**DOI:** 10.1021/acsanm.5c02235

**Published:** 2025-05-30

**Authors:** David Ruiz-Almoguera, Santiago Martín, Iván Sorribes, Jose A. Mata

**Affiliations:** † Institute of Advanced Materials (INAM), 16748 Universitat Jaume I, Avda. Sos Baynat s/n, 12071 Castellón, Spain; ‡ Instituto de Nanociencia y Materiales de Aragón (INMA), CSIC-Universidad de Zaragoza, 50009 Zaragoza, Spain; § Departamento de Química Física, 16765 Universidad de Zaragoza, 50009 Zaragoza, Spain; ∥ Laboratorio de Microscopias Avanzadas (LMA), Universidad de Zaragoza, Edificio I+D+i, 50018 Zaragoza, Spain

**Keywords:** iridium nanoparticles, catalyst deactivation, catalyst reusability, graphene, supported catalysis, hybrid materials

## Abstract

In this paper, we describe the synthesis, characterization, and recycling properties of iridium nanoparticles (IrNPs) anchored on graphene (**IrNPs@GNPs**) as a catalyst for alcohol oxidation. The formation of this hybrid material (comprising metal nanoparticles on graphene) is achieved in a single step under mild conditions. Graphene serves not only as a support for metal nanoparticles but also plays a critical role in controlling nanoparticle growth and nucleation while enhancing stability by preventing sintering. The IrNPs exhibit a spherical morphology with a small average size distribution (2.1 nm). The **IrNPs@GNPs** is an efficient catalytic material in the conversion of alcohols to the corresponding carbonyls in a sustainable manner, as supported by quantitative sustainability metrics. Oxidation reactions proceed at room temperature, using water as the solvent and atmospheric oxygen as the terminal oxidant. We detail the catalytic activity, substrate scope, and reuse/recycling of this hybrid material. Furthermore, we identify the primary deactivation pathway of the catalyst and present a regeneration protocol that restores its initial activity. The reuse and recyclability of iridium nanoparticles on graphene represent a significant advancement in the sustainable application of iridium-based catalysts.

## Graphical abstract

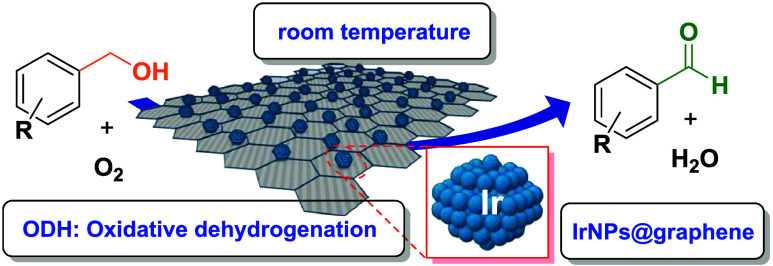

## 1 Introduction

Iridium-based nanomaterials are emerging as promising platforms for the rational design of materials with tailored properties relevant to a wide range of applications.^1–3^ For example, iridium nanoparticles have been employed in studying the kinetic model of the nanomaterial formation mechanism, particularly within the framework of the Finke–Watzky autocatalytic model.^4,5^ Iridium nanomaterials exhibit exceptional catalytic performance across various organic transformations, notably in hydrogenation reactions,^6–10^ as well as in water splitting reactions, where they serve primarily as anodes for the oxygen evolution reaction (OER).^11–13^ The catalytic activity achieved by iridium is not obtained using any other metals in many catalytic reactions, particularly in OER. However, the limited natural abundance and high cost of iridium force its replacement with more affordable/abundant metals, at least for developing industrial applications.^14^ This has driven the search for catalytic systems based on earth-abundant, non-critical metals, which represents a solid strategic research line.^15–17^ The objective is to explore the catalytic potential of these more available metals and to understand how their performance can be enhanced to match that of benchmark precious metals. This can be achieved through various strategies, including the use of appropriate supports, stabilizers, and organic ligands, or by employing single-atom catalysts to tailor the electronic and steric environment of active sites. In parallel, another approach to improve the sustainability of precious-metal-based catalysts involves the development of effective recycling and reuse methodologies, aiming to extend the lifespan and efficiency of these materials.

The use of graphene derivatives is particularly relevant for catalysis. The two-dimensional (2D) structure of graphene allows the allocation of active sites at the surface, directly predisposed for interaction with the substrate. The presence of active sites on the surface increases the efficiency of catalytic reactions by favoring the kinetics. In addition, graphene is based on abundant elements (mostly carbon and hydrogen), can display different compositions and sizes, and has the potential for straightforward functionalization with organic groups and metals. Our group has extensive experience in tailoring graphene-based materials for catalysis through the incorporation of metal complexes,^18–22^ organic functional groups,^23^ and metal nanoparticles.^24–27^

The oxidation of alcohols to aldehydes can be achieved via various stoichiometric reactions or catalytic procedures employing different oxidizing agents.^28–30^ Among these, the use of air as the oxidant is particularly notable from a sustainability perspective.^31–33^ One may argue that the reason is the abundance of air, but even more important is that the only byproduct is water.^34,35^ Employing iridium nanomaterials as heterogeneous catalysts offers a green approach to alcohol oxidation. The reaction proceeds at room temperature using atmospheric oxygen as the terminal oxidant and generating H_2_O as the sole byproduct. However, given the high cost and limited availability of iridium, improving the sustainability of such systems requires the development of efficient strategies for catalyst reuse and recycling. A key challenge in this context is the identification of deactivation pathways and the development of corresponding mitigation or reactivation protocols.

In this study, we present a rational and sustainable approach to alcohol oxidation using iridium nanoparticles anchored onto graphene. We focus on the catalyst’s reusability, investigating the mechanisms of deactivation and exploring strategies for catalyst reactivation to maintain catalytic performance over multiple cycles.

## 2 Experimental Section

### 2.1 Materials and Methods

Details of the equipment and procedures used for material characterization and product analysis are provided in the Supporting Information. Catalytic experiments were conducted under ambient conditions using air at atmospheric pressure.

### 2.2 Catalyst Preparation

#### 2.2.1 Synthesis of **IrNPs@GNPs**

To prepare the hybrid material **IrNPs@GNPs**, 500 mg of GNPs were dispersed in 100 mL of deionized water and sonicated for 30 min. Subsequently, a solution of IrCl_3_·3H_2_O (100 mg, 0.28 mmol) in 16 mL of deionized water was added, and the resulting suspension was stirred at ambient temperature for an additional 30 min. Following this, 16 mL of a 1 M NaBH_4_ solution (611.4 mg, 16.16 mmol) was introduced, and the mixture was left stirring at room temperature for 3 h. After the reaction was complete, the black precipitate was collected by filtration and sequentially washed with deionized water (3 × 100 mL) and acetone (1 × 100 mL). The resulting black powder corresponded to the **IrNPs@GNPs** hybrid. The iridium content was quantified by ICP-MS analysis, revealing a loading of 2.0 wt % iridium, which corresponded to 18% of the initial iridium used during the synthesis. **IrNPs@GNPs** can also be obtained starting from IrBr_3_·3H_2_O instead of the chloride salt, following the same procedure. Microscopy characterization and catalytic activity confirmed the exact nature and composition of the final material.

### 2.3 Catalytic Experiments

In a typical catalytic experiment, 0.5 mmol of the substrate and a specified amount of the catalyst were dispersed in 5 mL of deionized water and stirred at room temperature for 24 h. The reaction was performed in a Schlenk flask equipped with a reflux condenser and fitted with a septum and a needle to allow continuous access to atmospheric air. Conversion was determined by monitoring substrate consumption using GC/FID, while product yield was quantified by ^1^H NMR spectroscopy after extraction of organic products with dichloromethane and using 0.25 mmol of 1,3,5-trimethoxybenzene as an external standard. For water-soluble substrates, sodium formate (0.5–1.0 mmol) and D_2_O (100 μL) were added directly to the aqueous reaction mixture. After being stirred, a 0.6 mL aliquot was withdrawn and analyzed directly by ^1^H NMR spectroscopy.

#### 2.3.1 Reusability Experiments

Following the catalytic reaction, the **IrNPs@GNPs** hybrid material was recovered by filtration and subsequently washed with deionized water and dichloromethane. After the solid was dried with acetone, it was reused in a subsequent catalytic cycle without undergoing any regeneration treatment. Conversions and yields for each cycle were calculated by ^1^H NMR spectroscopy after product extraction with dichloromethane and using 1,3,5-trimethoxybenzene (0.25 mmol) as an external standard.

#### 2.3.2 Catalyst Activation

The spent **IrNPs@GNPs** material was activated by using a reductive treatment with molecular hydrogen. The spent **IrNPs@GNPs** material was placed in a crucible and inserted into a vertical tubular furnace. The sample was heated at 3 °C/min from room temperature to 300 °C, where the temperature was maintained for 2 h, under a continuous flow of hydrogen. After cooling to room temperature, the reactivated **IrNPs@GNPs** material was stored under nitrogen until the next use.

## 3 Results and Discussion

### 3.1 Synthesis and Characterization

Iridium nanoparticles anchored onto graphene nanoplatelets (**IrNPs@GNPs**) were synthesized in a single-step process (Scheme 1) by using the IrCl_3_·3H_2_O complex as the iridium source. The synthetic procedure involves a controlled reduction induced by sodium borohydride, which, under aqueous conditions, gradually releases hydrogen to act as the final reducing agent in a controlled manner.^36^ The iridium nanoparticles are directly immobilized on the surface of graphene nanoplatelets through interactions of d-orbitals of metal species with the delocalized π-electron cloud density of the carbonaceous material.^37^ The resulting hybrid material, consisting of iridium nanoparticles and graphene nanoplatelets, is obtained as an air-stable black powder.

**Scheme 1:**
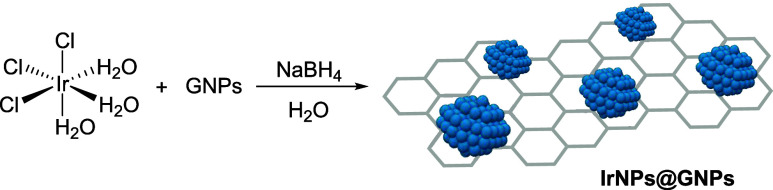
Synthesis of Iridium Nanoparticles Supported onto Graphene (GNPs)

The hybrid material **IrNPs@GNPs** was characterized by using a combination of microscopy, spectroscopy, and analytical techniques. Detailed characterization procedures and experimental details are provided in the Supporting Information. Morphological analysis via high-resolution transmission electron microscopy (HRTEM) reveals the two-dimensional structure of graphene nanoplatelets and the homogeneous distribution of the iridium nanoparticles across their surface (Figures 1 and S1–S6). The iridium nanoparticles exhibit a spherical morphology with a size histogram, indicating an average diameter of 2.12 ± 0.54 nm (Figure 1c). IrNPs are obtained in a small size and a relatively homogeneous size distribution. Energy-dispersive X-ray spectroscopy (EDX) confirms the presence of iridium at the bright spots of the scanning transmission electron microscopy (STEM) images, with carbon as the predominant element. Further elemental analysis was obtained by X-ray photoelectron spectroscopy (XPS; Figures 1d and S7–S11). The survey spectrum is dominated by carbon, oxygen, and iridium signals. Deconvolution analysis of the high-resolution core-level peak of Ir 4f reveals the presence of two doublets and a satellite peak at 66.4 eV. These doublets correspond to metallic Ir(0) and a surface layer of IrO_2_ in a 2:1 ratio, indicating partial iridium oxidation.^8,38^ The presence of IrO_2_ was further confirmed by deconvolution of the high-resolution peak of the O 1s spectrum, which displays a characteristic peak at 530.4 eV. Similarly, deconvolution of the high-resolution peak of the C 1s spectrum confirms the presence of C–O single bonds and C=O double bonds, characteristic of graphene-based materials, along with the predominant C–C peak indicative of the material’s graphitic nature.

**Figure 1:**
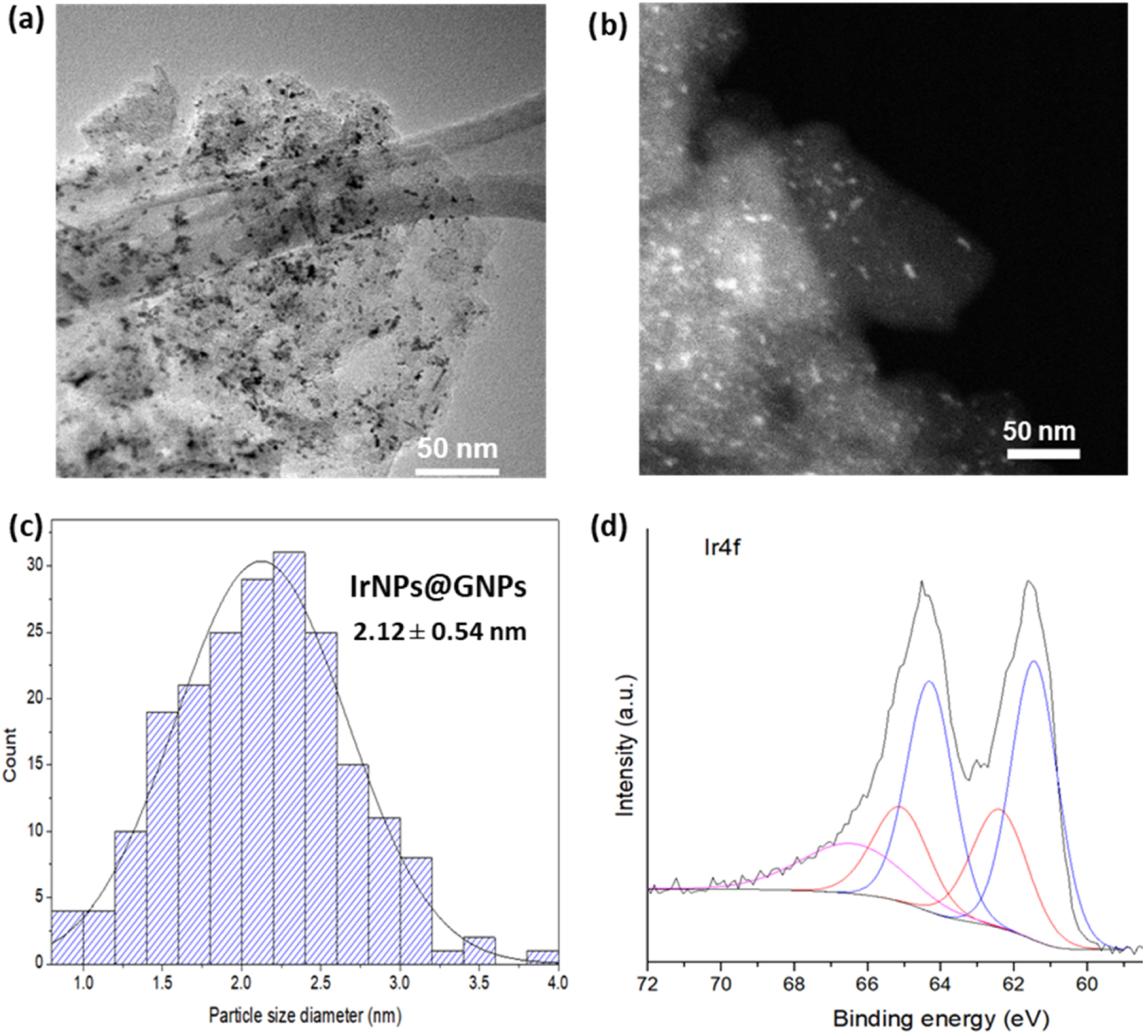
Characterization of **IrNPs@GNPs**. (a) HRTEM image, (b) STEM image, (c) size histogram (*N* = 200), and (d) XPS analysis for the core-level peak of Ir 4f. Peaks of Ir(0) are shown in blue at 61.4 and 64.3 eV, and those of IrO_2_ in red at 62.4 and 65.1 eV. Ratio Ir(0)/IrO_2_ = 2.

Raman spectroscopy provides key insights into the graphitic structure and the number of defects of carbon-based materials. The immobilization of iridium nanoparticles on the surface of graphene nanoplatelets induces only minor changes in the relative intensities of the graphitic (*I*_G_ at 1590 cm^–1^) and defect (*I*_D_ at 1350 cm^–1^) bands. The intensity ratio (*I*_D_/*I*_G_) for the hybrid material **IrNPs@GNPs** is 0.55, compared to 0.60 for pristine GNPs (Figures S12 and S13). These results indicate that the formation and immobilization of IrNPs do not significantly affect the graphitic character of the graphene nanoplatelets, as evidenced by Raman spectroscopy.

To further validate the synthesis methodology for anchoring IrNPs onto GNPs, an alternative iridium precursor, IrBr_3_·3H_2_O, was employed under identical reaction conditions. The resulting material, **Ir**_**Br**_ **NPs@GNPs**, was characterized and evaluated for catalytic activity, confirming the exact nature and composition of the final material (Figures S14–S17).

### 3.2 Catalytic Properties in the Transformation of Alcohol into Aldehydes

**IrNPs@GNPs** was tested as a catalyst for converting primary alcohols to aldehydes via oxidative dehydrogenation (ODH). We used oxygen from atmospheric air as a green and sustainable oxidation agent, with water as the sole byproduct. The compound used as a model substrate for the optimization of the catalytic conditions was *p*-methoxybenzyl alcohol (Table 1). First, we performed a series of control experiments. In the absence of a catalyst, no conversion or detectable aldehyde formation was observed, as confirmed by ^1^H NMR analysis after the extraction process (Table 1, entry 1). Similarly, no conversion was observed when only graphene nanoplatelets were used (Table 1, entry 2). These blank experiments are particularly relevant given the reported catalytic activity of graphene derivatives as carbocatalysts in various transformations, highlighting the potential non-innocent role of carbon supports.^23,39,40^ In our case, these results confirm that the iridium nanoparticles are catalytically active sites in the ODH of alcohols. Although the graphene support itself is not active in this transformation, it may still play a crucial role in the catalytic performance, as previously observed in systems involving gold and palladium nanoparticles anchored onto graphene.^21,24,26^ To assess the effect of the support, we compared the catalytic activity of unsupported iridium nanoparticles (IrNPs) to that of the supported hybrid material. Isolated IrNPs afforded modest yields (38%), whereas **IrNPs@GNPs** led to full conversion and quantitative yields under identical conditions (Table 1, entries 3 and 4). These findings underscore the significance of the support in designing efficient catalytic systems, likely due to its ability to stabilize metal nanoparticles and prevent sintering and/or deactivation. The heterogeneous/homogeneous nature of the transformation was proved using the poisoning mercury test. The results in the presence of mercury completely inhibited the formation of aldehyde, indicating the heterogeneous nature of the catalyst (Table 1, entry 5). The ODH of the alcohol reaction requires the presence of atmospheric oxygen as the final oxidant. In fact, when the reaction is carried out under a nitrogen atmosphere, the reaction barely proceeds, just confirming the requirement of oxygen (Table 1, entry 6). The use of air as an oxidant produces water as a byproduct. The production of water was confirmed by monitoring the ODH of *p*-methoxybenzyl alcohol in deuterated toluene (Figure S18).

**Table 1:**
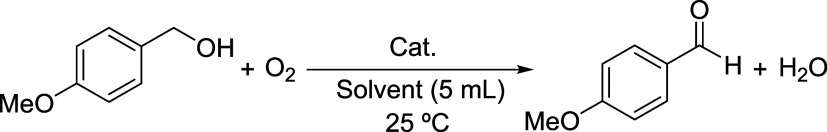
Reaction Optimization Using *p*-Methoxybenzyl Alcohol as a Model Substrate

entry	catalyst	atmosphere	solvent	conv. (%)^a^	yield (%)^a^	TON^d^	TOF (h^–1^)^e^
1		air	H_2_O				
2	**GNPs**	air	H_2_O				
3	**IrNPs**	air	H_2_O	38	36	120	n. d.
4	**IrNPs@GNPs**	air	H_2_O	100	97	323	227
5^b^	″	air	H_2_O				
6	″	N_2_	H_2_O	7	6	20	
7	**IrCl**_**3**_ **·3H**_**2**_ **O**	air	H_2_O				
8^c^	**IrNPs@GNPs**	air	CH_2_Cl_2_	20	20	67	n. d.
9^c^	″	air	toluene	60	60	200	121
10^c^	″	air	acetone	6	6	20	
11	″	air	CHCl_3_	14	14	47	
12	″	air	hexanes	45	42	140	
13	″	air	*n*-pentane	68	62	207	134
14	″	air	cyclohexane	7	6	20	
15	″	air	DMSO	6	6	20	
16	″	air	MeCN	6	5	17	
17	″	air	MeOH	20	18	60	
18	″	air	EtOH	19	19	63	
19	″	air	THF	9	8	27	
20	″	air	1,4-dioxane	10	9	30	

a Reaction conditions: *p*-methoxybenzyl alcohol (0.5 mmol), catalyst loading (0.5 mol % based on the total amount of Ir obtained by ICP-MS or 0.3 mol % considering only the atoms located at the surface of the nanoparticle), room temperature for 24 h, solvent (5 mL), aerobic conditions. Conversion and yield determined by ^1^H NMR using 1,3,5-trimethoxybenzene as an external standard.

b In the presence of Hg (0.1 mmol).

c Conversion and yield determined by gas chromatography using 0.5 mmol of anisole as an internal standard.

d TON: turnover number considering the number of atoms located at the surface of IrNPs.

e TOF: turnover frequency at 1 h considering the number of atoms located at the surface of IrNPs.

The catalytic activity of the iridium precursor IrCl_3_·3H_2_O, used in the synthesis of IrNPs, was also evaluated. The results demonstrated that this species is inactive in the ODH of alcohols, effectively ruling out the presence of unreacted IrCl_3_·3H_2_O on the graphene surface (Table 1, entry 7).

Subsequently, we investigated the performance of **IrNPs@GNPs** in a range of solvents (Table 1, entries 8–20). The reaction proceeded in the presence of various organic solvents. We have observed that when using solvents of low polarity, moderate activity was observed, whereas polar solvents led to diminished activity. Among all of the solvents tested, water afforded the best results, highlighting its suitability as a green and efficient reaction medium.

The ODH reactions were conducted using a catalyst loading of 0.5 mol % based on the total amount of iridium content, as determined by ICP-MS analysis. To more accurately assess the number of active sites, we estimated the proportion of surface atoms in the metal nanoparticles (Table S1 and Figure S19). The calculated dispersion, which represents the fraction of surface of atoms in a metal nanoparticle, was 59.9%, corresponding to an effective catalyst loading of 0.3 mol %. These initial catalytic results support the high catalytic efficiency of **IrNPs@GNPs** in the oxidative dehydrogenation of alcohols.

### 3.3 Kinetic Studies

We initially considered diffusional limitations as a potential factor affecting the efficiency of the heterogeneous catalysis system based on **IrNPs@GNPs**. In the first set of experiments, the same reaction was carried out under identical conditions but using different stirring speeds. The resulting reaction profiles were found to overlap, indicating that mass transfer does not significantly impact the reaction kinetics (Figure 2a). A complementary plot of conversion versus stirring speed at a fixed time (3 h) yielded consistent results (Figure 2b). These results are not unexpected for a graphene-based material where the active sites are located at the surface and are easily available to reach the substrates. Then, the catalytic properties of **IrNPs@GNPs** were further evaluated by monitoring the reaction evolution in the ODH process of *p*-methoxybenzyl alcohol at different catalyst loadings using the standard conditions (air, room temperature, and water as the solvent; Figure 2c). Reaction profiles showed the characteristic logarithmic curves of catalytic transformations without an induction period for all catalytic loadings tested. Quantitative yields of *p*-methoxybenzaldehyde were obtained using catalyst loadings within the 0.5–1 mol % range. Only in the case of 0.25 mol % catalyst loading, the yield achieved after 24 h is not quantitative (72%), but the reaction evolves gradually without observing catalyst deactivation. Catalyst stability was further assessed using a graphical method previously reported.^41,42^ This methodology involves plotting reaction progress as a function of the product of catalyst weight and time (*w* × *t*) for at least two distinct catalyst quantities. The superposition of these profiles indicates the absence of catalyst deactivation. In our study, we used four different catalyst mass loadings and observed the coincidence of all of the curves (Figure 2d). These results indicate that the **IrNPs@GNPs** catalytic material is not deactivated during the ODH of the alcohols.

**Figure 2:**
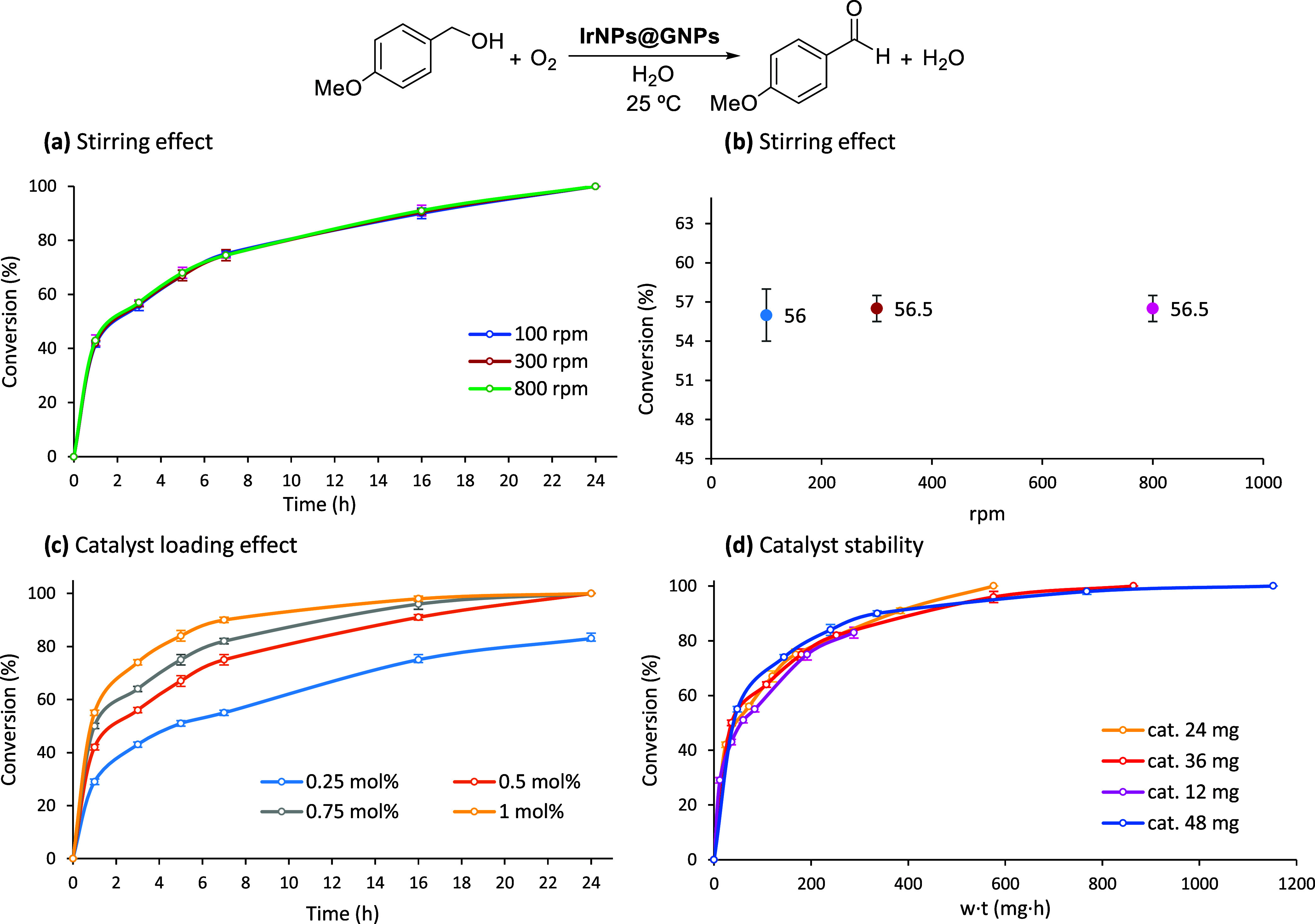
Reaction kinetics in the ODH reaction of benzyl alcohols. (a, b) Stirring effect, (c) catalyst loading effect (considering all of the metal amount and not only the atoms located at the surface), and (d) catalyst stability. Reaction conditions: *p*-methoxybenzyl alcohol (0.5 mmol), catalyst loading (0.5 mol % or as indicated), room temperature, solvent (5 mL), aerobic conditions.

Additionally, the experiments performed using different catalyst loadings allowed us to establish the reaction order in the catalysts using the graphical method of variable time normalization analysis (VTNA).^43,44^ The results suggest that oxidation of alcohols by **IrNPs@GNPs** is first-order dependence on catalyst concentration, confirming the direct involvement of **IrNPs@GNPs** in the reaction mechanism (Figures 3a, S20, and S21). Using the VTNA methodology, we also estimated the order of the reaction with respect to the substrate. For this, we performed the ODH of *p*-methoxybenzyl alcohol under standard conditions but with different initial concentrations of the substrate (Figures S22 and S23). Among all of the substrate orders scrutinized, the one where more data points were overlaid was 1, indicating the order of the reaction with respect to the substrate (Figure 3b).

**Figure 3:**
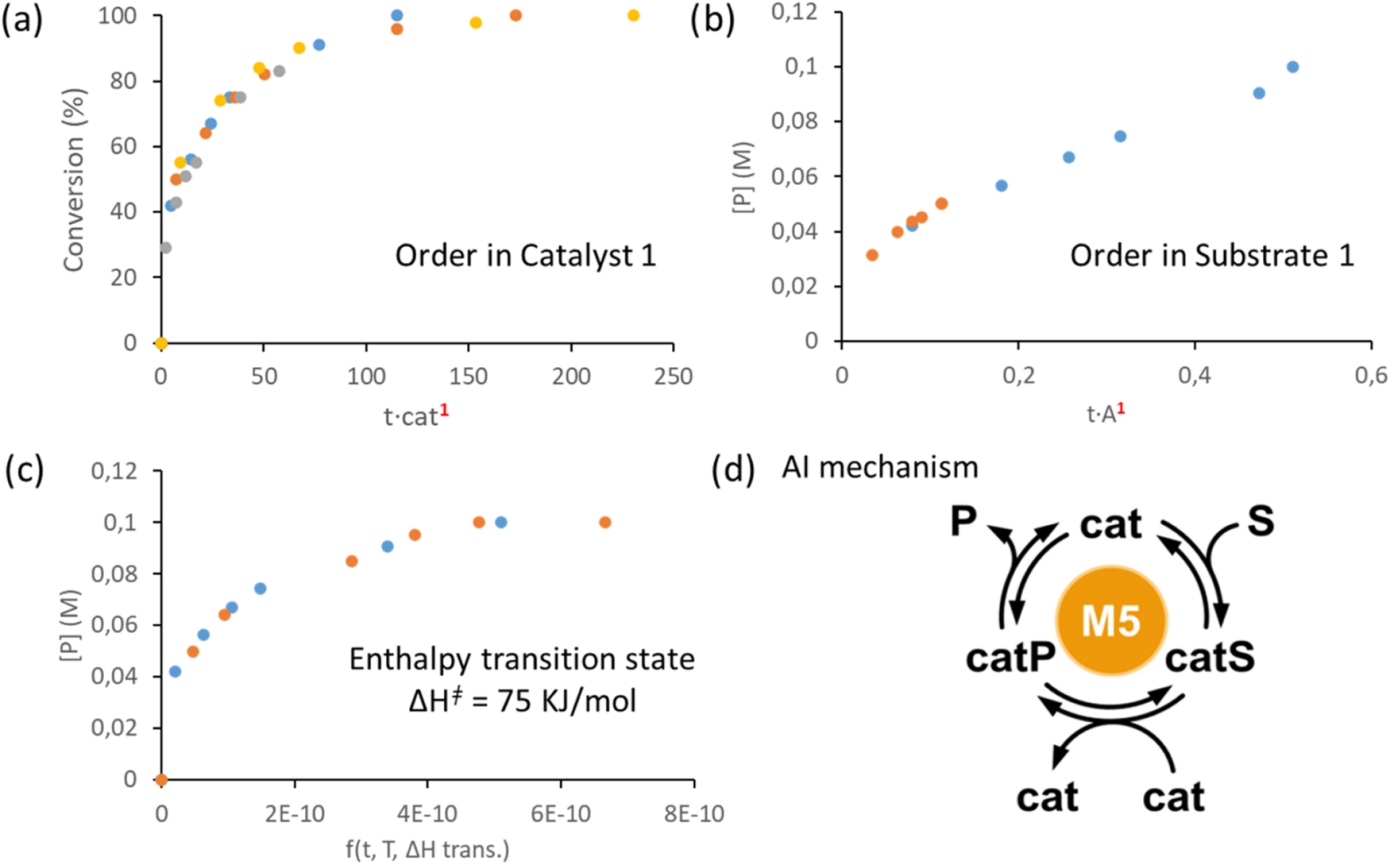
Kinetic studies: (a) determination of order with respect to the catalyst; the curves overlap at first-order, (b) determination of order with respect to the substrate; the curves also overlap at first-order, (c) estimation of the enthalpy transition state, and (d) mechanistic classification predicted by artificial intelligence (AI) algorithm.

We also obtained thermodynamic data of the ODH of alcohols by using a graphical method developed by Rivero-Crespo et al. (Figures S24–S27).^45^ The method is based on the Eyring–Polanyi equation, using a normalized time-scale function. The values of thermodynamic data are found in an iterative way, until the kinetic profiles overlay. The method provides an estimation of transition state enthalpy (Δ*H* ^*⧧*^), entropy (Δ*S* ^*⧧*^), and activation energy (*E*_a_). The value of transition state enthalpy (Δ*H* ^*⧧*^ = 75 kJ/mol) is low, as expected from a reaction that progresses at room temperature. The value of transition state entropy (Δ*S* ^*⧧*^ = −10 J/molK) is negative, suggesting an ordered transition state. With these data, the activation energy value at 25 °C is 77 kJ/mol, a low value that agrees with the experimental observations.

To further investigate the reaction mechanism, we employed a novel artificial intelligence (AI) approach recently developed by Burés and Larrosa.^46^ The method predicts one or more likely classes of reaction mechanisms using readily obtainable time–concentration profile data. (Figures 3d and S28). In our study, the AI algorithm identified a single dominant mechanistic class, denoted M5, belonging to the category of mechanisms involving bicatalytic steps. This prediction suggests that the oxidative dehydrogenation reaction proceeds via a pathway that requires the cooperative action of two distinct catalytic species. Such mechanistic insights may be crucial in guiding future optimization strategies and in the rational design of related catalytic systems.

To evaluate the substrate scope of the catalytic system, a series of benzyl alcohol derivatives were subjected to ODH under the optimized reaction conditions. Reaction progress was assessed through both substrate conversion and product yield. Conversion was determined by GC/FID using anisole as an internal standard (Figure S29), while yields were quantified by ^1^H NMR spectroscopy using 1,3,5-trimethoxybenzene as an external standard (Table 2). Overall, substrates bearing *para*-substituted electron-donating groups, such as *p*-methylbenzyl alcohol (Table 2, entry 1), *p*-methoxybenzyl alcohol (Table 2, entry 2), *p*-hydroxybenzyl alcohol (entry 8), and *p*-isopropylbenzyl alcohol (Table 2, entry 13), showed high conversions and excellent yields. Interestingly, halogenated derivatives such as *p*-chlorobenzyl alcohol (Table 2, entry 4) and *p*-bromobenzyl alcohol (Table 2, entry 6) also gave high conversions despite the electron-withdrawing nature of the substituents. The catalytic system also demonstrated compatibility with a range of functional groups, including nitro, hydroxyl, methylthio, and various alkyl substituents, providing moderate to good yields of the corresponding aldehydes (Table 2, entries 7–15). Notably, benzyl alcohols, containing fluorinated substituents, which are of particular interest for industrial applications, were efficiently converted to the corresponding aldehydes in good yields (Table 2, entries 16–18). ^1^H NMR analysis of crude reaction mixtures confirmed both the product yields and high selectivity toward the aldehyde products (Figures S30–S47). Importantly, all reactions were conducted at room temperature in water as the solvent and without the need for additional additives. These results highlight the versatility, efficiency, and sustainability of the **IrNPs@GNPs** hybrid catalyst for the selective oxidation of structurally diverse benzyl alcohols.

**Table 2:**
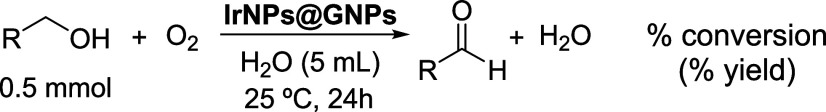

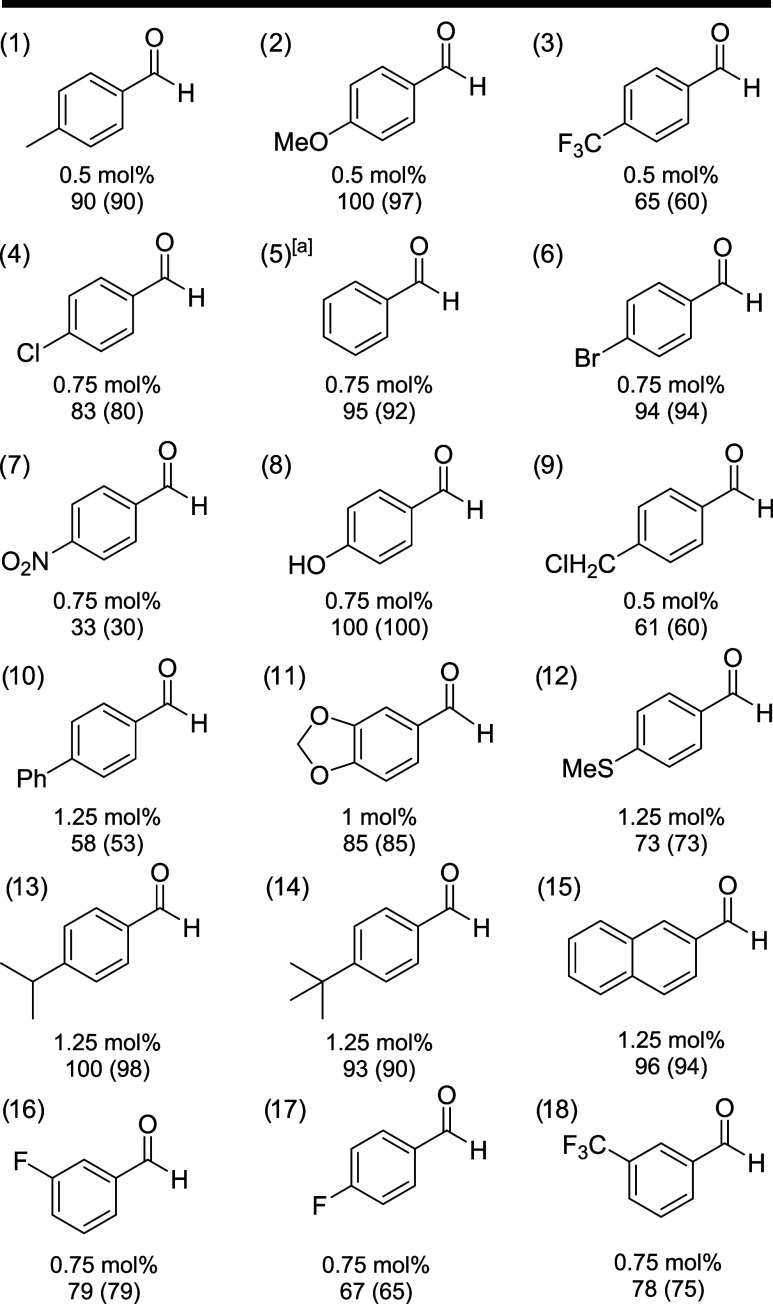
Reaction Scope in the ODH of Alcohols

a Conversion calculated with a gas chromatograph equipped with a flame ion detector (GC/FID) using anisole as an external standard. Yield calculated by ^1^H NMR spectroscopy using 1,3,5-trimethoxybenzene as an external standard.

The substrate scope was further expanded to include secondary alcohols with the aim of evaluating the catalytic versatility of **IrNPs@GNPs** (Table 3). In these reactions, the ODH of secondary alcohols leads to the formation of the corresponding ketones, as confirmed by ^1^H NMR analysis (Figures S48–S51). Compared with primary alcohols, we have observed that secondary alcohols exhibited lower reactivity under standard conditions. Notably, no conversion was observed when the reaction was conducted at room temperature. However, upon increasing the reaction temperature to 110 °C, quantitative yields of the corresponding ketones were obtained across the tested substrates. These findings demonstrate both the robustness and limitations of the **IrNPs@GNPs** catalytic system. While the catalyst is highly efficient and selective under mild conditions for primary alcohols, it requires elevated temperatures to achieve effective oxidation of secondary alcohols, highlighting the need for tailored conditions depending on substrate class.

**Table 3:**
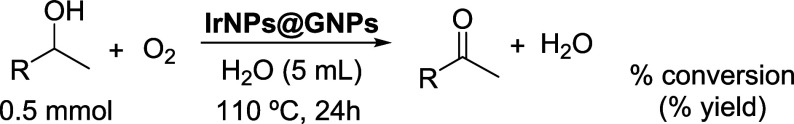

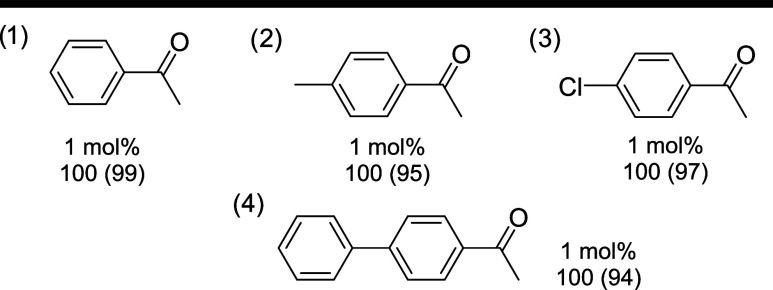
Evaluation of the ODH of Secondary Alcohols

a Conversion calculated with a GC/FID using anisole as an external standard. Yield calculated by ^1^H NMR spectroscopy using 1,3,5-trimethoxybenzene as an external standard.

### 3.4 Chemoselectivity Studies in the Oxidative Dehydrogenation of Alcohols

To investigate the chemoselectivity of the **IrNPs@GNPs** catalyst in the oxidative dehydrogenation (ODH) of alcohols, we performed a series of experiments using model compounds under standard reaction conditions with incomplete conversion to allow the identification of potential intermediates (Figures S52–S54). First, *p*-methoxybenzaldehyde was subjected to the reaction conditions to assess the possibility of overoxidation toward the corresponding carboxylic acid. The results show that no further overoxidation occurred under these conditions, even after a reaction time of 24 h (Figure 4a). A similar outcome was observed using a bifunctional substrate containing both an aldehyde and a benzylic alcohol group. In this case, selective oxidation of the alcohol to an aldehyde occurred, while the existing aldehyde functionality remained unaltered (Figure 4b). We further explored the selectivity of the catalyst in the presence of other potentially reactive functional groups. When the ODH of alcohols was conducted in the presence of alkenes, the alcohol was selectively oxidized to the aldehyde without any observable side reactions or transformations involving the alkene (Figure 4c). These results, together with the reaction scope, clearly demonstrate the excellent chemoselectivity of the **IrNPs@GNPs** catalyst, making it a promising system for the selective oxidation of alcohols in complex molecular environments.

**Figure 4:**
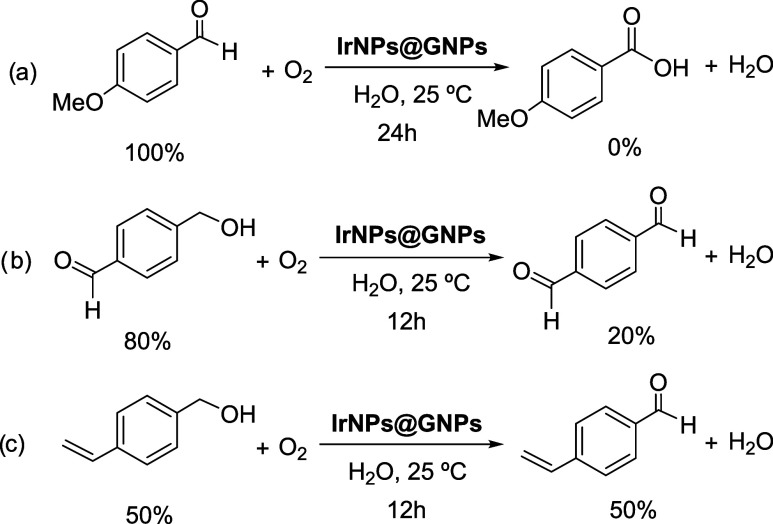
Chemoselective studies of **IrNPs@GNPs** in the ODH of alcohols. Product distribution (%) after the indicated time. Reaction conditions: substrate (0.5 mmol), catalyst loading (0.3 mol % based on surface Ir), room temperature, H_2_O (5 mL), aerobic conditions. (a) Aldehyde to carboxylic acid test, (b) Competitive experiment aldehyde versus benzyl alcohol, and (c) alkene hydrogenation test.

### 3.5 Reusability Experiments and Catalyst Deactivation

The long-term stability and reusability of the **IrNPs@GNPs** catalyst were evaluated by using *p*-methoxybenzyl alcohol as a model substrate under the standard reaction conditions (Figure 5). Reaction progress was monitored by sampling small aliquots (200 μL) prior to completion (3 h), allowing direct comparison of catalytic activity across recycling experiments and eliminating bias from cumulative catalyst usage. After each cycle, the solid catalyst was recovered by filtration, thoroughly washed with deionized water, dichloromethane, and acetone. Once the catalyst was air-dried, it was reused in a subsequent reaction without any activation process.

**Figure 5:**
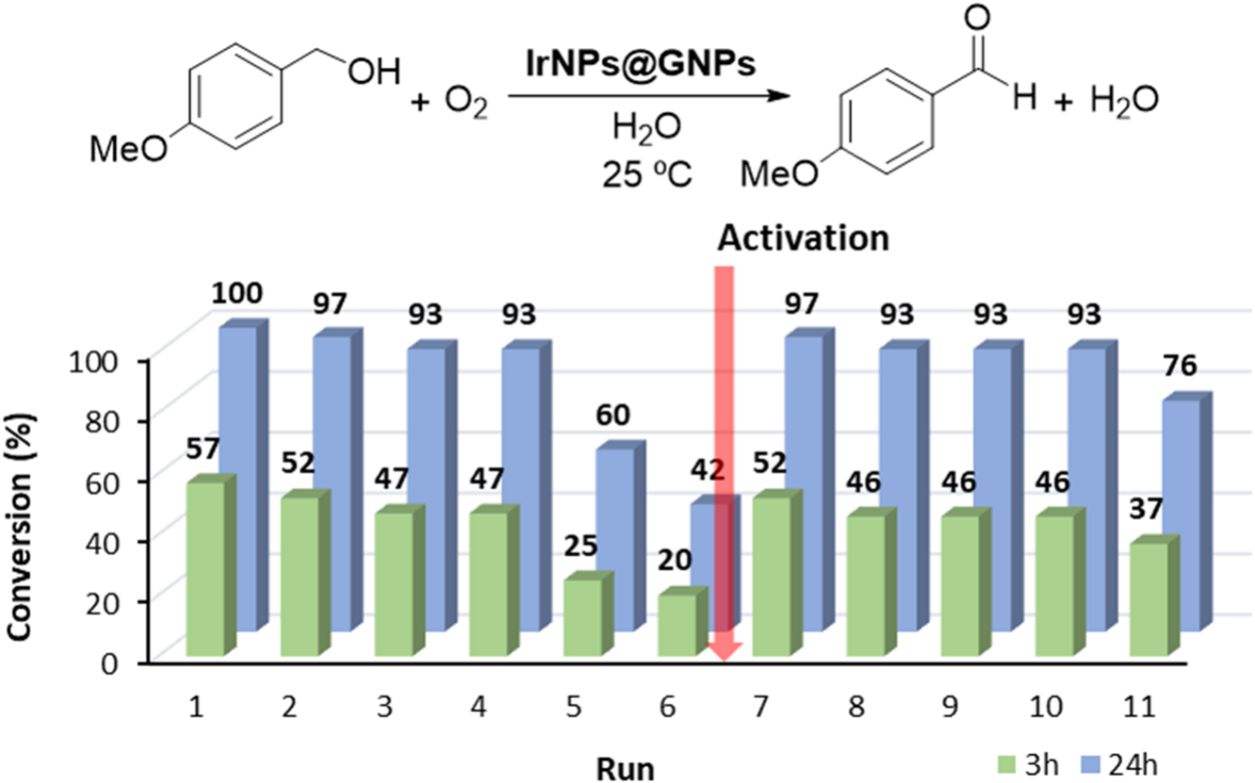
Recycling properties of **IrNPs@GNPs** in the ODH of alcohols. Reaction conditions: *p*-methoxybenzyl alcohol (0.5 mmol), catalyst loading (0.5 mol % based on the total amount of Ir obtained by ICP-MS), room temperature, solvent (5 mL), aerobic conditions.

The catalyst displayed consistent activity across the first four cycles, showing only a slight decrease in conversion at 3 h (from 57 to 47%) and at reaction completion (from 100 to 93%). However, in cycles 5 and 6, a pronounced drop in catalytic performance was observed, with conversion at 3 h falling to 25 and 20%, respectively. These results suggest a gradual deactivation process that accumulates over repeated catalytic cycles.

To elucidate the deactivation mechanism, we performed a hot filtration experiment. Deactivation caused by leaching of active species is a common process in supported catalysis. The hot filtration experiment was carried out in two parallel reactions: a control and the hot filtration experiment itself (Figure S55). The results showed that upon removal of the solid catalyst, the progress of the reaction was suppressed. This result was further confirmed by an ICP-MS analysis that excluded the presence of iridium species in the filtrate. These experiments point out the absence of active species in solution and confirm that catalytic transformation happens at the surface of graphene.

At this point, postreaction characterization of the spent catalyst (after run 6) was performed via HRTEM, Raman spectroscopy, and XPS (Figure 6). HRTEM and STEM images revealed no significant morphological changes in the IrNPs or the graphene support. The nanoparticle size remained unchanged (2.28 ± 0.47 nm), indicating that sintering was not responsible for deactivation. We have previously observed that the presence of graphene as support prevents metal nanoparticle agglomeration in the case of palladium and gold.^25,27^ Raman spectroscopy revealed a shift in the D/G band intensity ratio from 0.55 to 0.74, indicating an increase in structural defects (Figure S13).

**Figure 6:**
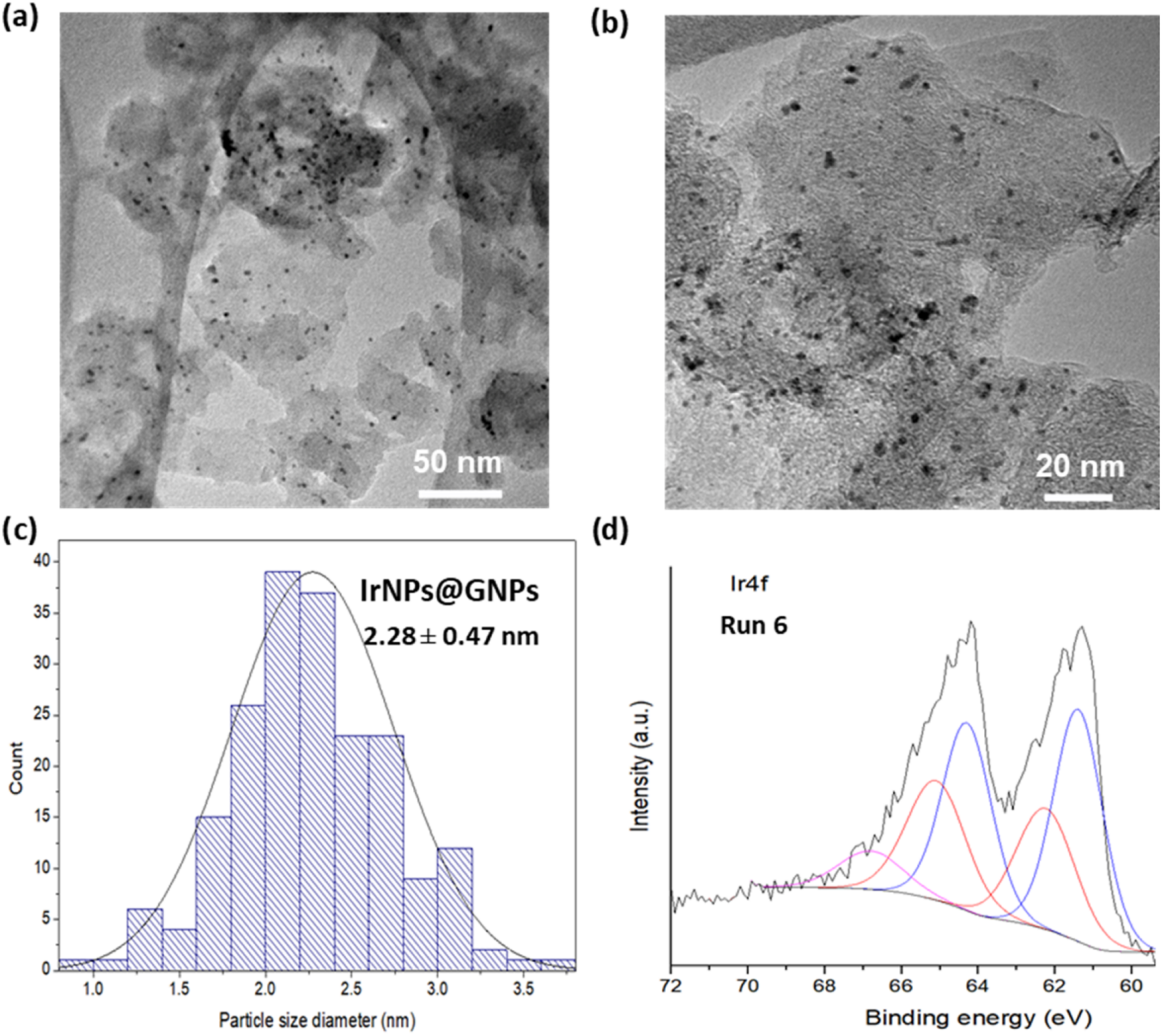
Characterization of spent **IrNPs@GNPs** after run 6. (a, b) HRTEM images at different magnifications, (c) size histogram (*N* = 200), and (d) XPS analysis for the core-level peak of Ir 4f. Peaks of Ir(0) are shown in blue at 61.4 and 64.3 eV, and those of IrO_2_ in red at 62.3 and 65.1 eV. Ratio Ir(0)/IrO_2_ = 1.5.

Characterization of the spent **IrNPs@GNPs** by XPS spectroscopy confirms the changes observed in catalysis and particularly in the active sites (Figures S8–S11). Deconvolution analysis of the high-resolution core-level peak of Ir 4f reveals an increment of the IrO_2_ peaks versus the parent material, indicating oxidation of IrNPs during the ODH of benzyl alcohols (Figure S9). These results suggest that deactivation of the hybrid material **IrNPs@GNPs** is produced by passivation of IrNPs caused by the formation of an external layer of IrO_2_. In view of these results, we sought to activate the catalyst under reduction conditions. For this, we treated the spent **IrNPs@GNPs** with hydrogen at a high temperature. XPS characterization of activated **IrNPs@GNPs** confirms a decrease in the peaks attributed to IrO_2_ (Figure S9) up to the initial values of the as-prepared catalyst. Then, activated **IrNPs@GNPs** was reused in subsequent catalytic runs. We observed that the activity was completely recovered after activation (run 7, conv. 52% at 3 h and 97% at 24 h). Interestingly, the catalyst activity is maintained for three additional runs (runs 8–10) before gradual deactivation resumed in cycle 11, again correlating with an increase in IrO_2_ content (Figure S9).

These findings demonstrate that **IrNPs@GNPs** is a robust and reusable catalytic system for the selective ODH of alcohols, with deactivation attributable to passivation via surface oxidation. Importantly, the activity can be fully restored by reductive treatment, highlighting the material’s potential for practical and sustainable catalytic applications.

### 3.6 Discussion of Sustainability Aspects

We evaluated the sustainability in the ODH of alcohol using benzyl alcohol as a model substrate (Scheme S1). Five key green chemistry metrics were assessed, including the toxicity of the chemicals involved (Table S2). Several factors underscore the environmentally friendly nature of our methodology. Notably, the oxidant used is molecular oxygen from air, an ideal green reagent that produces only water as a benign byproduct. The reaction is carried out in water, which is widely recognized as the most environmentally favorable solvent. Additionally, the transformation proceeds efficiently at room temperature, minimizing energy consumption and enhancing the process’s overall green credentials. While the use of iridium, a scarce metal, poses a limitation from a sustainability perspective, this challenge is mitigated by the demonstrated recyclability and reusability of the catalyst. Moreover, we developed a straightforward reactivation protocol that restores catalytic performance after deactivation, further extending the catalyst′s lifecycle.

Taken together, these features position our protocol as a sustainable strategy for alcohol oxidation (Table S3). The combination of a benign oxidant, a green solvent, mild reaction conditions, and a recyclable catalyst highlights a promising pathway toward the development of environmentally responsible catalytic methodologies. We hope these findings encourage the continued advancement of green chemistry in the design of efficient oxidation processes.

## 4 Conclusions

In this work, we developed a hybrid material comprising ligand-free iridium nanoparticles anchored onto the surface of graphene nanoplatelets **(IrNPs@GNPs)**. This heterogeneous system catalyzes the selective oxidation of benzyl alcohols to benzaldehydes under mild and sustainable conditions: room temperature, atmospheric oxygen as the oxidant, and water as the solvent. These green attributes position the methodology as a promising contribution to environmentally conscious chemical synthesis. The catalyst demonstrates excellent stability and performance over multiple reaction cycles. Upon extended use, gradual deactivation is observed, which has been mechanistically attributed to the formation of a passivating layer of iridium­(IV) oxide (IrO_2_) on the nanoparticle surface. Importantly, catalytic activity can be fully restored through a simple high-temperature hydrogen treatment, which reduces the IrO_2_ layer and regenerates the active IrNPs. This reversible deactivation and reactivation process underscores the robustness and reusability of **IrNPs@GNPs**, further enhancing its appeal for sustainable catalytic applications.

## Supplementary Material



## Notes

Additional characterization materials by XPS, Raman, and HRTEM; further details of experimental procedures; NMR characterization of organic products; and ESI-MS (PDF)
