# Supplementary material for: Recycling Properties of Iridium Nanoparticles Anchored on Graphene as Catalysts in Alcohol Oxidation
Source: ACS Appl Nano Mater. 2025 May 30;8(23):12342–52. doi: 10.1021/acsanm.5c02235 (PMC12172379; doi:10.1021/acsanm.5c02235)
Supplement: Supplementary file 1 [file an5c02235_si_001.pdf]

SUPPORTING INFORMATION

Recycling Properties of Iridium Nanoparticles Anchored on Graphene as Catalysts in Alcohol Oxidation

David Ruiz-Almoguera,[a] Santiago Martín,[b,c] Iván Sorribes[a] and Jose A. Mata[a]**

[a]Institute of Advanced Materials (INAM), Universitat Jaume I, Avda. Sos Baynat s/n, 12071, Castellón (Spain). Tel: +34 964387516. Email: isorribe@uji.es and jmata@uji.es

[b]Instituto de Nanociencia y Materiales de Aragón (INMA), CSIC-Universidad de Zaragoza, 50009, Zaragoza (Spain)

[c]Departamento de Química Física, Universidad de Zaragoza, 50009, Zaragoza (Spain) and Laboratorio de Microscopias Avanzadas (LMA). Universidad de Zaragoza, Edificio I+D+i. 50018, Zaragoza (Spain)

Contents

S1. Materials and methods 2

S2. Microscopy characterization (Transmission electron microscopy) 3

S3. X-ray photoelectron spectroscopy (XPS) 7

S4. Raman spectroscopy 10

S5. Synthesis and characterization of IrNPs@GNPs starting from $IrBr_3·3H_2O$ 12

S6. Detection of H_2O formation 15

S7. Estimation of surface atoms in IrNPs@GNPs nanoparticles 16

S8. Kinetic studies 17

S8.1 Order in catalyst 17

S8.2 Order in substrate 18

S8.3 Thermodynamic data 19

S8.4 Artificial intelligence (AI) mechanism proposal 23

S9. Gas chromatography spectra of organic compounds 24

S10. ^{1}H NMR spectra of crude reaction products 25

S11. ^{1}H NMR spectra of crude reaction products in ODH of secondary alcohols 42

S12. Chemoselectivity studies 46

S13. Hot filtration experiment 49

S14. Evaluation of green chemistry aspects 50

S15. Catalyst comparison data 52

S16. References 53

S1. Materials and methods

Reagents used in the preparation of catalyst were synthetic grade and used as received: $IrCl_3 \cdot 3H_2O$ (100%), $IrBr_3 \cdot 3H_2O$ (100%), $NaBH_4$ (99%). Deionized water was used as provided. Graphene Nanoplatelets 750 m^2/g (**GNPs**) was purchased (Sigma Aldrich) and used as received. Substrates used for catalytic experiments are commercially available and used as received.

Yield and conversion were determined by nuclear magnetic resonance (NMR). The NMR spectra were recorded on Bruker spectrometers operating at 300 or 400 MHz (^1H NMR) and referenced to $SiMe_4$ (δ in ppm and J in Hertz).

High-resolution images of transmission electron microscopy (HRTEM) and high-angle annular dark-field images (HAADF-STEM) of the samples were obtained using a Jem-2100 LaB6 (JEOL) transmission electron microscope couple with an INCA Energy TEM 200 (Oxford) energy dispersive X-Ray spectrometer (EDX) operating at 200 kV. To prepare the samples, a droplet of a methanol dispersion was dried on a carbon-coated copper grid.

The iridium content in the hybrid material was determined by inductively coupled mass spectrometry analysis (ICP-MS). A 15 mg sample goes under a digestion process in a Mars 6 iWave CEM microwave oven using nitric acid (69%) with a power of 1800 W at 210 °C during 25 minutes. The solid particles were removed by filtration and, after diluting to an adequate volume, ICP-MS analyses were recorded on an Agilent 7500 CS instrument in duplicate.

Raman spectra were recorded at room temperature using a 532 nm laser excitation focused through a WITec GmbH Raman spectrophotometer equipped with a CCD detector. XPS spectra were recorded on a Kratos AXIS ultra DLD spectrometer equipped with an Al Kα X-ray monochromatic source (1486.6 eV) and using 20 eV as pass energy. Binding energies were calibrated according to the C1s peak at 284.6 eV.

S2. Microscopy characterization (Transmission electron microscopy)

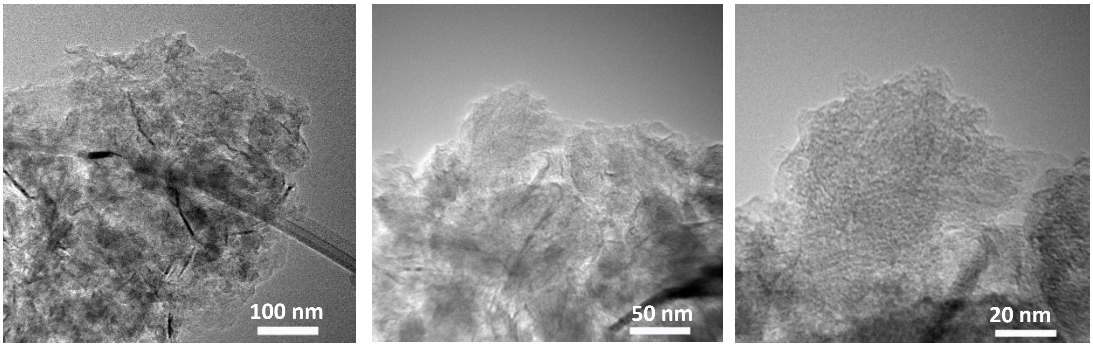

Figure S1 HRTEM images of **GNPs** at various magnifications.

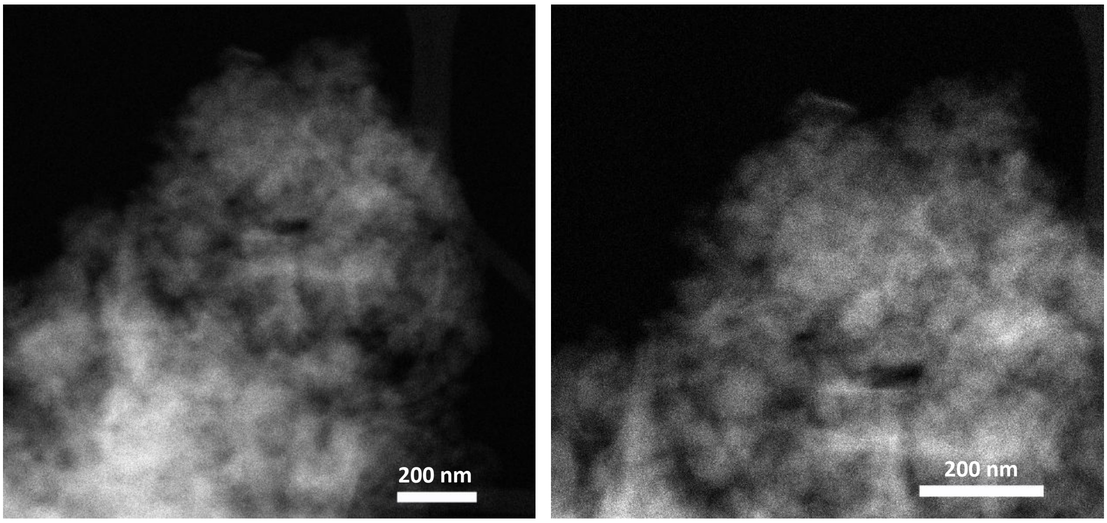

Figure S2 STEM images of **GNPs**.

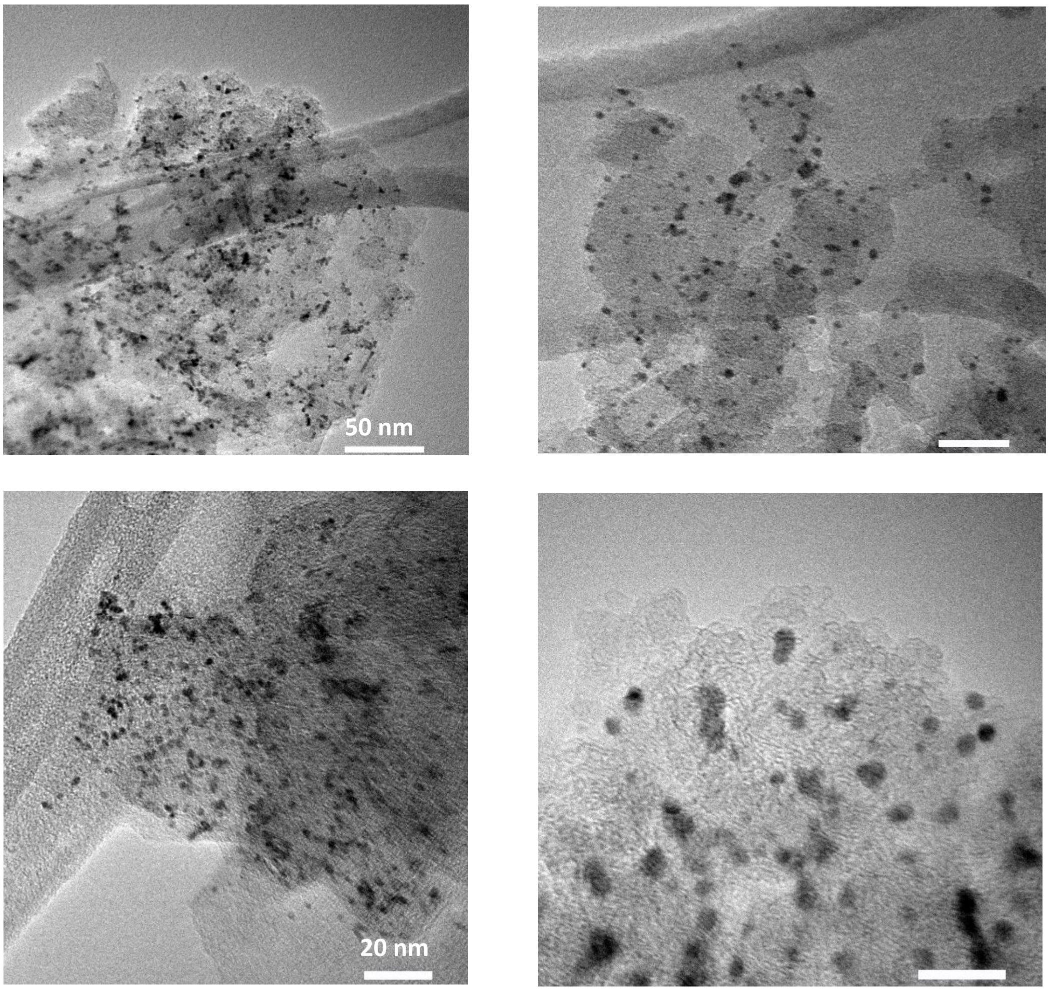

Figure S3 HRTEM images of **IrNPs@GNPs** at various magnifications.

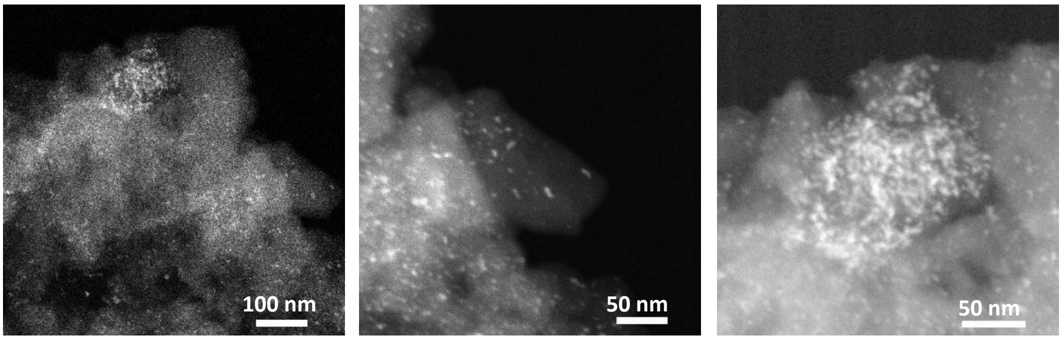

Figure S4 STEM images of **IrNPs@GNPs** at various magnifications showing the distribution and morphology of iridium nanoparticles on the graphene surface.

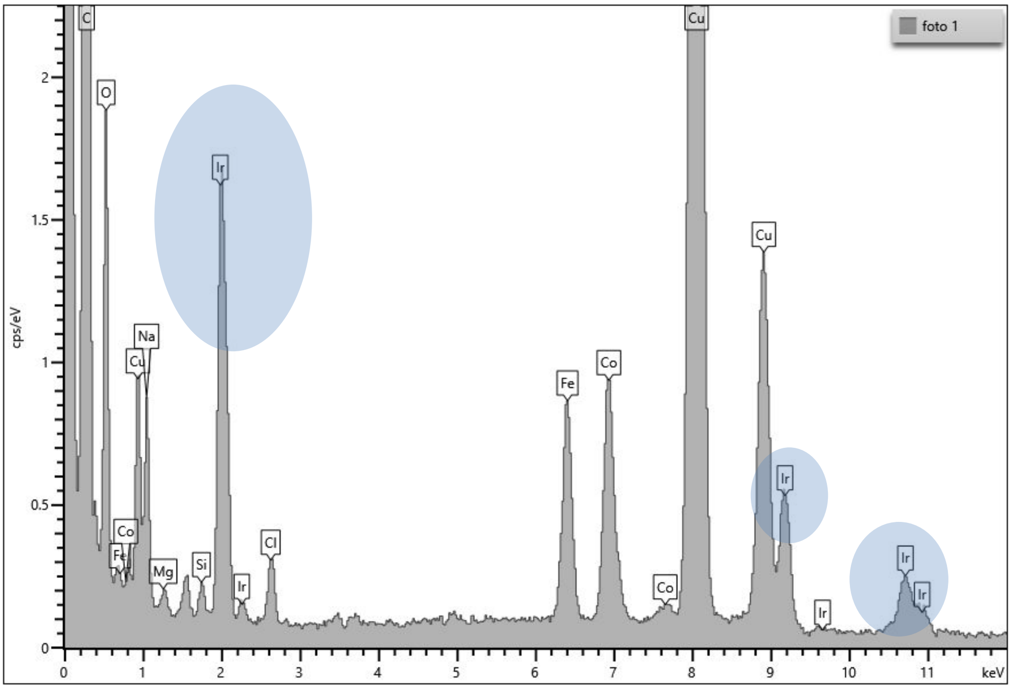

Figure S5 EDX spectrum of **IrNPs@GNPs** confirming the presence of iridium. The copper signal originates from the TEM grid used during analysis.

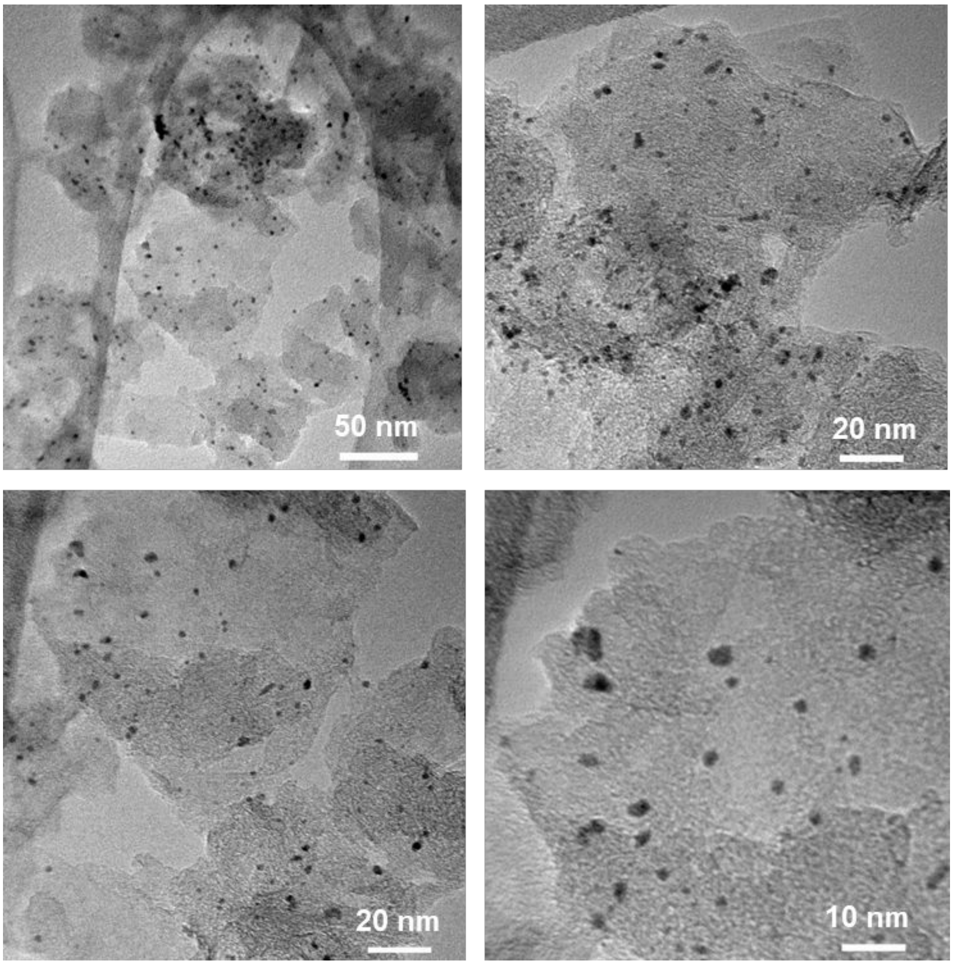

Figure S6 HRTEM images of spent **IrNPs@GNPs** at various magnifications (Images after 6 runs in ODH reaction of *p*-methoxybenzyl alcohol)

S3. X-ray photoelectron spectroscopy (XPS)

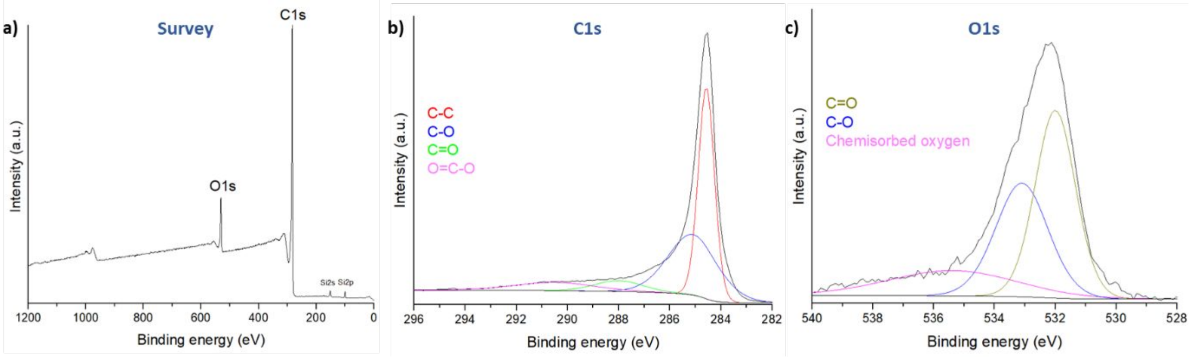

Figure S*7* XPS of **GNPs** used as support for IrNPs. (a) Survey spectrum of **GNPs**. (b) C1s band with deconvolution. (c) O1s band with deconvolution.

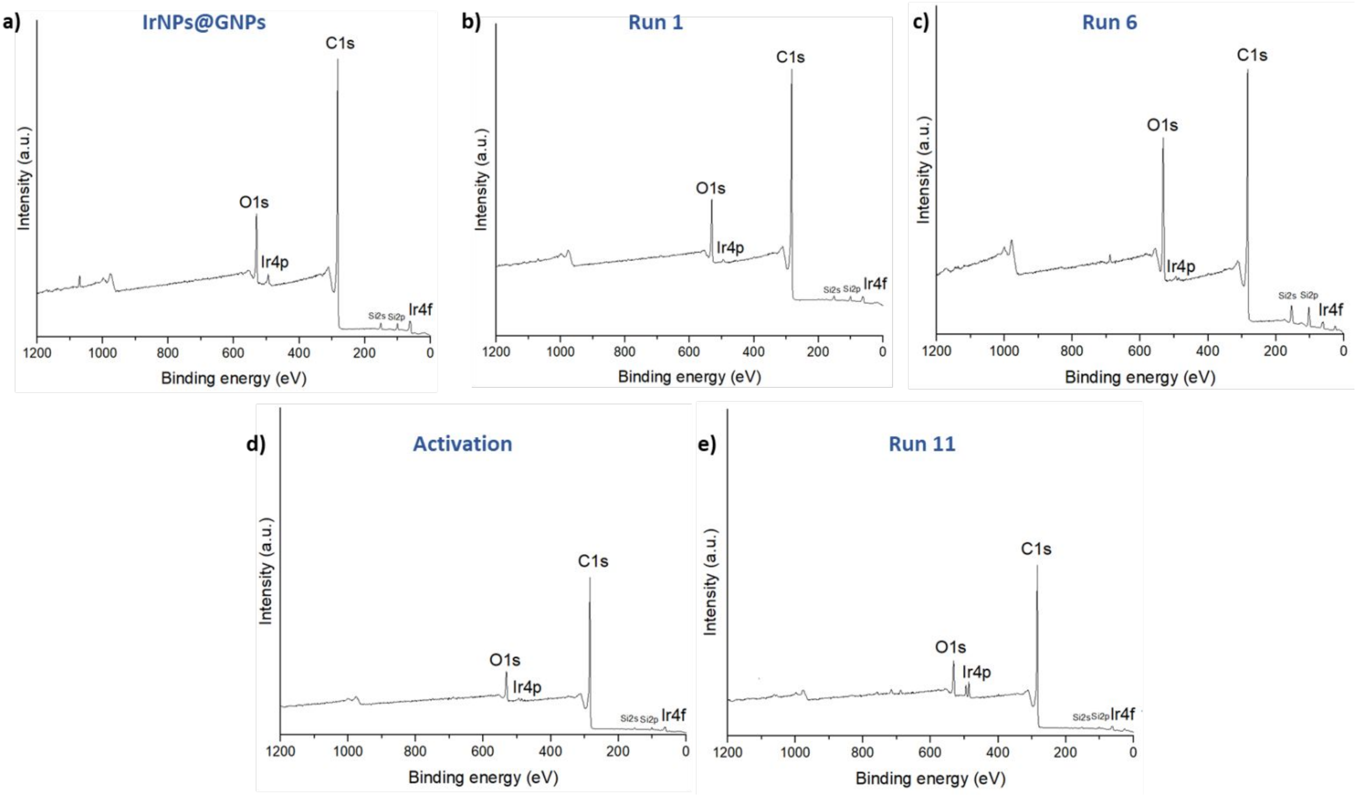

Figure S8 XPS survey spectra of **IrNPs@GNPs** as prepared (a), after run 1 (b), run 6 (c), activation (d) and run 11 (e).

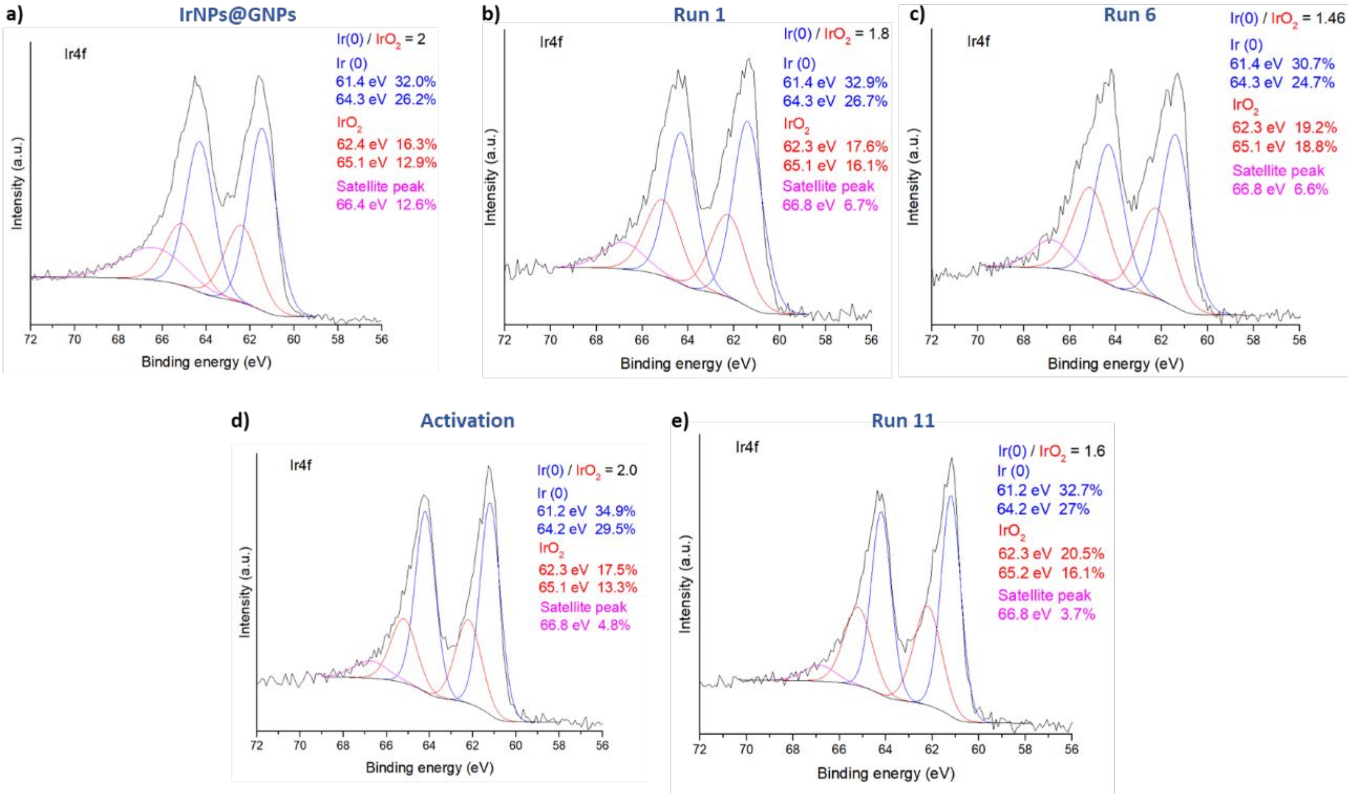

%	IrNPs@GNPs	Run 1	Run 6	Activation	Run 11
Ir(0)	66.7	64.3	59.4	66.7	61.5
IrO_2	33.3	35.7	40.6	33.3	38.5

Figure S9 High-resolution XPS spectra of Ir4f core-level peak of **IrNPs@GNPs** as prepared (a), after run 1 (b), run 6 (c), activation (d) and run 11 (e). Table: evolution of Ir(0) and IrO_2 during reusability experiments.

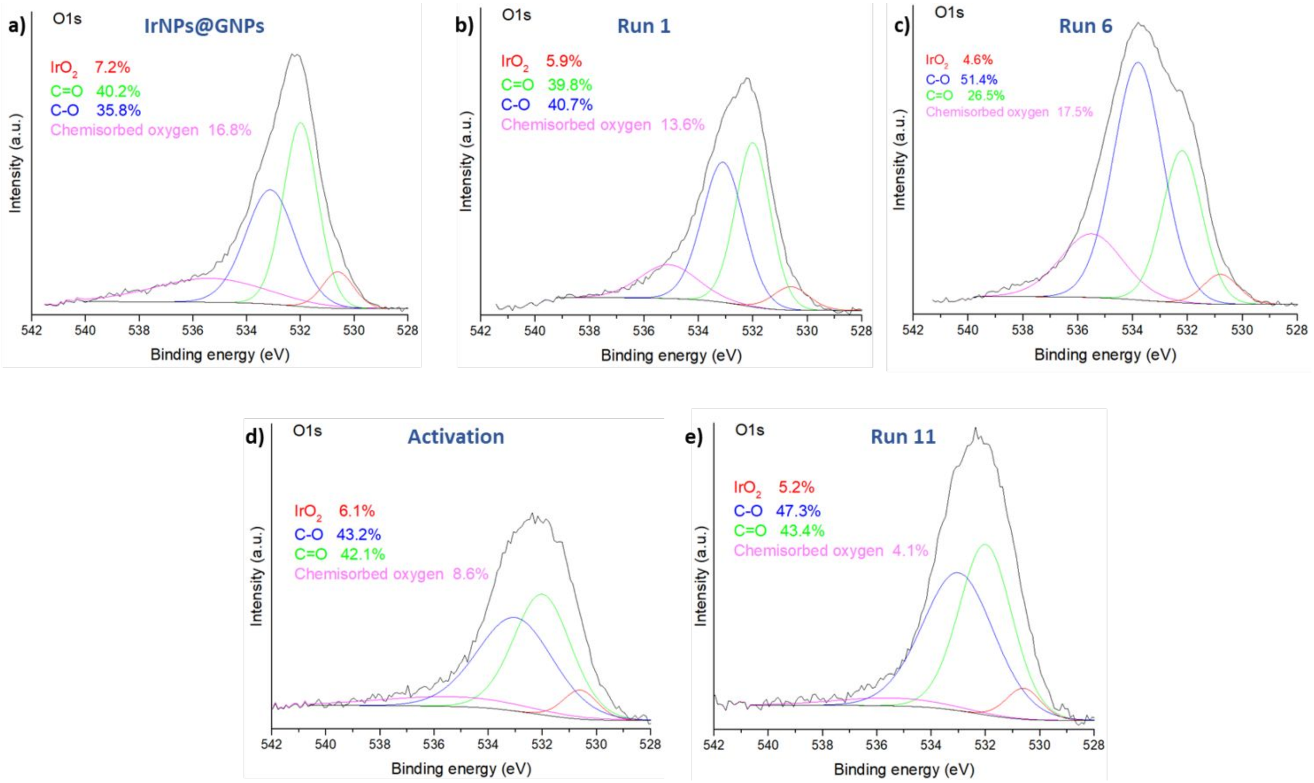

Figure S10 High-resolution XPS spectra of O1s core-level peak of **IrNPs@GNPs** as prepared (a), after run 1 (b), run 6 (c), activation (d) and run 11 (e).

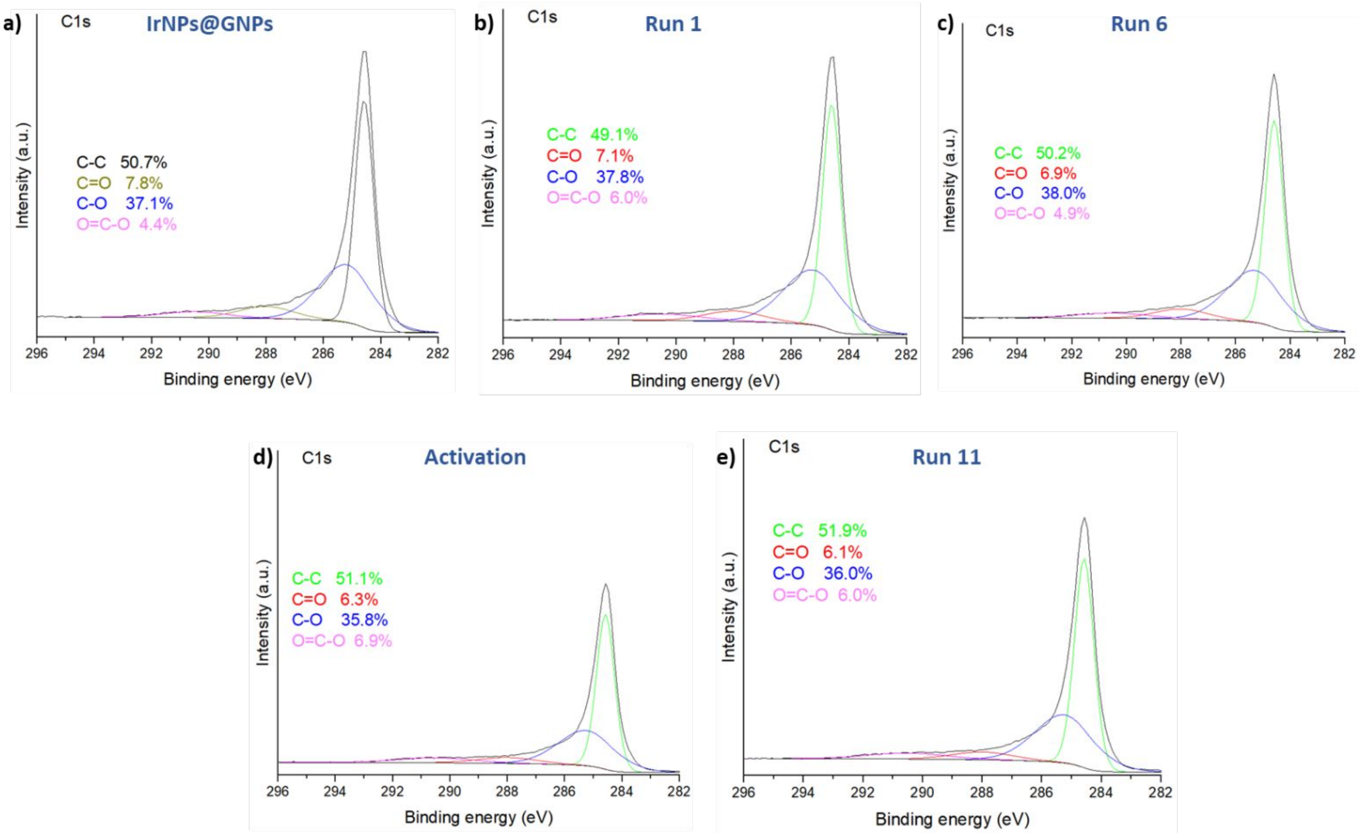

Figure S11 High-resolution XPS spectra of C1s core-level peak of **IrNPs@GNPs** as prepared (a), after run 1 (b), run 6 (c), activation (d) and run 11 (e).

S4. Raman spectroscopy

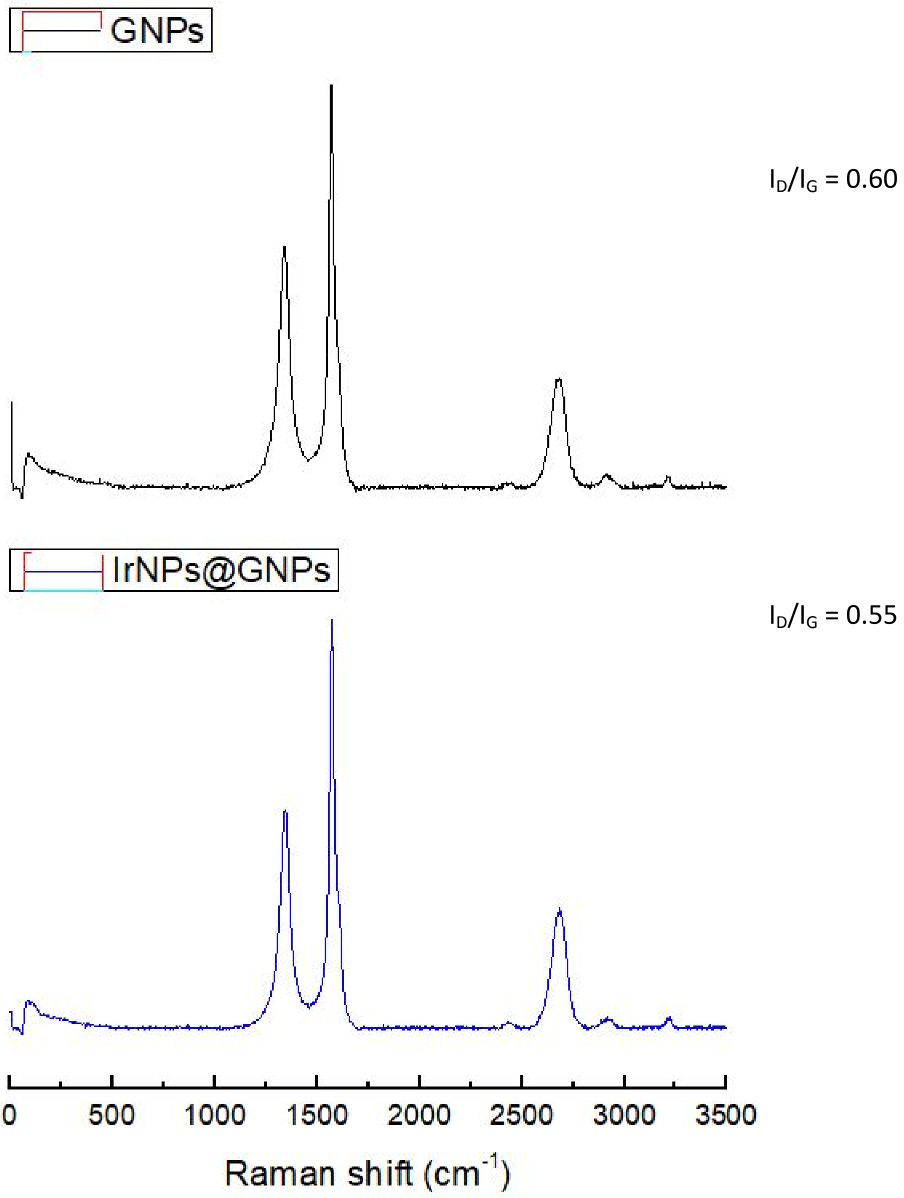

Figure S12 Comparative Raman spectra of **IrNPs@GNPs** and **GNPs**.

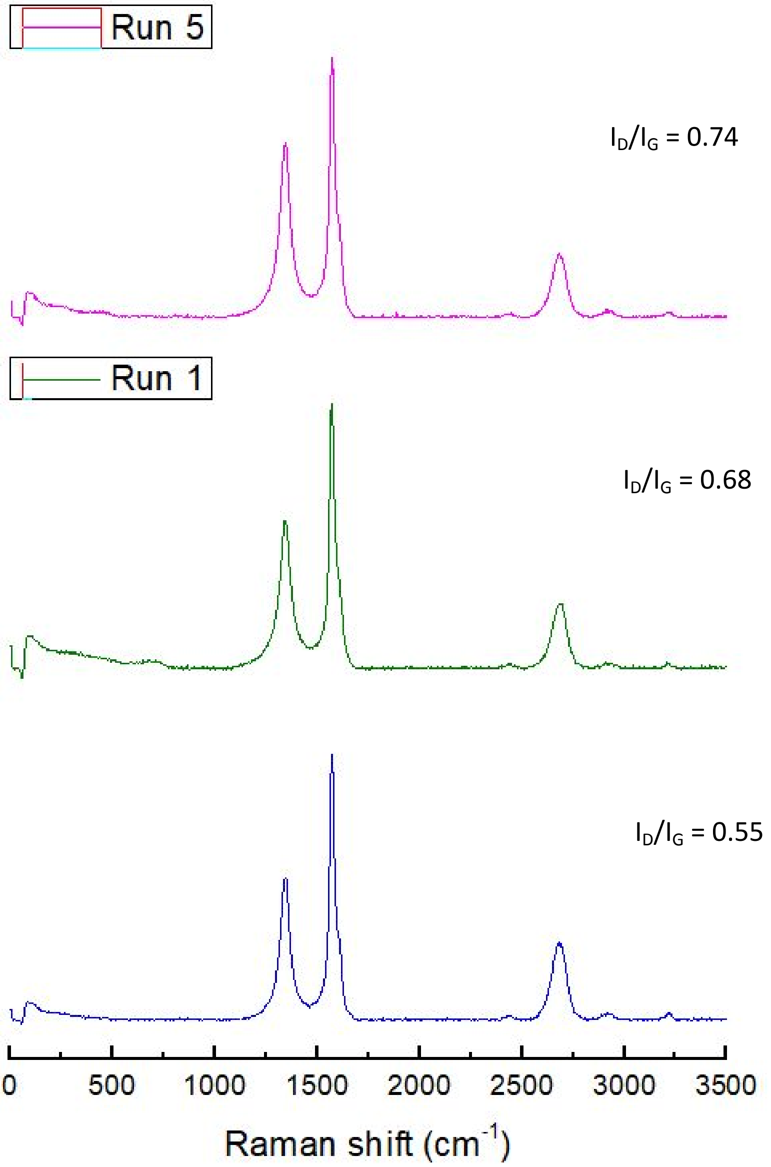

Figure S13 Comparative Raman spectra of **IrNPs@GNPs** after several runs in ODH reaction of *p*-methoxybenzyl alcohol.

S5. Synthesis and characterization of IrNPs@GNPs starting from $IrBr_3 \cdot 3H_2O$

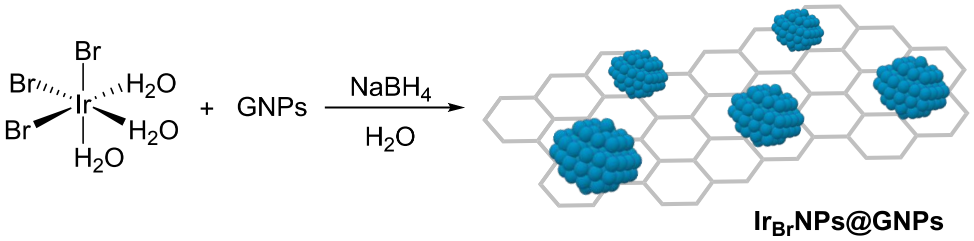

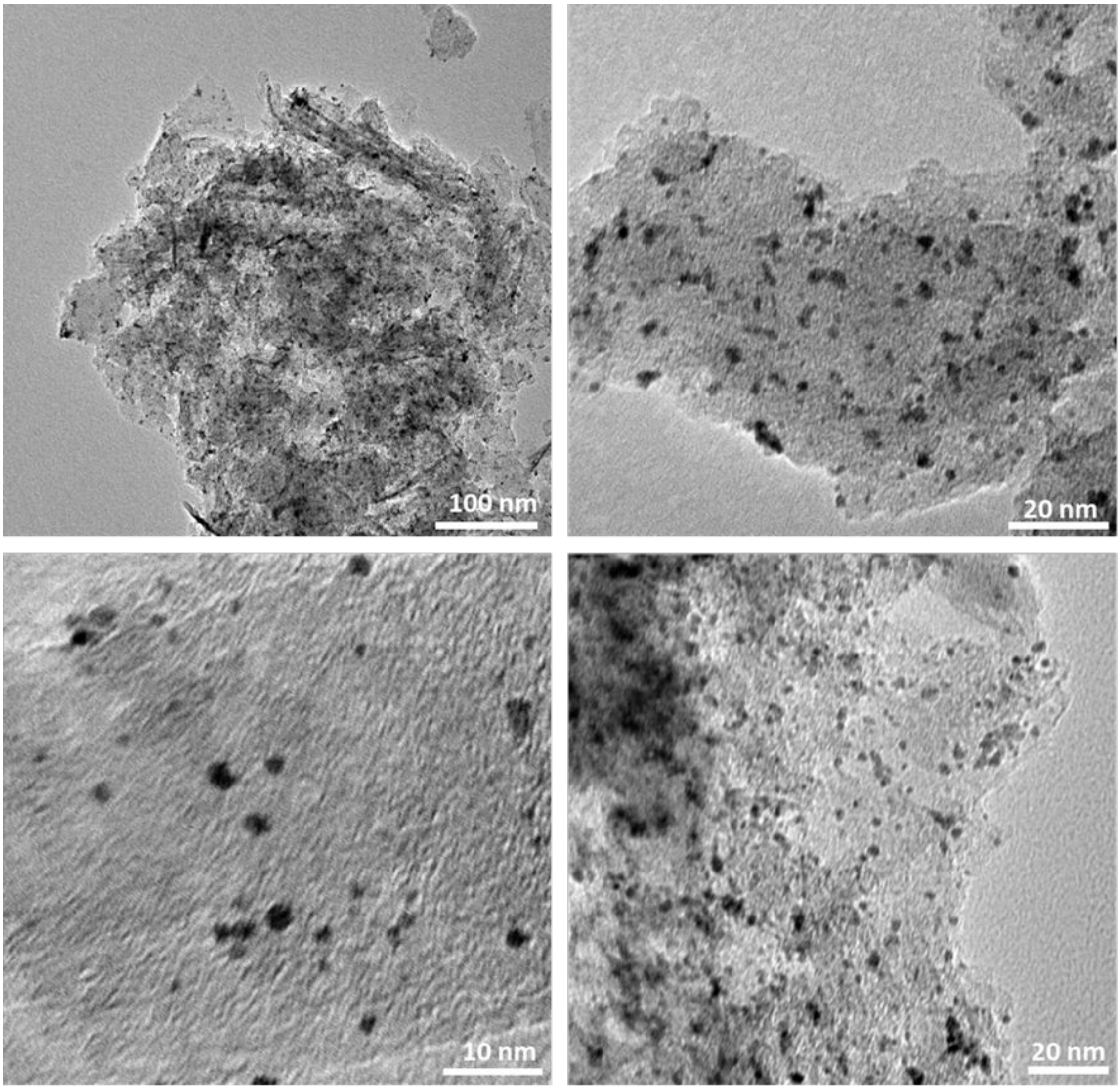

Figure S14 HRTEM images of **Ir_{Br}NPs@GNPs** at various magnifications.

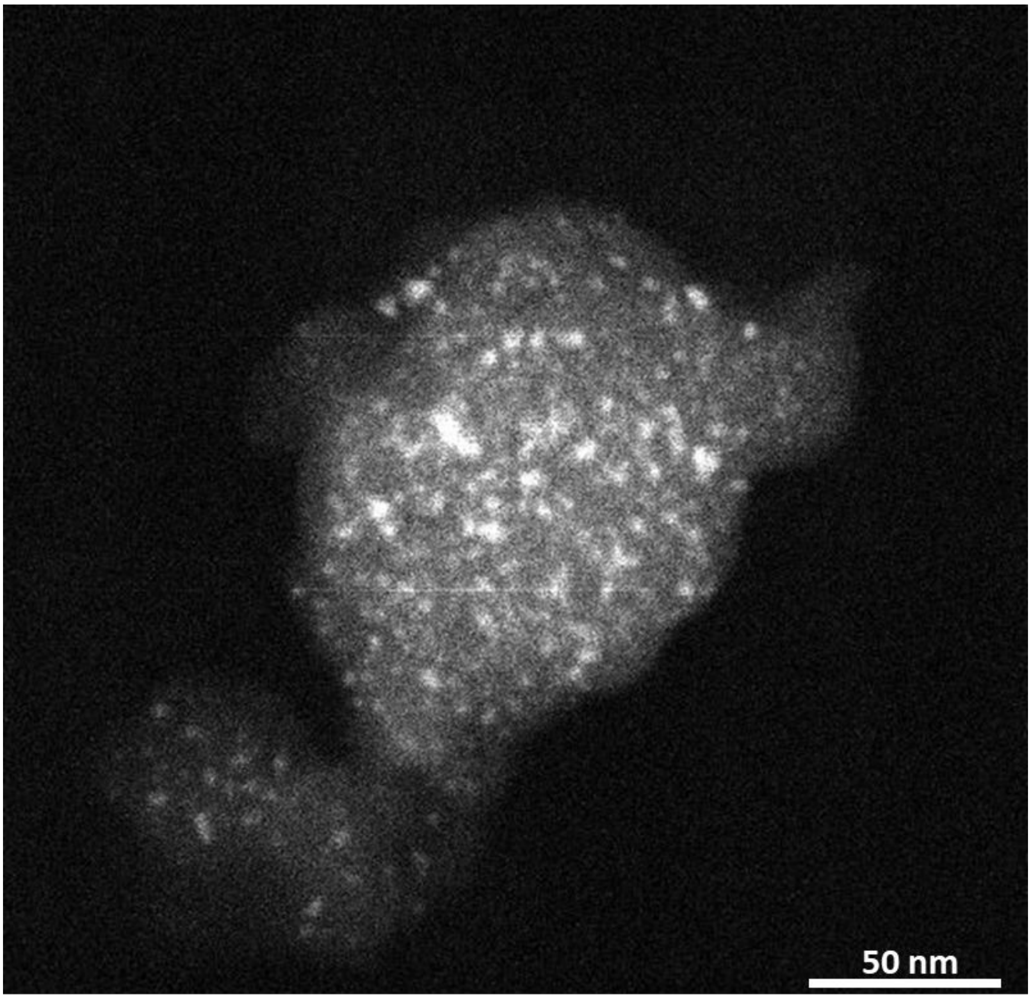

Figure S15 STEM images of **Ir_{Br}NPs@GNPs**

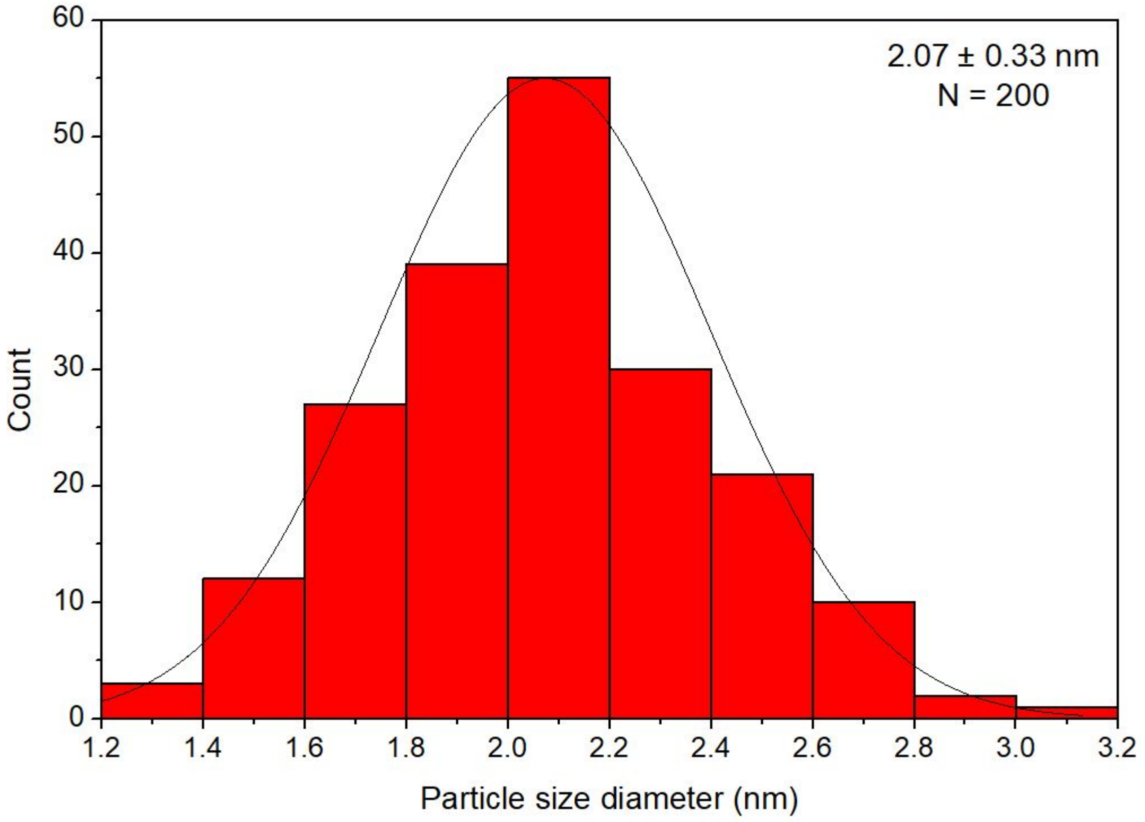

Figure S16 Size histogram of **Ir$_{Br}$NPs@GNPs**

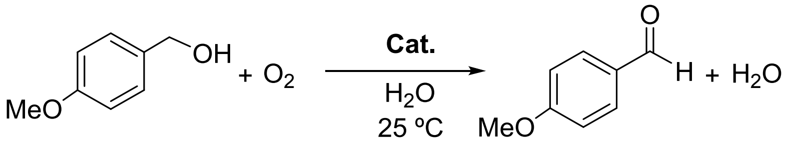

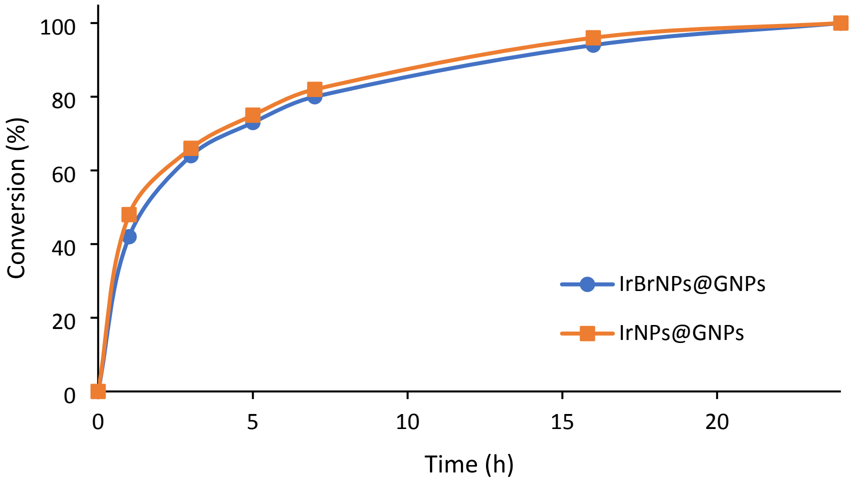

Figure S17 Comparison catalytic activity of **IrNPs@GNPs** vs **Ir$_{Br}$NPs@GNPs**

S6. Detection of H_2O formation

The ODH of *p*-methoxybenzyl alcohol was performed under the standard conditions but using deuterated toluene as solvent. A 1 mL aliquot was extracted, filtered and analyzed by ^1H NMR at initial time and after 8 hours. The formation of water was confirmed after comparing the ^1H NMR spectra of the aliquots extracted. The increment of water signal agrees with the conversion observed by GC/FID analysis using anisole as an external standard.

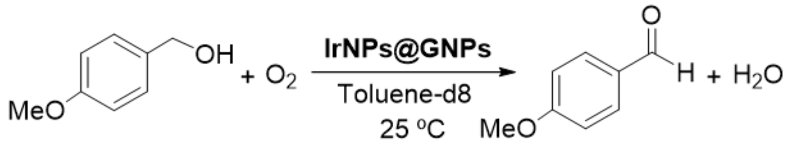

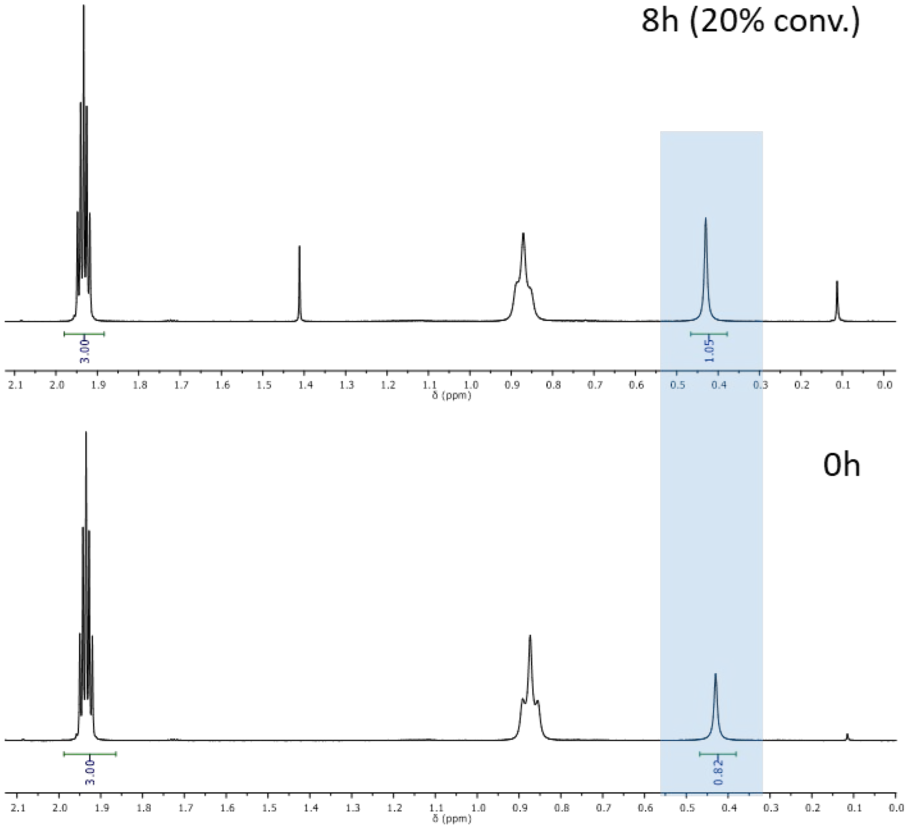

Figure S18 Experimental determination of water formation. Water signal increment highlighted in blue.

S7. Estimation of surface atoms in IrNPs@GNPs nanoparticles

The estimation of surface atoms in **IrNPs@GNPs** was performed assuming a spherical nanoparticle morphology, with the radius (*r*) determined from a size histogram obtained by analyzing at least 200 particles in STEM images. First, the total number of Ir atoms was calculated using the volume of a sphere with radius *r*, based on the know density of iridium. Then, a second calculation was performed using a sphere of radius r-2r_c, where r_c is the covalent radius of iridium, to approximate the number of atoms within the nanoparticle's interior. The difference between these two values corresponds to the number of surface atoms, which we assume to be catalytically active. Atoms in direct contact with the graphene surface were not excluded from this estimation. The ratio of surface atoms to total atoms is referred to as the dispersion, dispersion degree or dispersity. [1]

Table S1: Estimation of surface atoms

**Data**		
Iridium density (g/cm^3)	22.42	
Iridium atomic mass (g/mo)	192.22	
Na Avogadro number	6.023 x 10^{23}	
Iridium covalent radi (nm)	0.138	
Diameter of IrNPs from STEM (nm)	2.12 ± 0.54	

**Total atoms**		
Sphere volume	V_{sphere}	4.8491E-21
Mass of Iridium	m_{Ir}	1.08716E-19
Moles of Iridium	n_{Ir}	5.65581E-22
Number of Iridium atoms	$\#_{Ir\ atoms}$	341
**Atoms subsurface**		
Sphere volume	V_{sphere}	1.9423E-21
Mass of Iridium	m_{Ir}	4.3546E-20
Moles of Iridium	n_{Ir}	2.2654E-22
Number of Iridium atoms	$\#_{Ir\ atoms}$	136

**Ir surface atoms**	204
**Dispersion**	59.9%

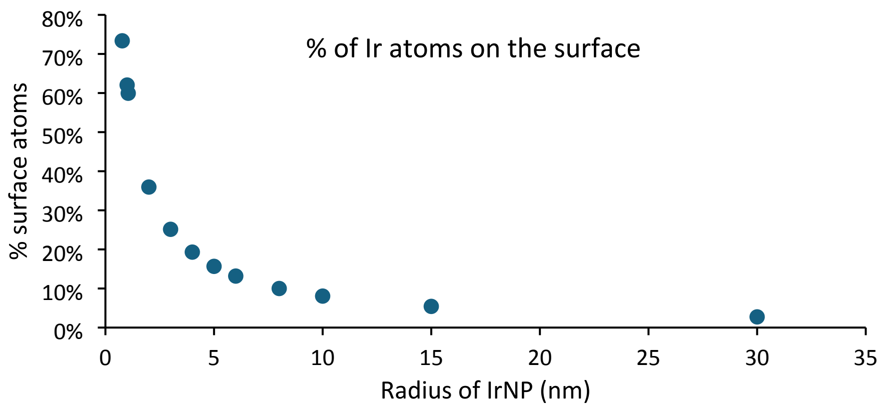

Figure S19 Variation of Iridium surface atoms as a function of particle size.

S8. Kinetic studies

S8.1 Order in catalyst

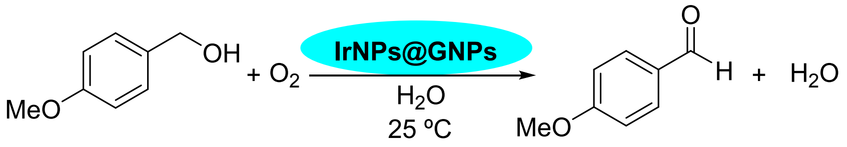

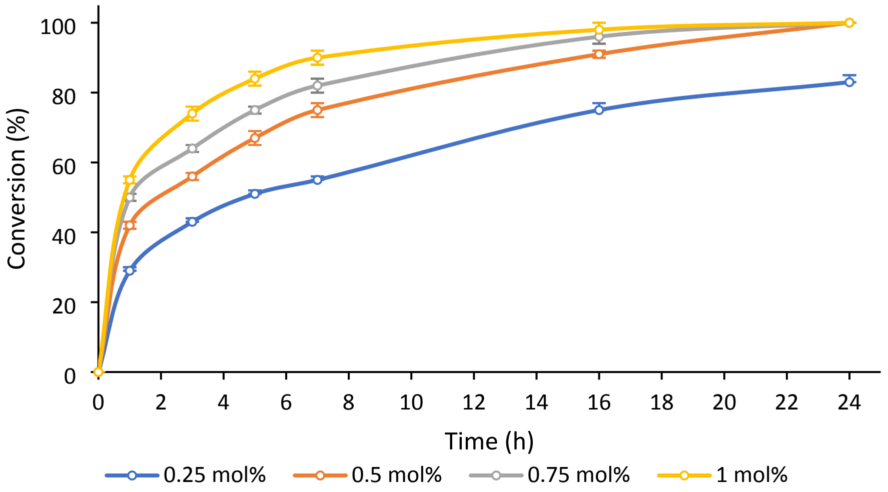

Figure S20 Reaction progress profiles in the ODH of *p*-methoxybenzyl alcohol with various catalyst loadings. Reaction conditions: *p*-methoxybenzyl alcohol (0.5 mmol), catalyst loading (x mol% based on the total amount of Ir obtained by ICP-MS), room temperature, solvent (5 mL), aerobic conditions.

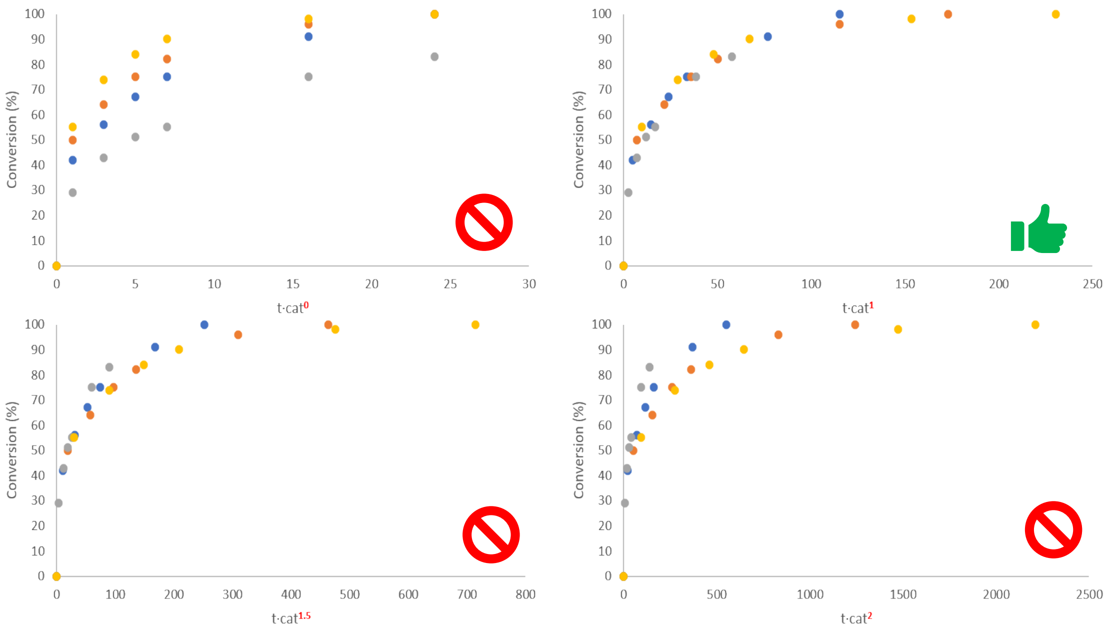

Figure S21 Evaluation of order in catalyst using variable time normalization analysis (VTNA).[2–4] The order in catalysts corresponds to the plot where more data points are overlayed according to VTNA, which is 1 in this case. Data: yellow circles (1 mol%), orange circles (0.75 mol%), blue circles (0.5 mol%) and grey circles (0.25 mol%).

S8.2 Order in substrate

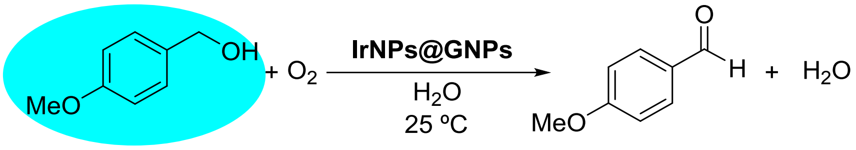

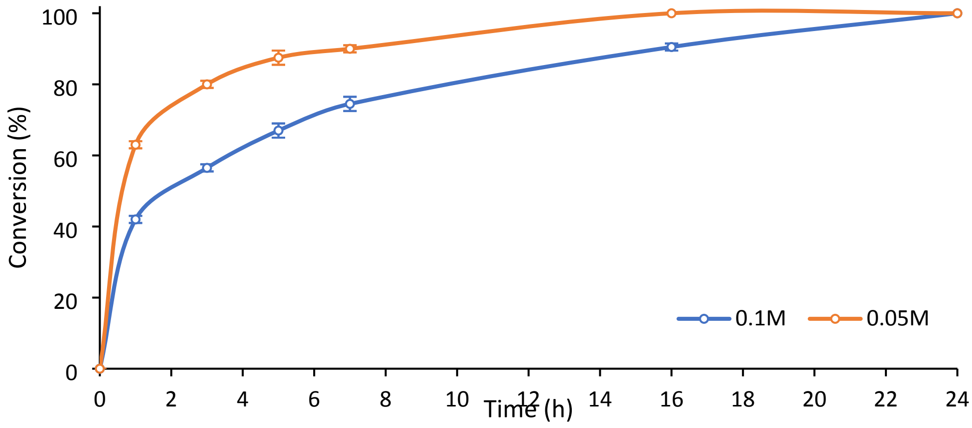

Figure S22 Reaction progress profiles in the ODH of *p*-methoxybenzyl alcohol at different initial concentration of substrate.

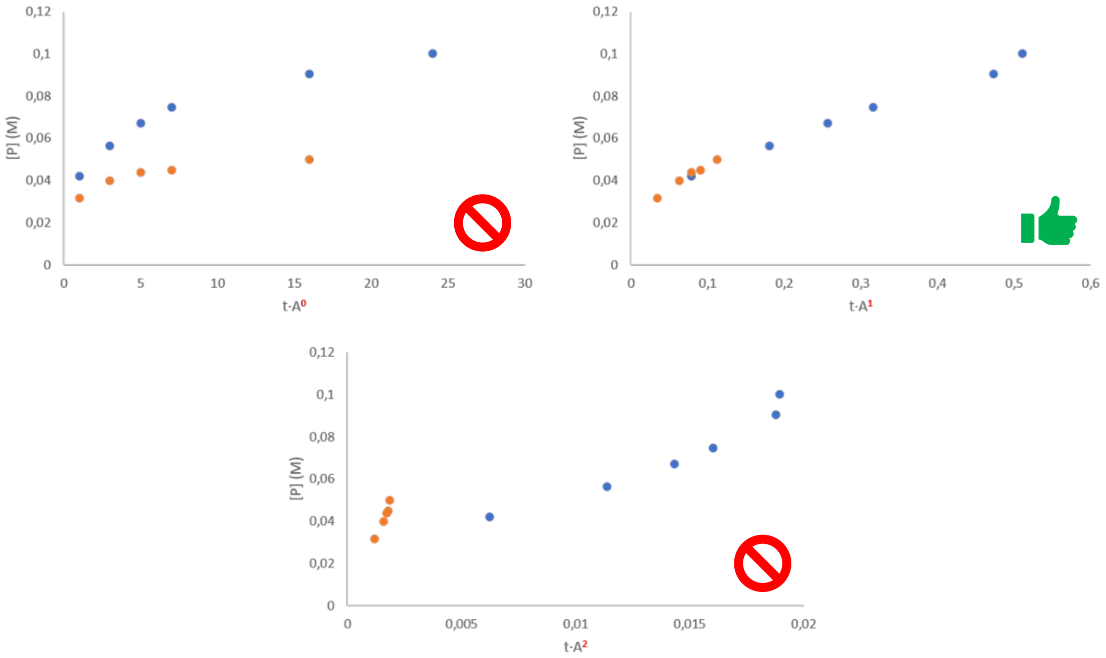

Figure S23 Evaluation of order in substrate using variable time normalization analysis (VTNA).[2–4] The order in substrate corresponds to the plot where more data points are overlayed according to VTNA, which is 1 in this case. Data: orange circles ($[Substrate]_o$ = 0.05 M) and blue circles ($[Substrate]_o$ = 0.1 M).

S8.3 Thermodynamic data

A graphical method developed by Rivero-Crespo et al. was used to estimate the enthalpy ($\Delta H^{\neq}$) and entropy ($\Delta S^{\neq}$) transition state, their associated error and the activation energy (E_a).[5] The method is based on the Eyring-Polanyi equation using a normalized time-scale function. From an experimental approach, the methodology requires reaction monitoring profiles at different temperatures. The values of thermodynamic data are found in an iterative way until the kinetic profiles graphically overlay.

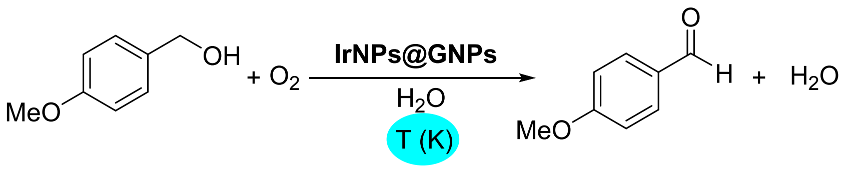

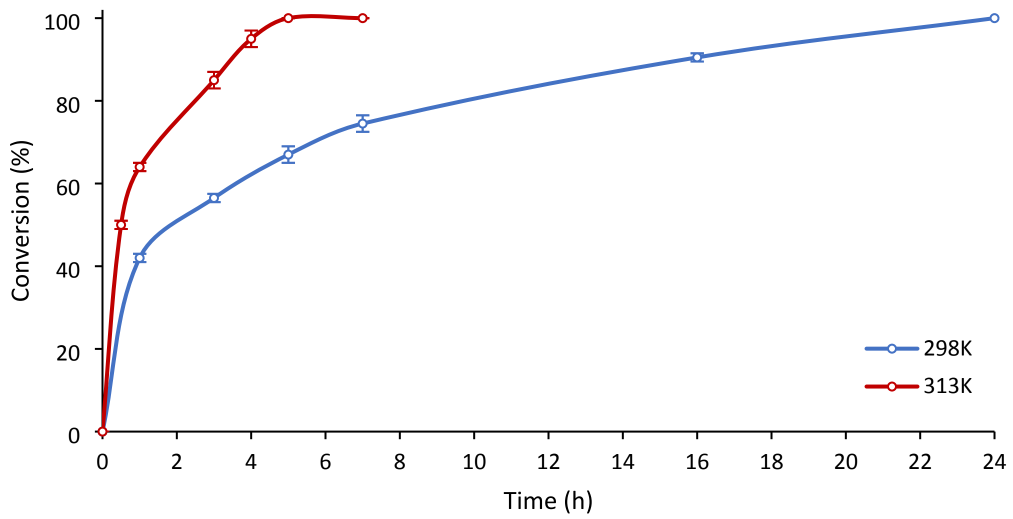

Figure S24 Reaction progress profiles in the ODH of *p*-methoxybenzyl alcohol at different temperatures.

Enthalpy transition state (ΔH‡)

$$f(t, T, \Delta H\ trans.) = t \cdot T \cdot e^{-\Delta H\ trans./_{RT}}$$

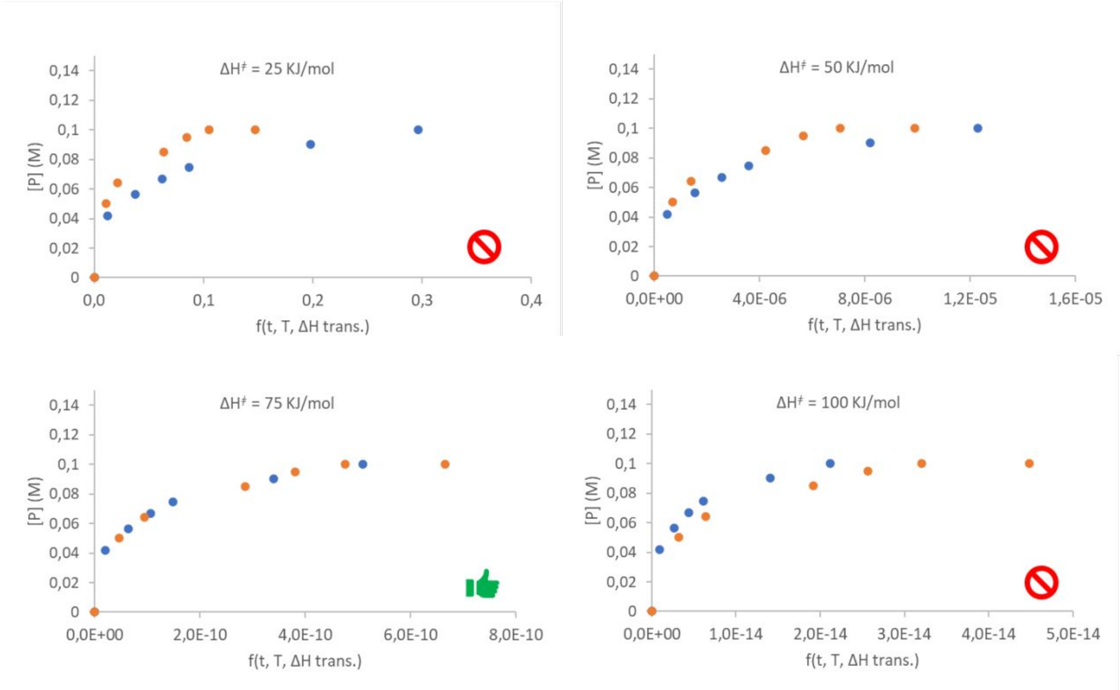

Figure S25 Graphical method used to estimate the transition state enthalpy (**ΔH‡**). The **ΔH‡** value corresponds to the plot where more data points are overlayed, 75 KJ/mol in this case. Data: Orange circles at 313 K and blue circles at 298 K.

Entropy transition state (ΔS‡)

$$g(t, T, \Delta S\ trans.) = \left(t \cdot \frac{K_B \cdot T}{h}\right)^{\frac{T}{T_m}} \cdot e^{\frac{\Delta S\ trans. \cdot T}{R \cdot T_m}}$$

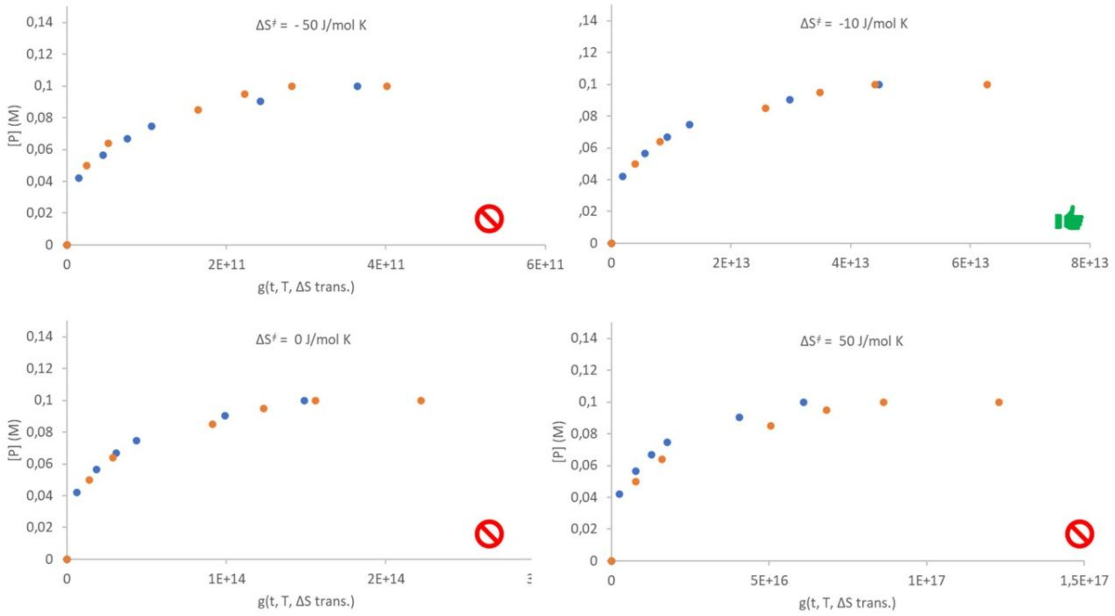

Figure S26 Graphical method used to estimate the transition state entropy (**ΔS‡**). The **ΔS‡** value corresponds to the plot where more data points are overlayed, -10 J/molK in this case. Data: Orange circles at 313 K and blue circles at 298 K.

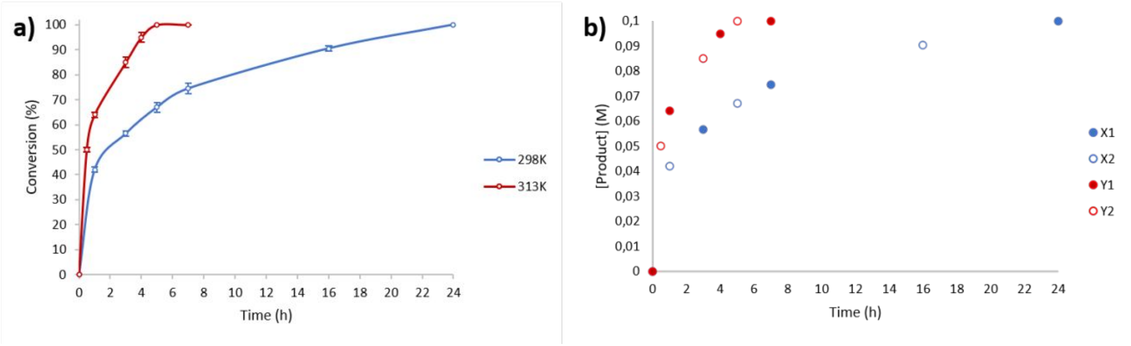

c)

**Data set**	**ΔH‡ (KJ/mol)**	**ΔS‡ (KJ/mol)**
X-Y	75	-10
X1-Y1	75.5	-10.5
X1-Y2	76	-9.2
X2-Y1	76.5	-10
X2-Y2	77	-10
**Average**	75.8 ± 0.8	-10.0 ± 0.4

Figure S27 Associated error: a) Time-conversion profiles at various temperatures. b) Data split in four subsets. c) Data used for the calculation of the thermodynamic variables and their associated error.

S8.4 Artificial intelligence (AI) mechanism proposal

The input data for the machine learning algorithm crafted by Burés and Llarrosa consist in monitoring profiles (Figure S20).[6] We utilized the pre-trained model furnished by the authors, specifically the comprehensive model **M1_20_model_S_noXS_01to5.n5**, which incorporates substrate concentration vs time experiments for catalyst loadings of 0.25, 0.75, and 1 mol%. The code was run with *Python 3.9*. To satisfy the requirement of the model for uniform time points, 7 time points spanning from 0 to 24 hours (both included) were selected for the analysis.

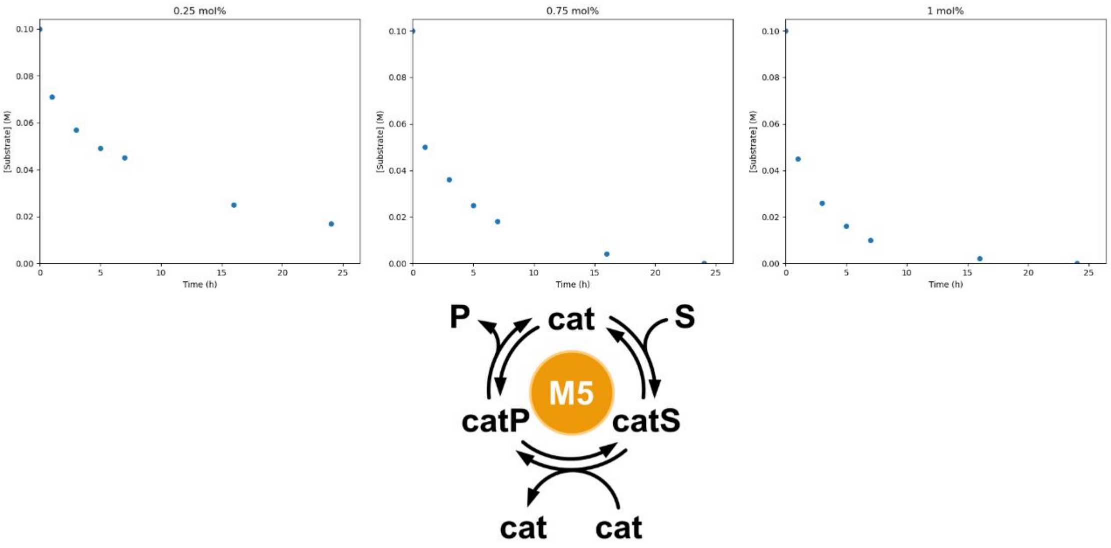

Figure S28 Kinetic data used for machine learning algorithm and mechanism prediction for three kinetic data.

S9. Gas chromatography spectra of organic compounds

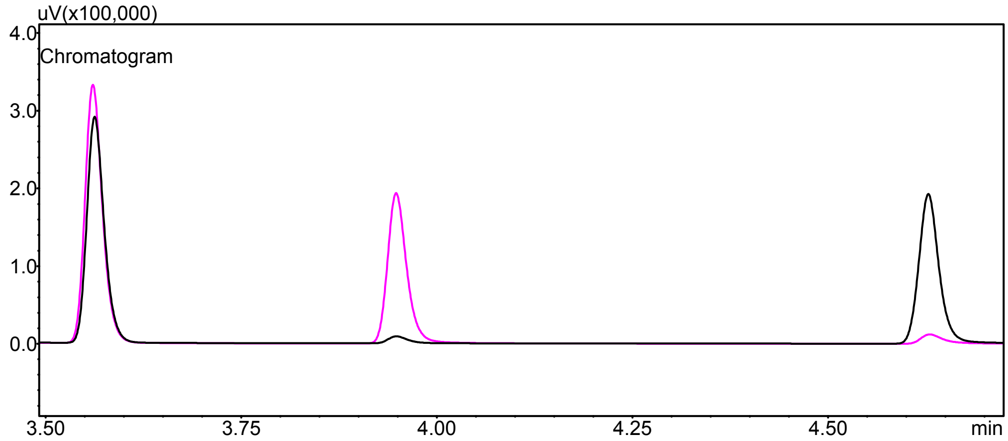

Figure S29 Representative reaction progress by GC/FID in the ODH of benzylalcohol (r.t. = 4.6 min) to benzaldehyde (r.t. = 3.9 min) in the presence of 0.5 mmol of anisole as an external standard (r.t. = 3.6 min). Black line: 0h; pink line: 24h. Temperature program: 1) 80 °C, 10 °C/min until 150 °C, 1 min. 2) 150 °C, 20 °C/min until 300 °C, 2 min. Total time: 17.5 min.

S10. ^{1}H NMR spectra of crude reaction products

In a generic catalytic reaction, 0.5 mmol of substrate and the required amount of catalyst are dispersed in 5 mL of deionized water and stirred at room temperature for 24 hours. After the extraction of organic products with CH2Cl2 (3 x 5 mL), the reaction crude is analyzed by 1H NMR spectroscopy. Conversion and yield are quantified after adding a known amount of 1,3,5-trimethoxybenzene (TMB) or sodium formate (0.25 mmol of TMB or 0.5 mmol of sodium formate) as an external standard.

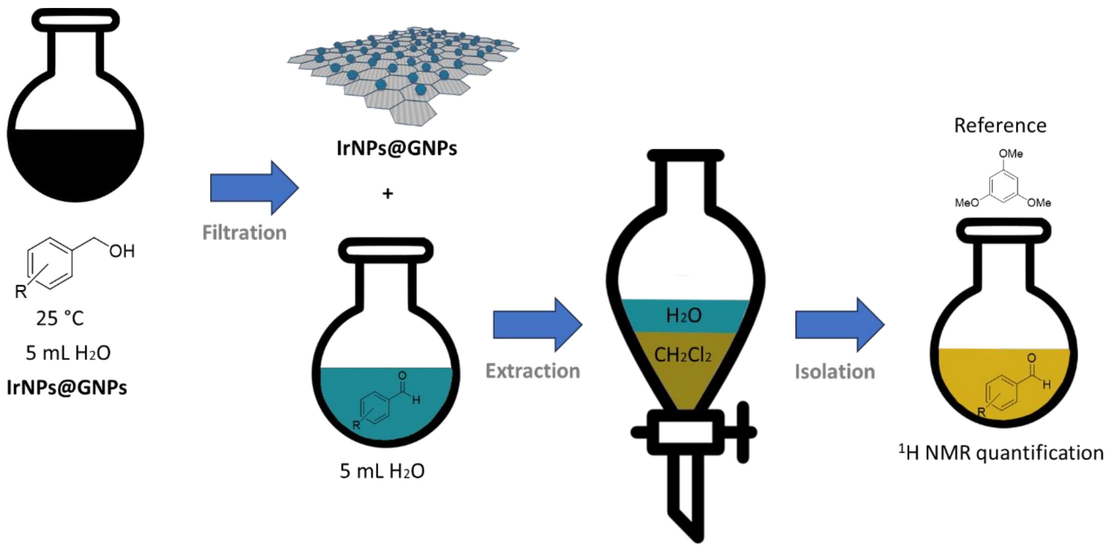

Figure S30 Experimental set-up for ^{1}H NMR quantification using 1,3,5-trimethoxybenze as an external standard.

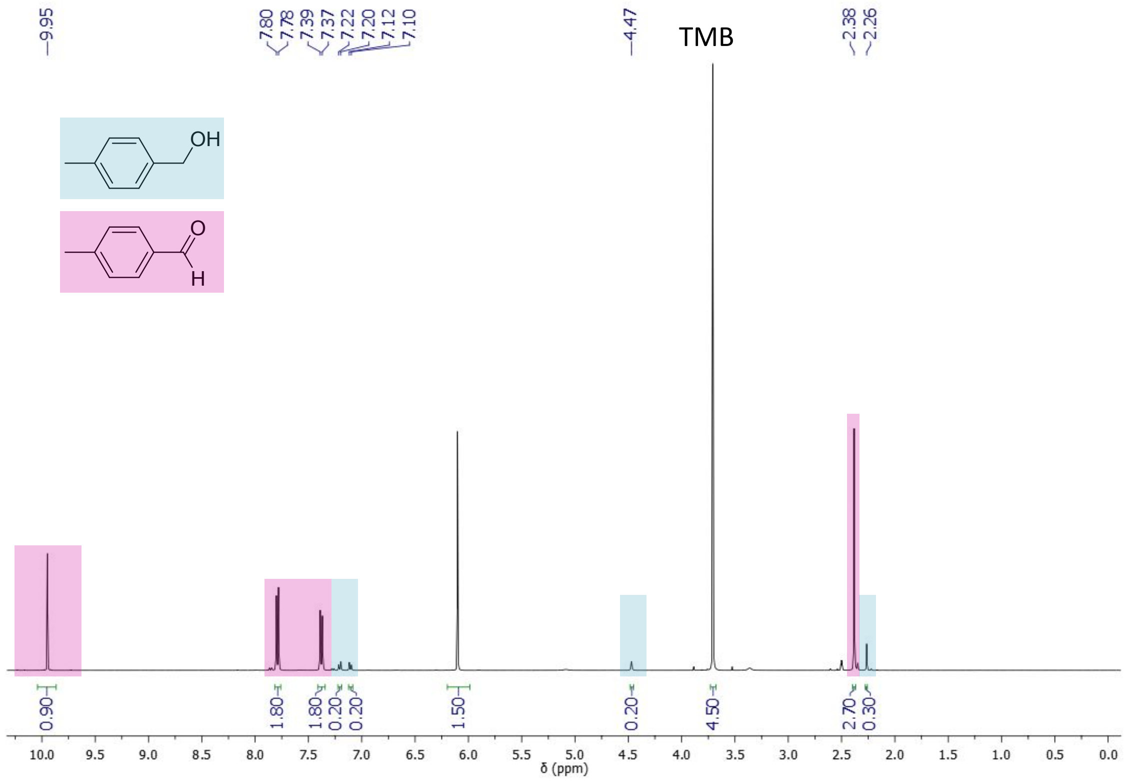

Figure S31 ^{1}H NMR spectrum of *p*-methylbenzaldehyde (Table 2, entry 1) in DMSO in the presence of 0.25 mmol of 1,3,5-trimethoxybenze as an external standard (6.1 ppm and 3.75 ppm).

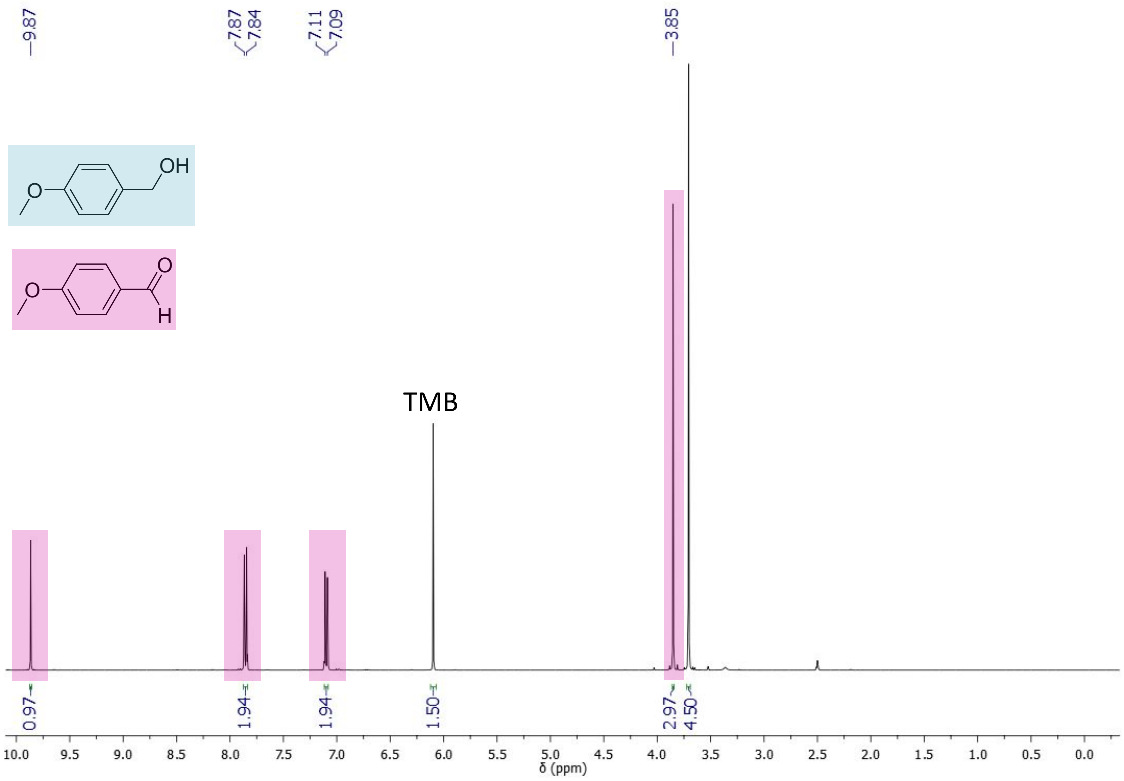

Figure S32 ^{1}H NMR spectrum of *p*-methoxylbenzaldehyde (Table 2, entry 2) in DMSO in the presence of 0.25 mmol of 1,3,5-trimethoxybenze as an external standard (6.1 ppm and 3.75 ppm).

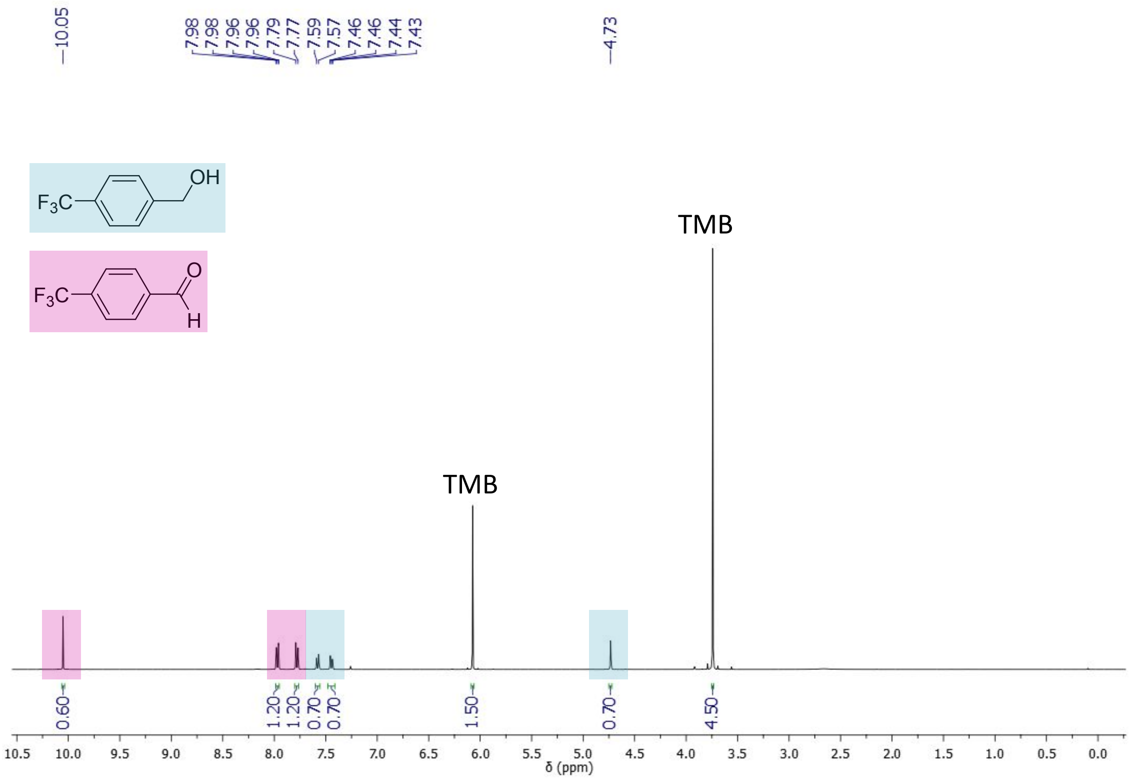

Figure S33 ^{1}H NMR spectrum of *p*-trifluoromethylbenzaldehyde (Table 2, entry 3) in $CDCl_3$ in the presence of 0.25 mmol of 1,3,5-trimethoxybenze as an external standard (6.1 ppm and 3.75 ppm).

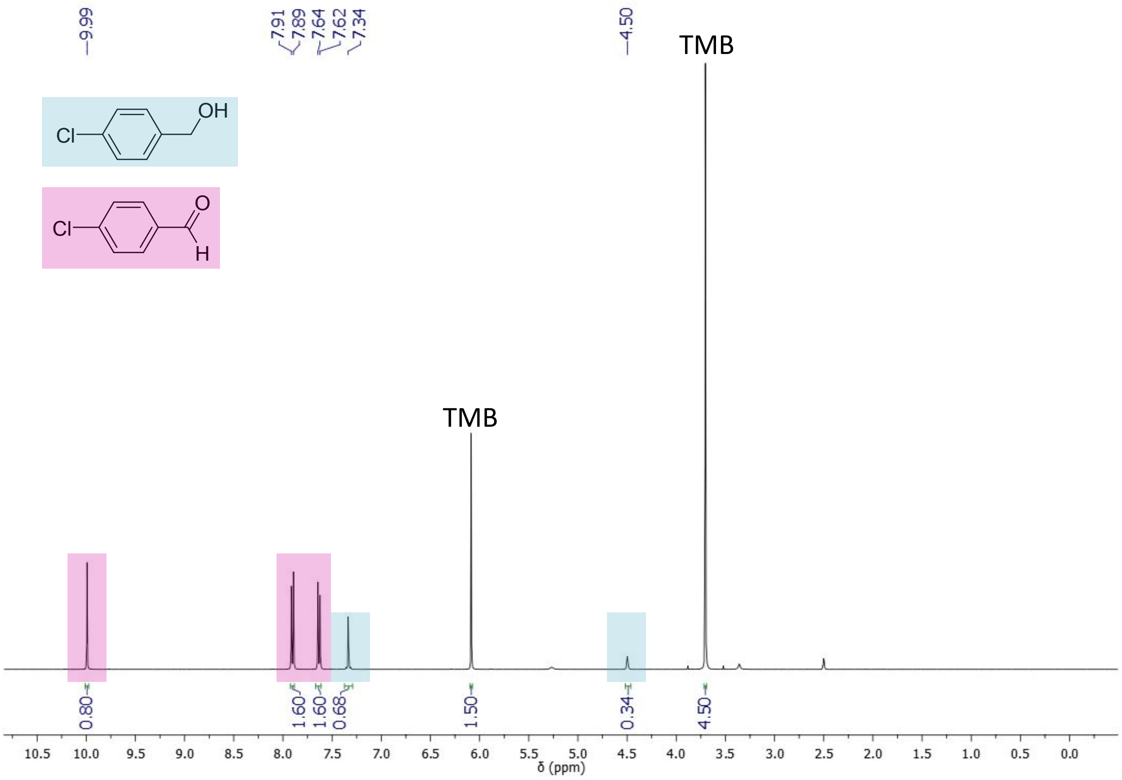

Figure S34 ^{1}H NMR spectrum of *p*-chlorobenzaldehyde (Table 2, entry 4) in DMSO in the presence of 0.25 mmol of 1,3,5-trimethoxybenze as an external standard (6.1 ppm and 3.75 ppm).

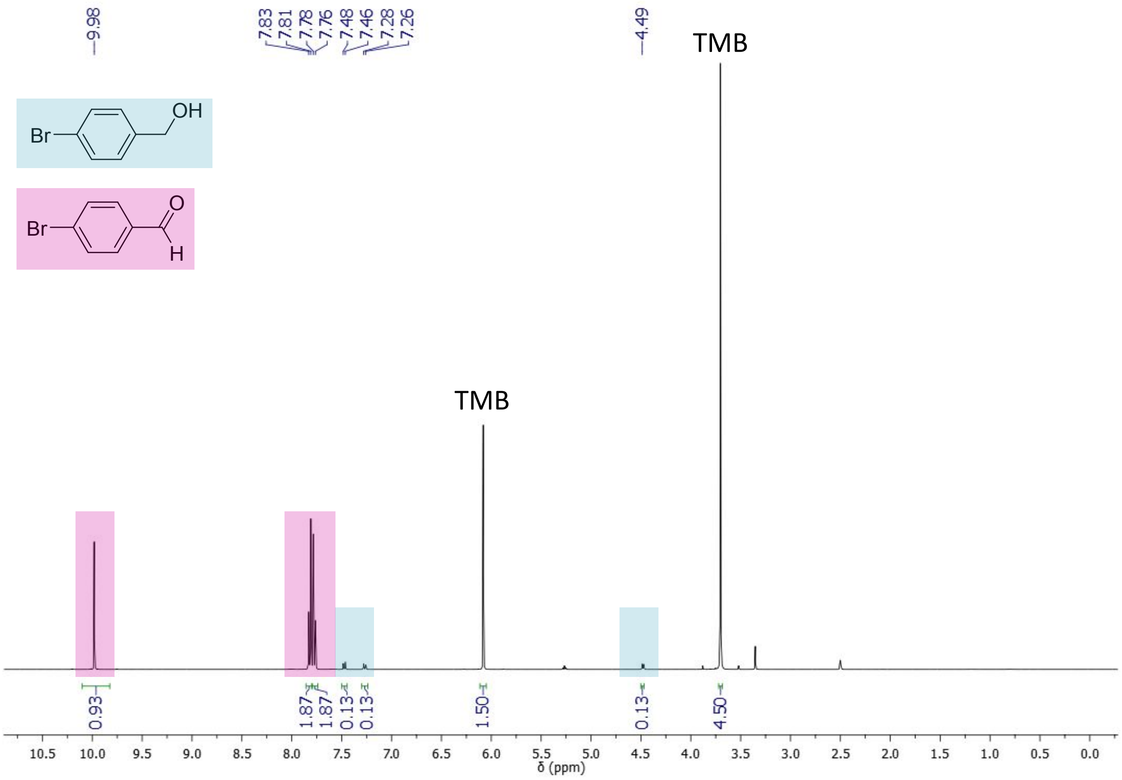

Figure S35 ^{1}H NMR spectrum of *p*-bromobenzaldehyde (Table 2, entry 6) in DMSO in the presence of 0.25 mmol of 1,3,5-trimethoxybenze as an external standard (6.1 ppm and 3.75 ppm).

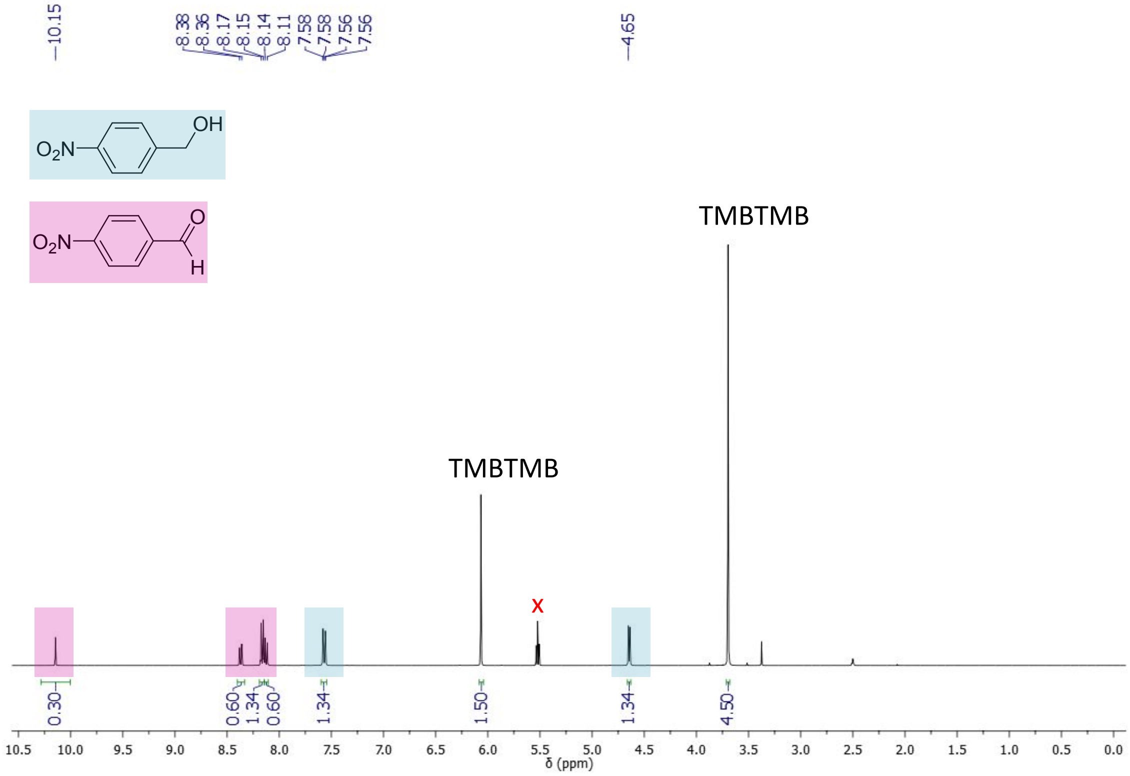

Figure S36 ^{1}H NMR spectrum of *p*-nitrobenzaldehyde (Table 2, entry 7) in DMSO in the presence of 0.25 mmol of 1,3,5-trimethoxybenze as an external standard (6.1 ppm and 3.75 ppm).

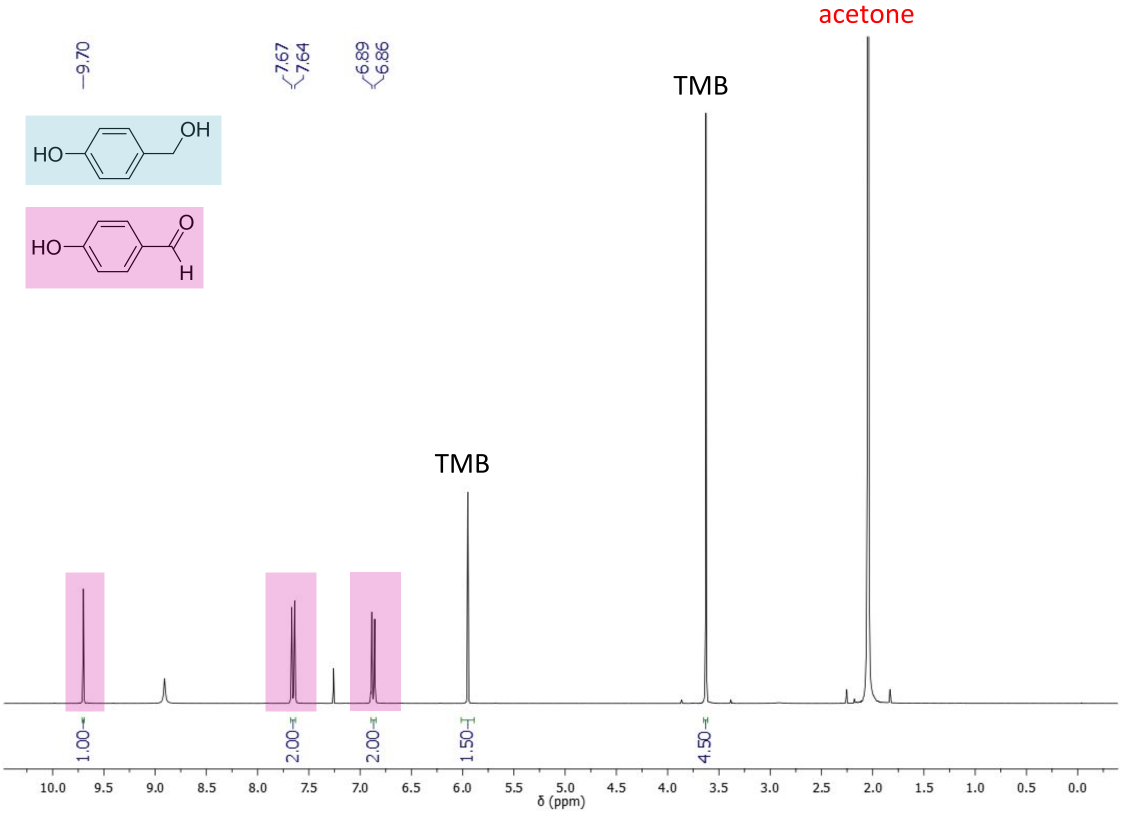

Figure S37 ^{1}H NMR spectrum of *p*-hydroxybenzyl alcohol (Table 2, entry 8) in $CDCl_3$ in the presence of 0.25 mmol of 1,3,5-trimethoxybenze as an external standard (6.1 ppm and 3.75 ppm).

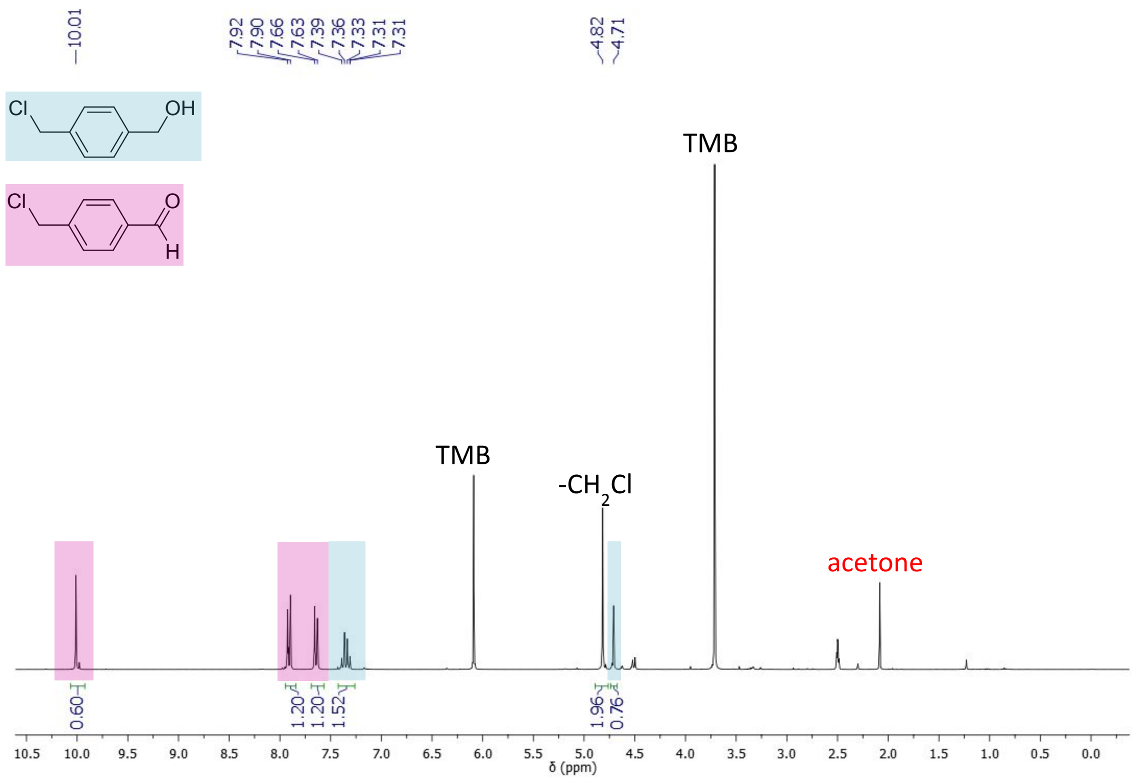

Figure S38 ^{1}H NMR spectrum of *p*-chloromethylbenzaldehyde (Table 2, entry 9) in DMSO in the presence of 0.25 mmol of 1,3,5-trimethoxybenze as an external standard (6.1 ppm and 3.75 ppm).

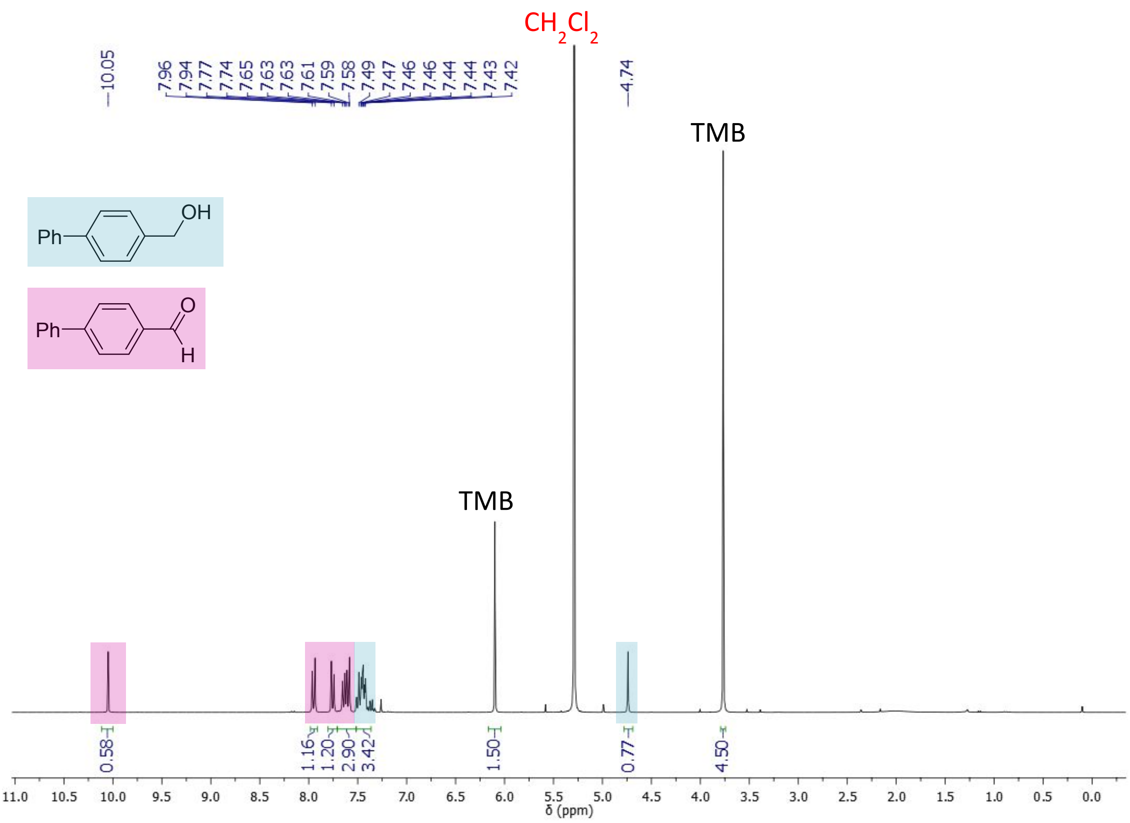

Figure S39 ^{1}H NMR (in $CDCl_3$) spectrum of biphenyl-4-methanol (Table 2, entry 10) in the presence of 0.25 mmol of 1,3,5-trimethoxybenze as an external standard (6.1 ppm and 3.75 ppm).

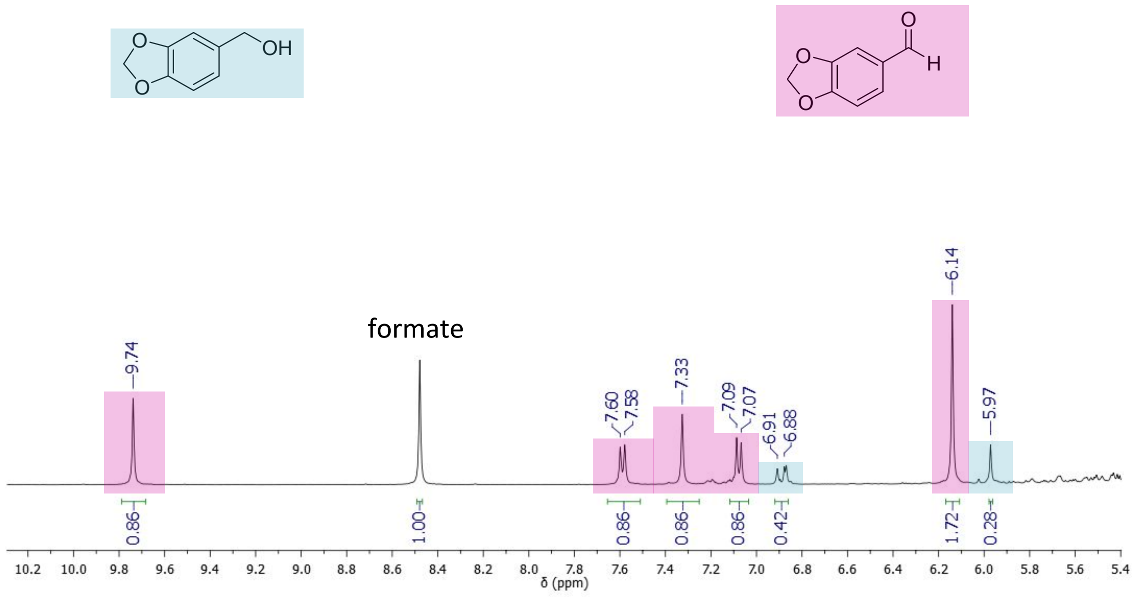

Figure S40 ^{1}H NMR (in D_2O) spectrum of piperonal (Table 2, entry 11) in the presence of 0.5 mmol of sodium formate as an external standard (8.5 ppm).

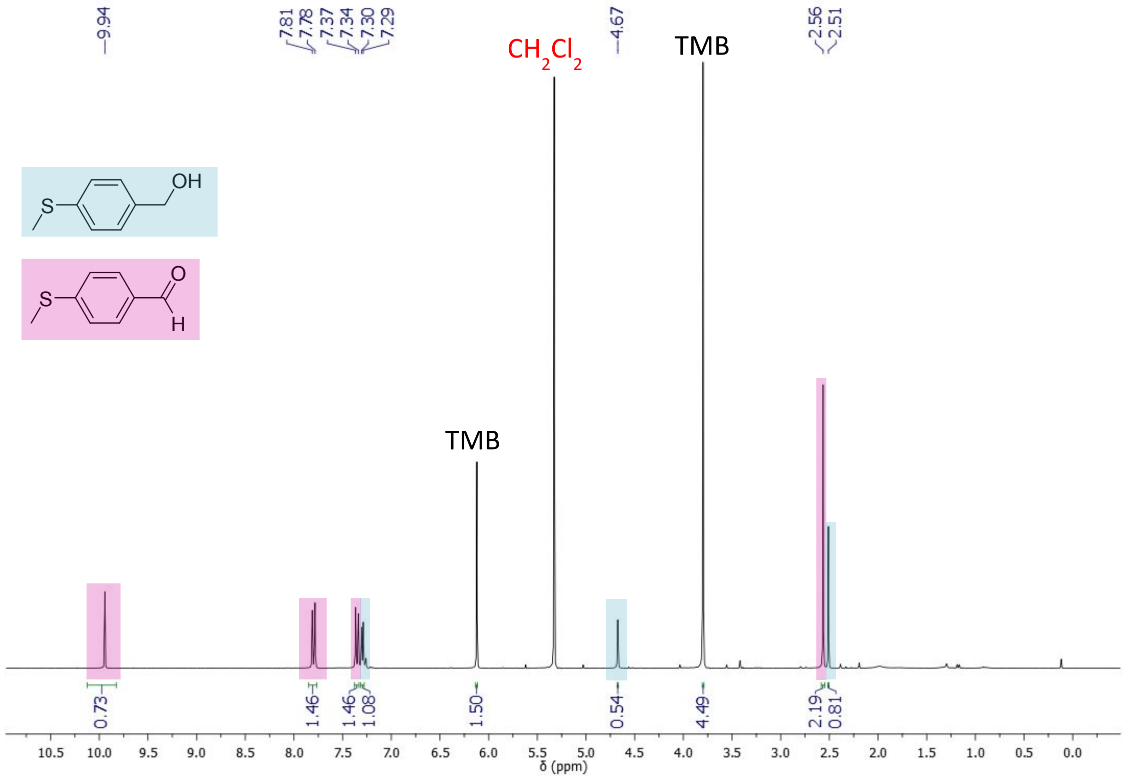

Figure S41 ^{1}H NMR (in $CDCl_3$) spectrum of *p*-(methylthio)benzaldehyde (Table 2, entry 12) in the presence of 0.25 mmol of 1,3,5-trimethoxybenze as an external standard (6.1 ppm and 3.75 ppm).

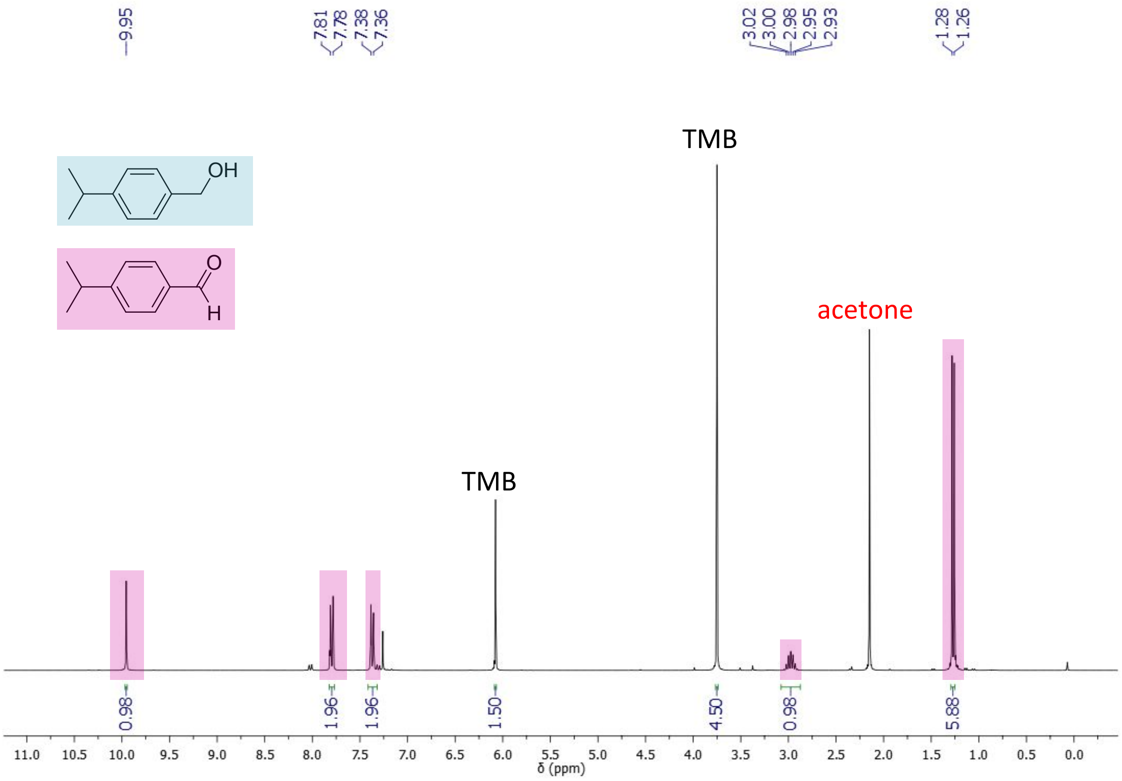

Figure S42 ^{1}H NMR spectrum of *p*-isopropylbenzaldehyde (Table 2, entry 13) in $CDCl_3$ in the presence of 0.25 mmol of TMB as an external standard (6.1 ppm and 3.75 ppm).

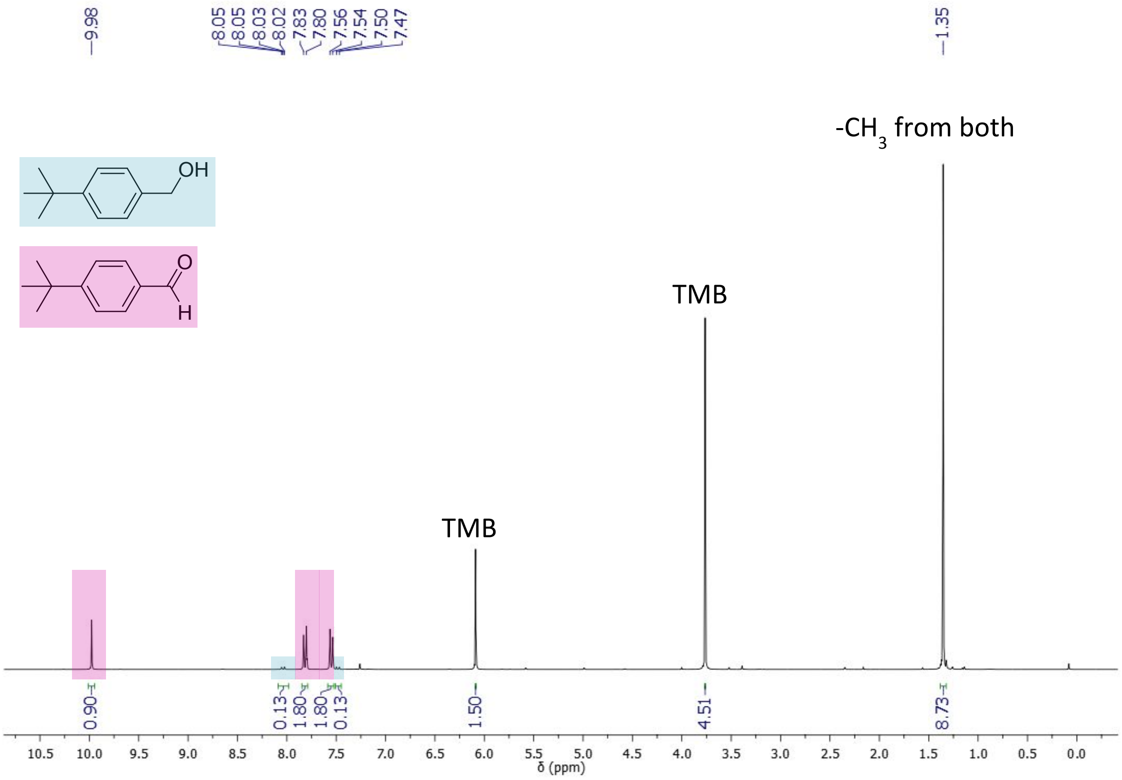

Figure S43 ^{1}H NMR spectrum of *p*-tertbutylbenzaldehyde (Table 2, entry 14) in $CDCl_3$ in the presence of 0.25 mmol of TMB as an external standard (6.1 ppm and 3.75 ppm).

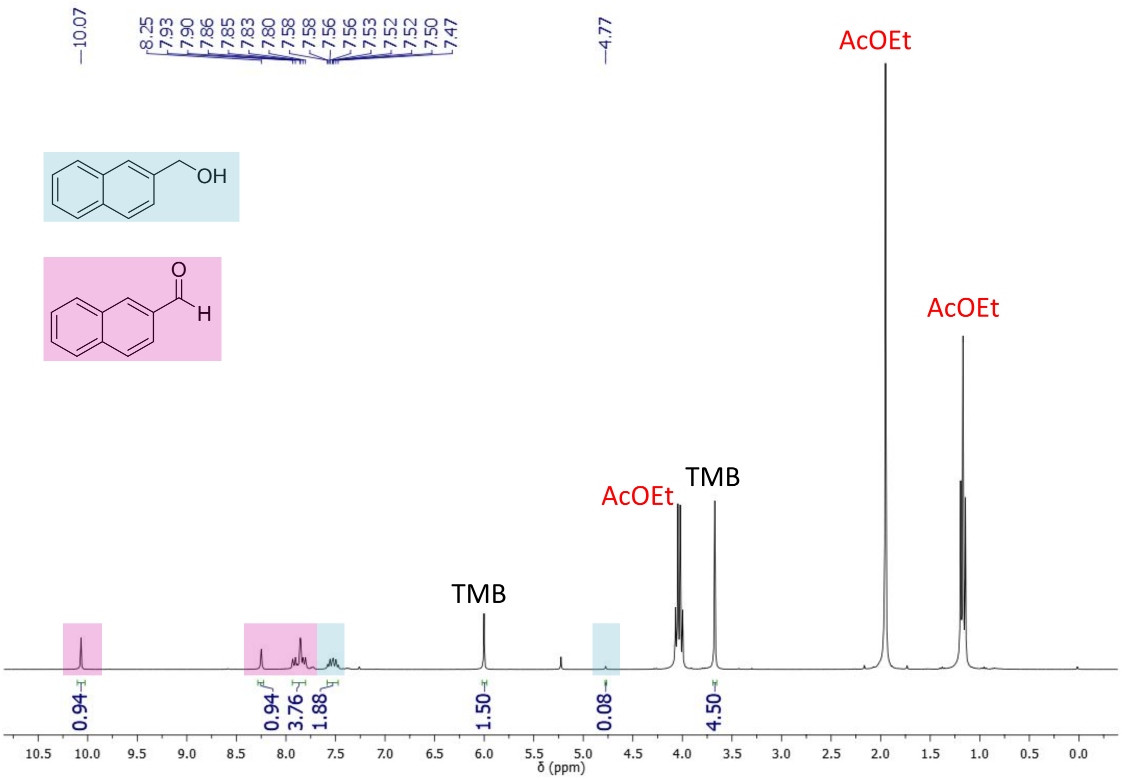

Figure S44 ^{1}H NMR spectrum of 2-naphtaldehyde (Table 2, entry 15) in $CDCl_3$ in the presence of 0.25 mmol of TMB as an external standard (6.1 ppm and 3.75 ppm).

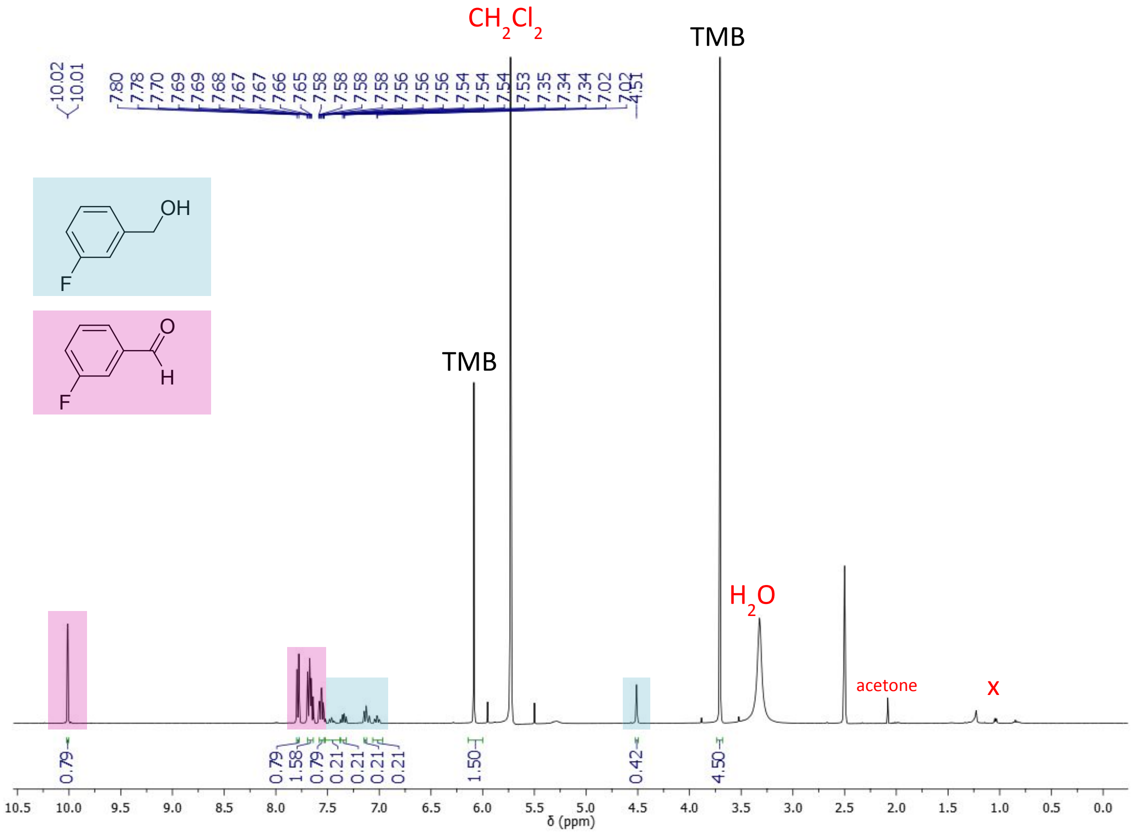

Figure S45 ^{1}H NMR spectrum *m*-fluorobenzaldehyde (Table 2, entry 16) in DMSO-d6 in the presence of 0.25 mmol of TMB as an external standard (6.1 ppm and 3.75 ppm).

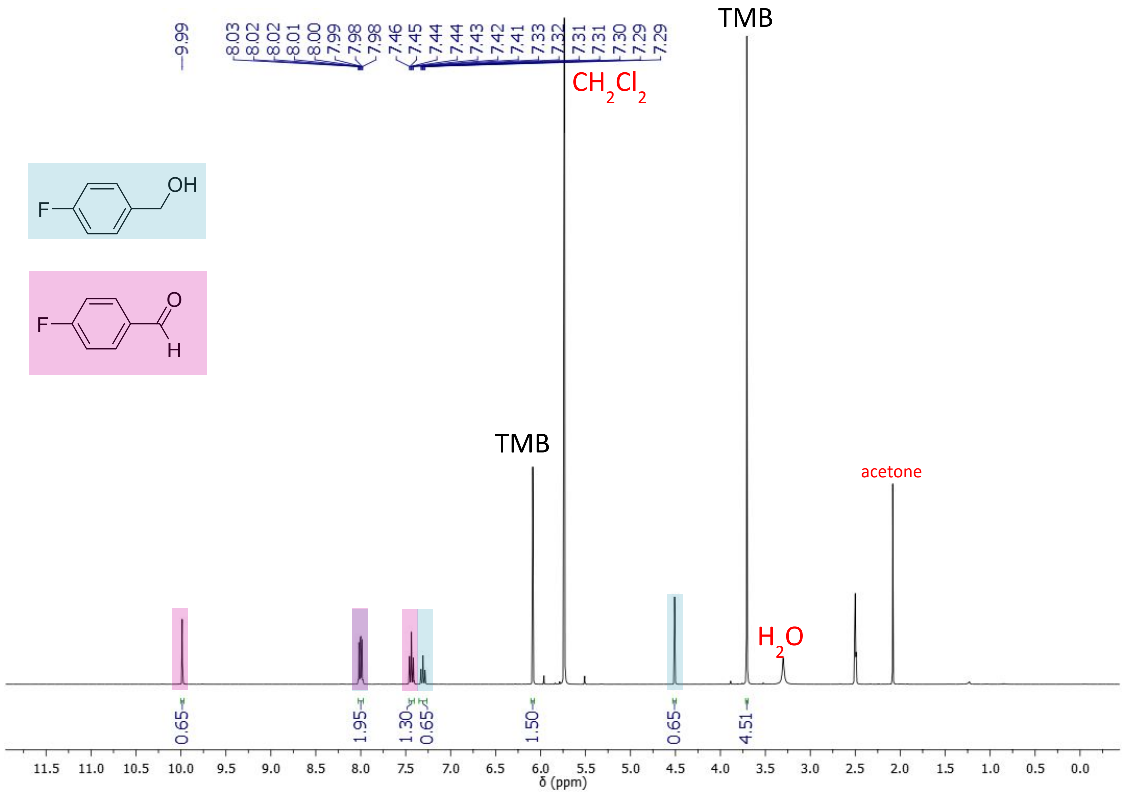

Figure S46 ^{1}H NMR spectrum *p*-fluorobenzaldehyde (Table 2, entry 17) in DMSO-d6 in the presence of 0.25 mmol of TMB as an external standard (6.1 ppm and 3.75 ppm).

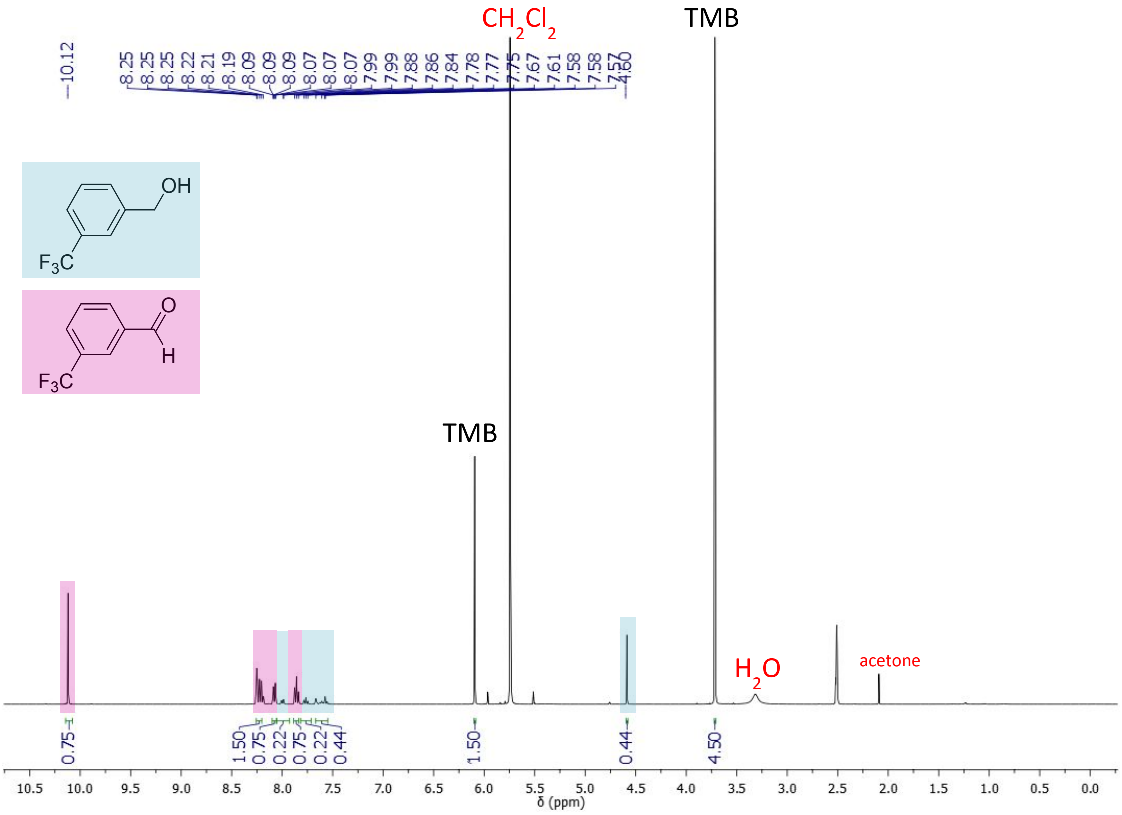

Figure S47 ^{1}H NMR spectrum *m*-trifluoromethyl benzaldehyde (Table 2, entry 18) in DMSO-d6 in the presence of 0.25 mmol of TMB as an external standard (6.1 ppm and 3.75 ppm).

S11. ^{1}H NMR spectra of crude reaction products in ODH of secondary alcohols

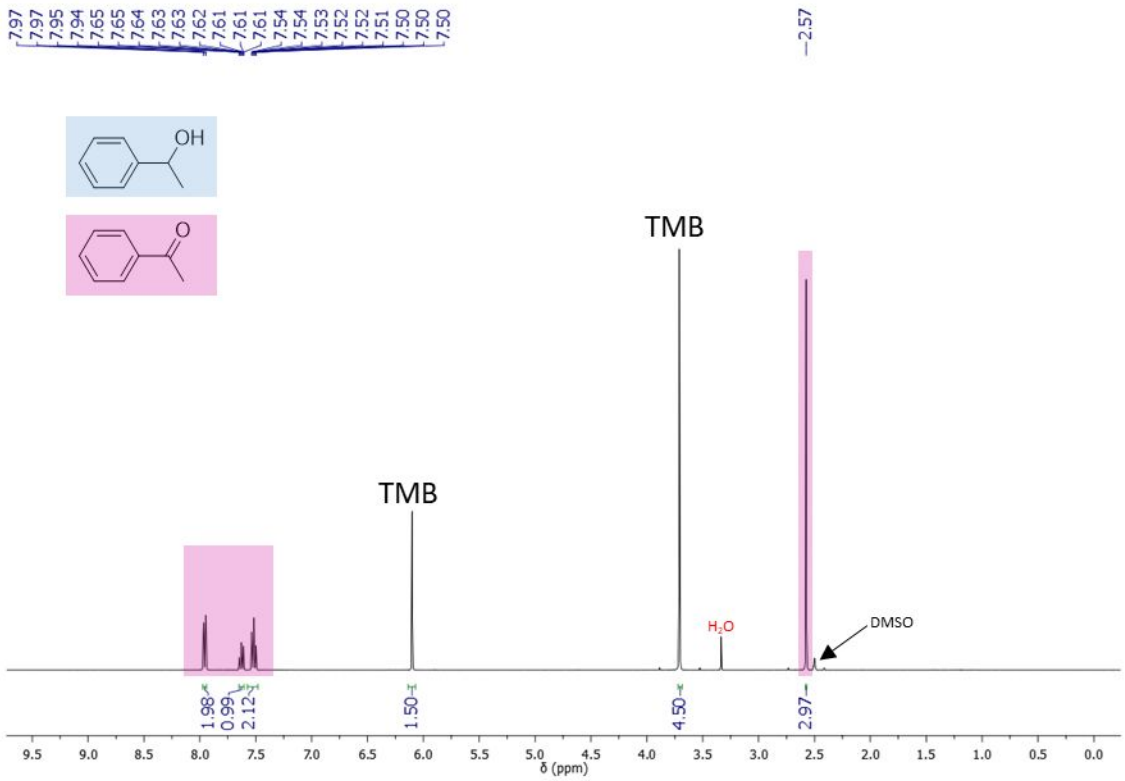

Figure S48 ^{1}H NMR (DMSO-d6) spectrum of acetophenone (Table 3, entry 1) in the presence of 0.25 mmol of 1,3,5-trimethoxybenze as an external standard (6.1 ppm and 3.75 ppm).

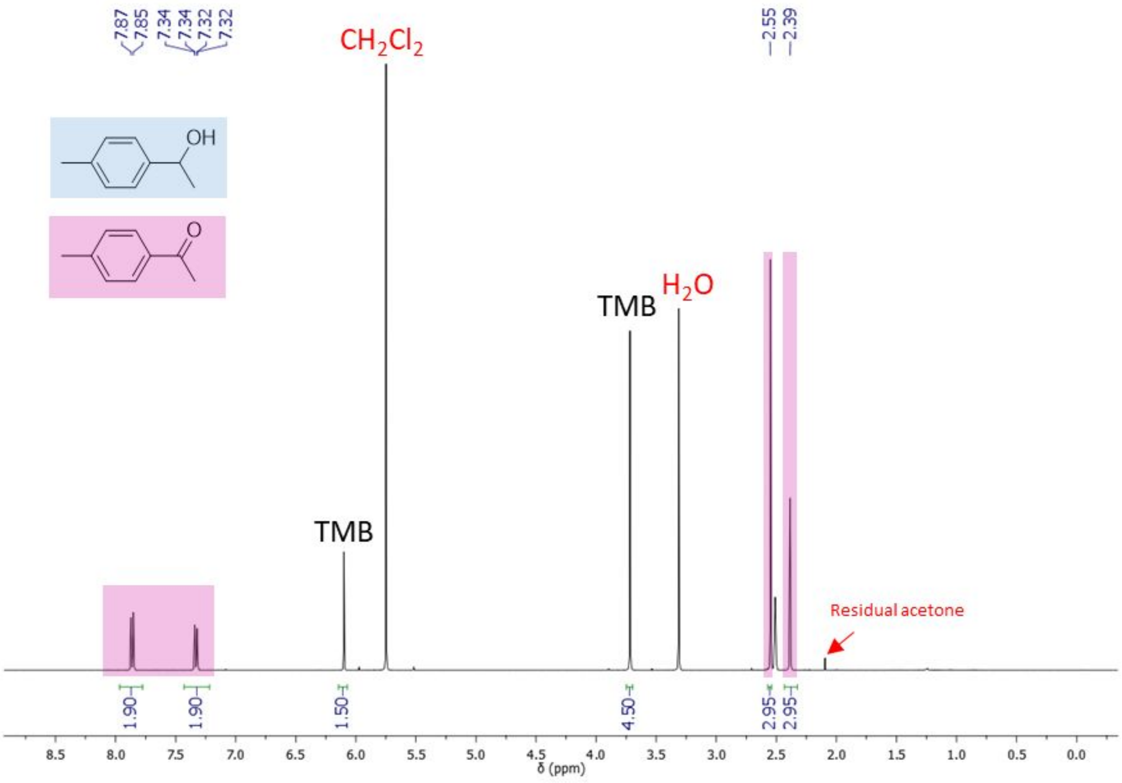

Figure S49 ^{1}H NMR (DMSO-d6) spectrum of 4-methyl-acetophenone (Table 3, entry 2) in the presence of 0.25 mmol of 1,3,5-trimethoxybenze as an external standard (6.1 ppm and 3.75 ppm).

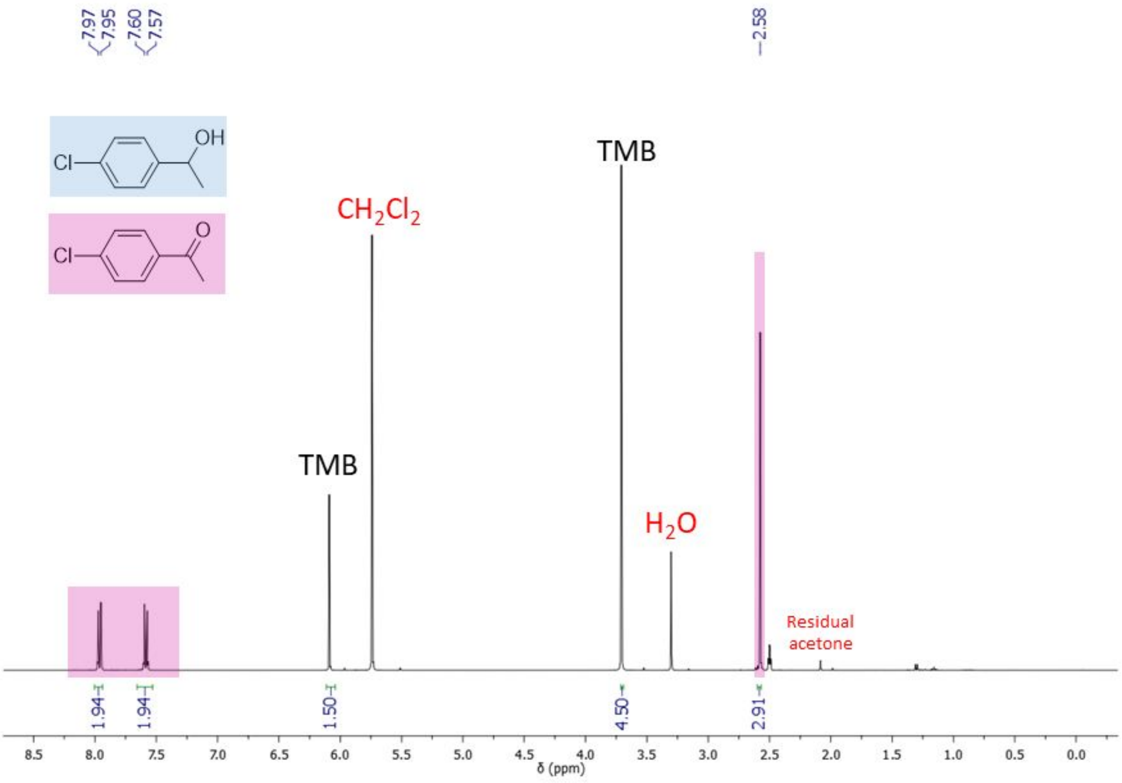

Figure S50 ^{1}H NMR (DMSO-d6) spectrum of 4-Chloro-acetophenone (Table 3, entry 3) in the presence of 0.25 mmol of 1,3,5-trimethoxybenze as an external standard (6.1 ppm and 3.75 ppm).

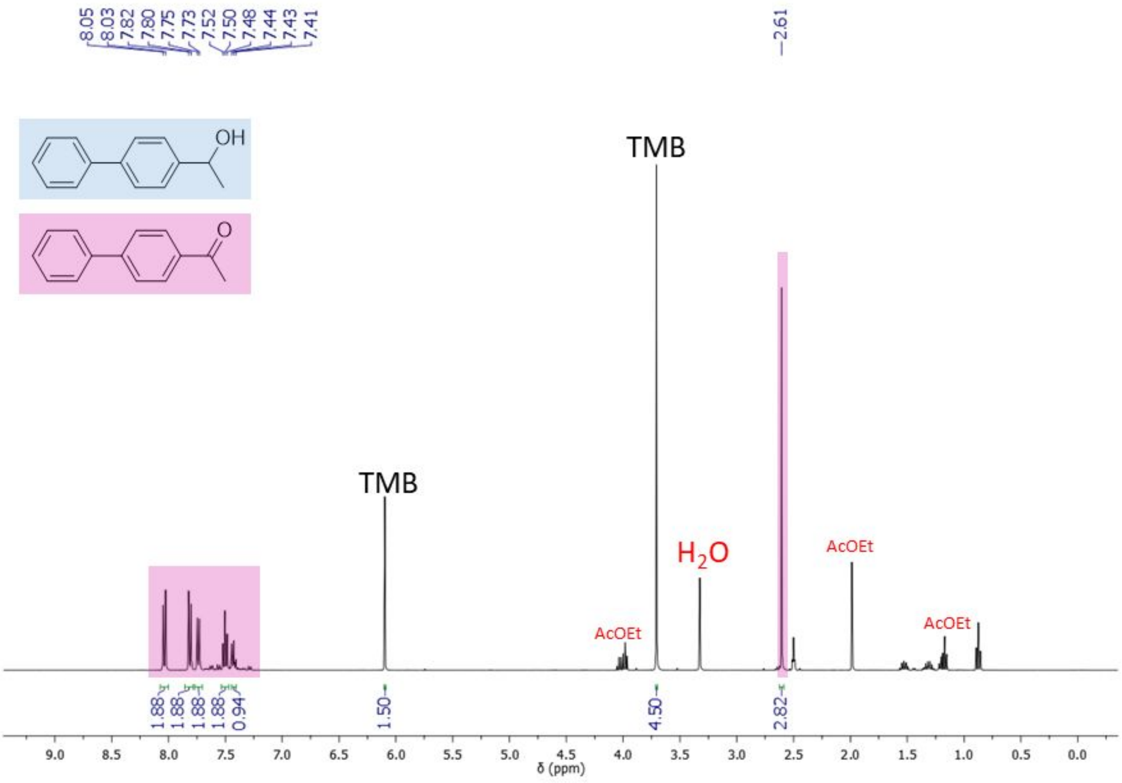

Figure S51 ^{1}H NMR (DMSO-d6) spectrum of 4-Phenyl-acetophenone (Table 3, entry 4) in the presence of 0.25 mmol of 1,3,5-trimethoxybenze as an external standard (6.1 ppm and 3.75 ppm).

S12. Chemoselectivity studies

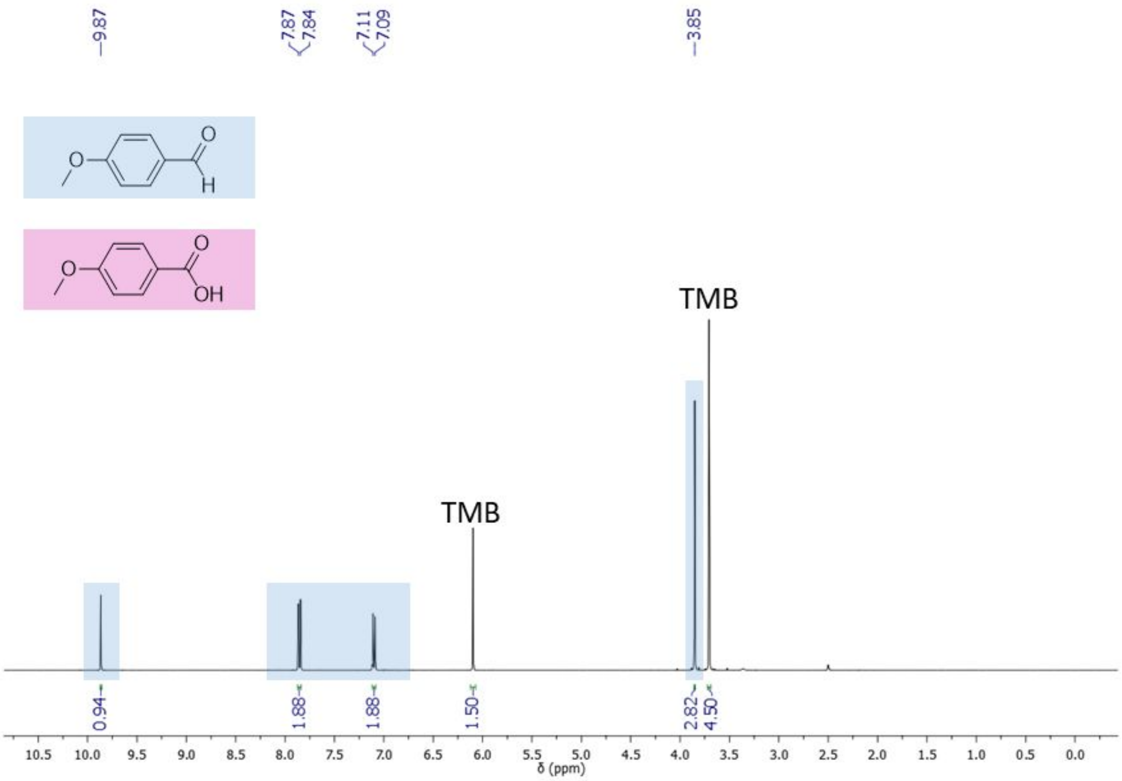

Figure S52 ^{1}H NMR (DMSO-d6) spectrum of *p*-methoxybenzaldehyde (Figure 4, reaction a) in the presence of 0.25 mmol of 1,3,5-trimethoxybenze as an external standard (6.1 ppm and 3.75 ppm).

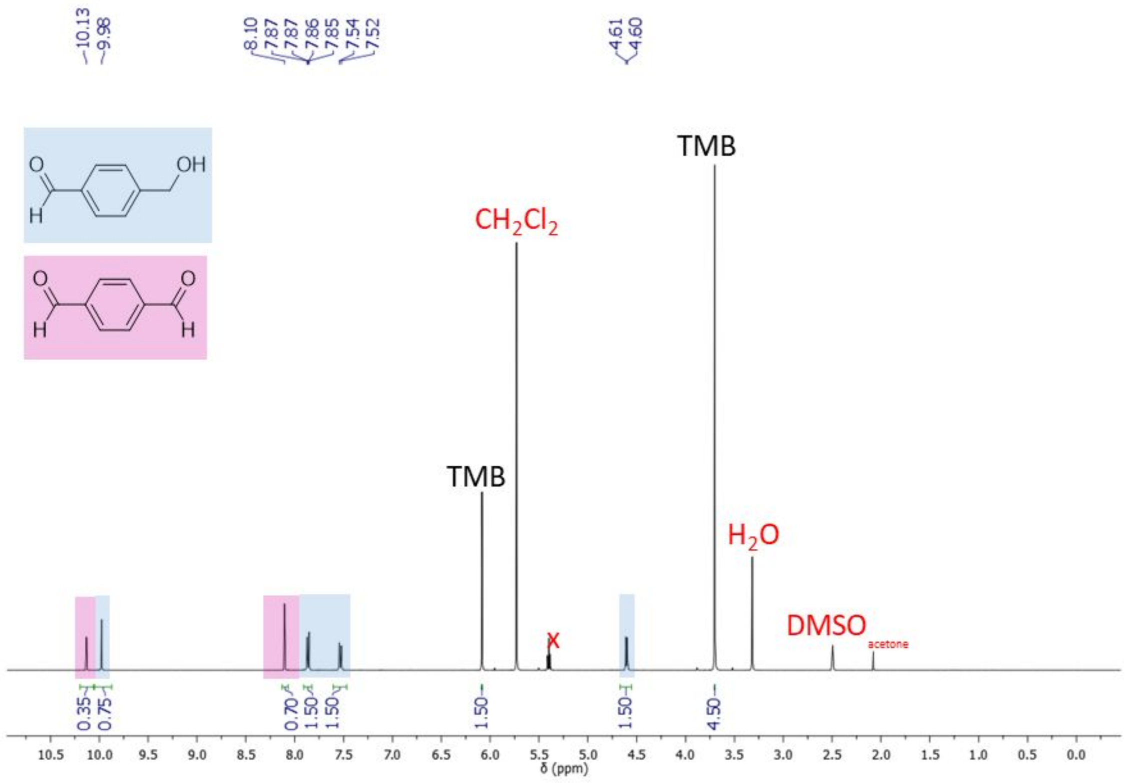

Figure S53 ^{1}H NMR (DMSO-d6) spectrum of terephthalaldehyde and starting material (Figure 4, reaction b) in the presence of 0.25 mmol of 1,3,5-trimethoxybenze as an external standard (6.1 ppm and 3.75 ppm).

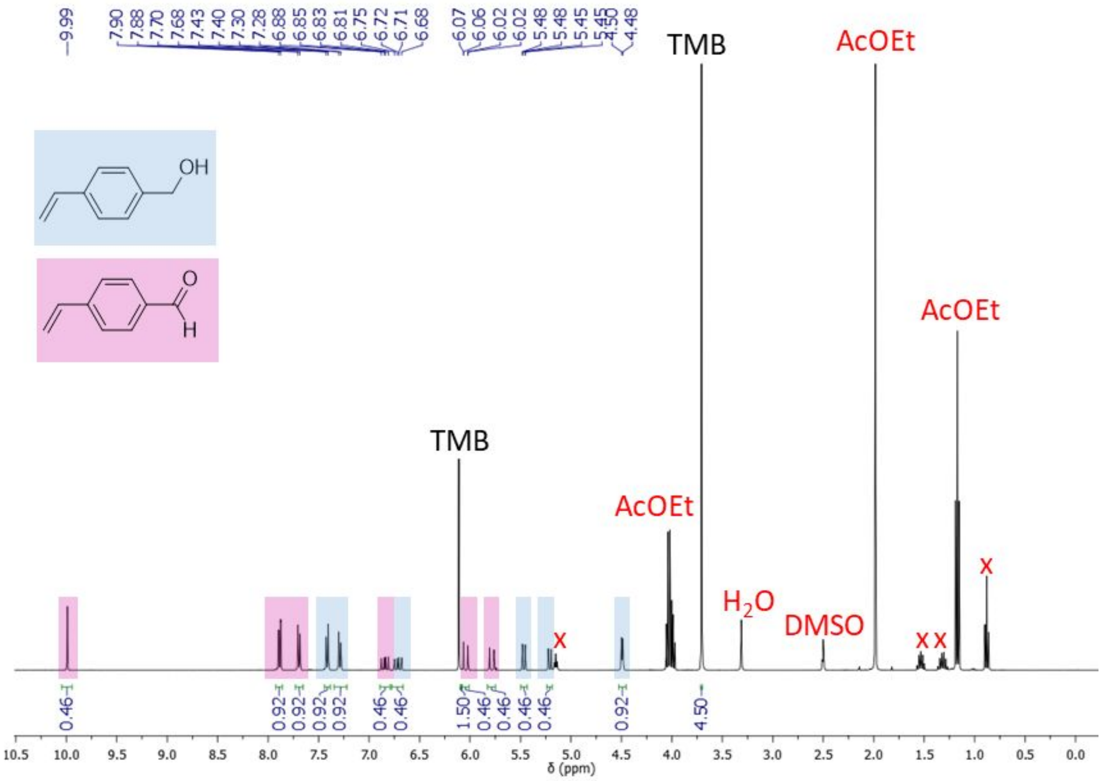

Figure S54 ^{1}H NMR (DMSO-d6) spectrum of *p*-vinylbenzaldehyde and starting material (Figure 4, reaction c) in the presence of 0.25 mmol of 1,3,5-trimethoxybenze as an external standard (6.1 ppm and 3.75 ppm).

S13. Hot filtration experiment

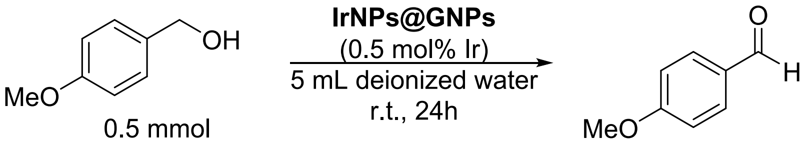

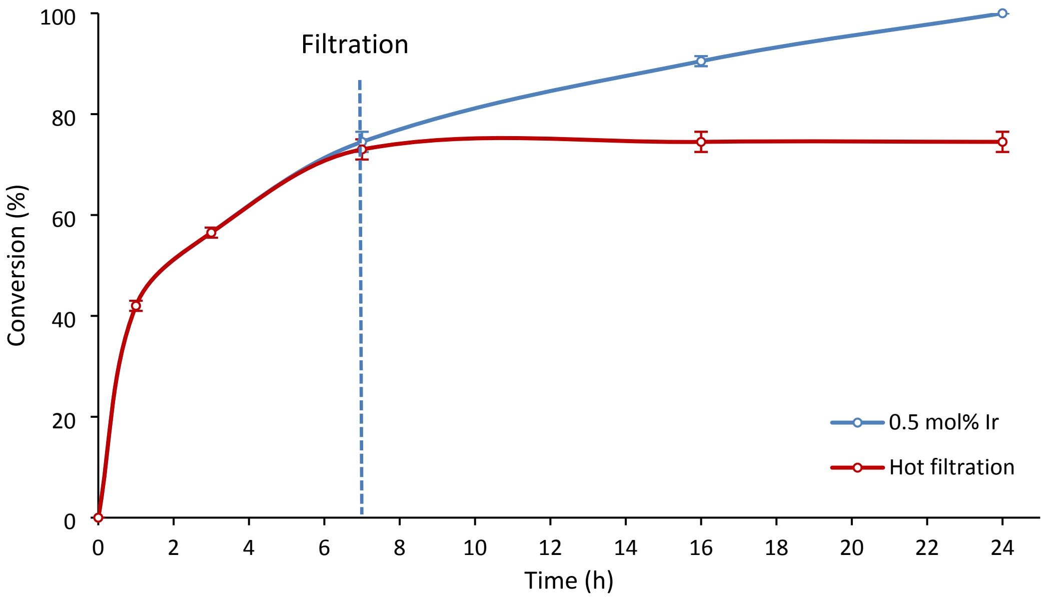

Figure S55 Hot filtration experiment demonstrating that catalytic activity in the filtrate is negligible after removal of the solid catalyst. (Blue: control experiment and Red: hot filtration experiment).

S14. Evaluation of green chemistry aspects

The sustainability in the ODH of alcohols was assessed using benzyl alcohol as a model substrate (Scheme S1). The key points in the sustainability of our protocol are the oxidant, solvent, temperature and catalyst. The oxidant used is atmospheric oxygen that is a bening reagent and only forms water as a byproduct. The reaction is carried out using water as solvent that is the most beneficial solvent from a green perspective. The reaction is performed at room temperature favouring the energetic metrics. There is a limitation in the use of a scarce metal source such as iridium. However, we have assessed the recycling and reuse of the catalytic material and even developed a reactivation protocol.

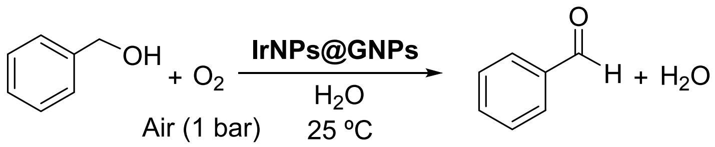

Scheme S1 Oxydation of alcohols by iridium nanoparticles anchored onto graphene.

Table S2 Data of components used for our catalytic transformation

Entry	Substance	CAS	MW g/mol	Price[a] €/g	NFPA 704[b]	NFPA ranking[c]	Specification
1	Benzyl alcohol	100-51-6	108.1	0.07	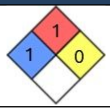	2	Reagentplus@ 99%
2	O_2 form air	-	32	-	-	-	Oxygen is not considered as it is used directly for air at 1 bar.
3	Benzaldehyde	100-51-6	106.1	0.04	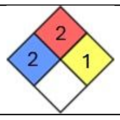	5	Reagentplus@ 99%
4	Water	7732-18-5	18	-	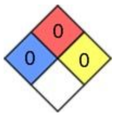	0	Price of water is not considered for economic calculations

[a]Prices were taken form online Merck catalog for Spain using the packing size of 2.5 Kg (or L) or closest packaging size if not avaiable. [b]NFPA 704: Standard system for the identification of the hazards of materials for emergency response is a standard maintained by the U.S.-based National Fire Protection Association. [c]NFPA ranking: is the sum of values provided in the NFPA 704 labels.

The green chemistry metrics were evaluated using the following formulas:

Atom economy	
$\mathrm{AE} = \frac{\mathrm{MW\ of\ desired\ product}}{\mathrm{MW\ of\ reagent\ A} + \mathrm{MW\ of\ reagent\ B}}\ \mathrm{x\ 100\%}$	AE = 85%
Comments:<br>The atom economy is below the ideal value (100 %) because of the formation of water as by product. However, water is considered as a bening by produc.	
Atom efficiency	
$\mathrm{AEff} = \mathrm{AE\ x\ yield(\%)}$	AE_{ff} = 80.7%
Comments:<br>Considering an avarage yield of 95%.	
Reaction mass efficiency	
$\mathrm{RME} = \frac{\mathrm{Kg\ of\ product}}{\mathrm{Kg\ of\ reagent\ A} + \mathrm{Kg\ of\ reagent\ B}}\ \mathrm{x\ 100\%}$	RME = 93%
Comments:<br>Water and oxygen from air are ideal solvent/reagent from a sustainable point of view and are not considered harmless.	
Economics	
$\mathrm{Eco} = \frac{\mathrm{product\ value\ per\ mol\ x\ yield(\%)}}{\mathrm{Reagent\ cost\ per\ mol\ x\ 100\%\%}}$	Eco = 0.5
Comments:<br>The economic value is low because the actual price of benzaldehyde is relatively low.	

S15. Catalyst comparison data

Table S3 Data comparison for the oxydative dehydrogenation of benzyl alcohol.[a]

Entry	Catalyst	Catalyst (mg)	Substrate (mmol)	Solvent	Temp (°C)	Time (h)	Conv. (%)	Sel. (%)	Additive	Ref.
1	Ru@Al_2O_3	200 (2 mol% Ru)	9.6	-	90	24	62	100	-	[7]
2	IrCpCl@MOF	(3 mol% Ir)	0.33	Toluene	110	24	54		Cs_2CO_3 Iodobenzene	[8]
3	Pd@SiO_2/Al_2O_3	100 (0.5 mol% Pd)	48.5	-	70	97	98		-	[9]
4	Cit-IrNPs	9	0.1	H_2O	25	10	92		-	[10]
5	Au/Pd@TiO_2	20	18.5	-	120	1	56	74	-	[11]
6	Ir@BPyOH-BP-PMO	(0.5 mol% Ir)	1.0	Toluene	110	18	95		-	[12]
7	IrNPs@NiO	(0.11 mol% Ir)	1.0	Toluene	110	9	84		-	[13]
8	AuIrNPs@Al_2O_3	30 (0.1 mol% Ir)	2.9	Toluene	80	2	60		K_2CO_3	[14]
9	$IrCl_3$/GO		0.48	Toluene		2	96		Ultrasounds	[15]
10	IrNPs@TiO_2	75 (5 mol% Ir)	1.5	Toluene	80	1	77		-	[16]
11	IrNPs@GNPs	15 (0.75 mol% Ir)	0.5	H_2O	25	24	97	100	-	This work

[a]A detailed and excact comparison between different catalytic systems is difficult due to the variability of reaction conditions and catalytic materials. However, there are few reports using water as reaction solvent and in most cases high temperatures are requiered.

S16. References

(1) Small Metal Particles and Supported Metal Catalysts. In *Metal-Catalysed Reactions of Hydrocarbons*; Springer US; pp 35–91. https://doi.org/10.1007/0-387-26111-7_2.

(2) Burés, J. A Simple Graphical Method to Determine the Order in Catalyst. *Angew. Chem. Int. Ed.* **2016**, *55* (6), 2028–2031. https://doi.org/10.1002/anie.201508983.

(3) Martínez-Carrión, A.; Howlett, M. G.; Alamillo-Ferrer, C.; Clayton, A. D.; Bourne, R. A.; Codina, A.; Vidal-Ferran, A.; Adams, R. W.; Burés, J. Kinetic Treatments for Catalyst Activation and Deactivation Processes Based on Variable Time Normalization Analysis. *Angew. Chem. Int. Ed.* **2019**, *58* (30), 10189–10193. https://doi.org/10.1002/anie.201903878.

(4) Nielsen, C. D. T.; Burés, J. Visual Kinetic Analysis. *Chem. Sci.* **2019**, *10* (2), 348–353. https://doi.org/10.1039/C8SC04698K.

(5) Rivero-Crespo, M.; Oliver-Meseguer, J.; Kapłońska, K.; Kuśtrowski, P.; Pardo, E.; Cerón-Carrasco, J. P.; Leyva-Pérez, A. Cyclic Metal(Oid) Clusters Control Platinum-Catalysed Hydrosilylation Reactions: From Soluble to Zeolite and MOF Catalysts. *Chem. Sci.* **2020**, *11* (31), 8113–8124. https://doi.org/10.1039/D0SC02391D.

(6) Burés, J.; Larrosa, I. Organic Reaction Mechanism Classification Using Machine Learning. *Nature* **2023**, *613* (February 2022), 689–695. https://doi.org/https://doi.org/10.1038/s41586-022-05639-4.

(7) Aneggi, E.; Campagnolo, F.; Zuccaccia, D.; Baratta, W.; Llorca, J.; Trovarelli, A. Sustainable Solvent-Free Selective Oxidation of Benzyl Alcohol Using Ru(0) Supported on Alumina. *Inorganics* **2023**, *11* (5), 177. https://doi.org/10.3390/inorganics11050177.

(8) Abednatanzi, S.; Derakhshandeh, P. G.; Abbasi, A.; Van Der Voort, P.; Leus, K. Direct Synthesis of an Iridium(III) Bipyridine Metal–Organic Framework as a Heterogeneous Catalyst for Aerobic Alcohol Oxidation. *ChemCatChem* **2016**, *8* (23), 3672–3679. https://doi.org/10.1002/cctc.201600985.

(9) Chen, J.; Zhang, Q.; Wang, Y.; Wan, H. Size-Dependent Catalytic Activity of Supported Palladium Nanoparticles for Aerobic Oxidation of Alcohols. *Adv. Synth. Catal.* **2008**, *350* (3), 453–464. https://doi.org/10.1002/adsc.200700350.

(10) Jin, G.; Liu, J.; Wang, C.; Gu, W.; Ran, G.; Liu, B.; Song, Q. Ir Nanoparticles with Multi-Enzyme Activities and Its Application in the Selective Oxidation of Aromatic Alcohols. *Appl. Catal. B Environ.* **2020**, *267* (January), 118725. https://doi.org/10.1016/j.apcatb.2020.118725.

(11) Sankar, M.; He, Q.; Morad, M.; Pritchard, J.; Freakley, S. J.; Edwards, J. K.; Taylor, S. H.; Morgan, D. J.; Carley, A. F.; Knight, D. W.; Kiely, C. J.; Hutchings, G. J. Synthesis of Stable Ligand-Free Gold–Palladium Nanoparticles Using a Simple Excess Anion Method. *ACS Nano* **2012**, *6* (8), 6600–6613. https://doi.org/10.1021/nn302299e.

(12) Shimizu, M.; Michikawa, K.; Maegawa, Y.; Inagaki, S.; Fujita, K. Iridium Complex Immobilized on Custom-Designed Periodic Mesoporous Organosilica as Reusable Catalyst for the Dehydrogenative Oxidation of Alcohols. *ACS Appl. Nano Mater.* **2020**, *3* (3), 2527–2535. https://doi.org/10.1021/acsanm.9b02607.

(13) Bordoloi, K.; Kalita, G. D.; Das, P. Iridium Nanoparticles on NiO as Heterogeneous Catalysts for Acceptorless Dehydrogenation of Primary Alcohols with Tunable Product Selectivity. *ACS Appl. Nano Mater.* **2023**, *6* (16), 14786–14797. https://doi.org/10.1021/acsanm.3c02324.

(14) Nagy, G.; Gál, T.; Srankó, D. F.; Sáfrán, G.; Maróti, B.; Sajó, I. E.; Schmidt, F.-P.; Beck, A. Selective Aerobic Oxidation of Benzyl Alcohol on Alumina Supported Au-Ru and Au-Ir Catalysts. *Mol. Catal.* **2020**, *492* (December 2019), 110917. https://doi.org/10.1016/j.mcat.2020.110917.

(15) Chen, T.-R.; Lin, Y.-S.; Wang, Y.-X.; Lee, W.-J.; Chen, K. H.-C.; Chen, J.-D. Graphene Oxide–Iridium Nanocatalyst for the Transformation of Benzylic Alcohols into Carbonyl Compounds. *RSC Adv.* **2020**, *10* (8), 4436–4445. https://doi.org/10.1039/C9RA10294A.

(16) Ito, S.; Wang, X.; Waheed, A.; Li, G.; Maeda, N.; Meier, D. M.; Naito, S.; Baiker, A. Support Effects in Iridium-Catalyzed Aerobic Oxidation of Benzyl Alcohol Studied by Modulation-Excitation Attenuated Total Reflection IR Spectroscopy. *J. Catal.* **2021**, *393*, 42–50. https://doi.org/10.1016/j.jcat.2020.11.010.
